# Oriented External Electric Fields Regurating the Reaction Mechanism of CH_4_ Oxidation Catalyzed by Fe(IV)-Oxo-Corrolazine: Insight from Density Functional Calculations

**DOI:** 10.3389/fchem.2022.896944

**Published:** 2022-06-29

**Authors:** Jie Wu, Tairen Long, Haiyan Wang, Jin-Xia Liang, Chun Zhu

**Affiliations:** School of Chemistry and Chemical Engineering, Guizhou University, Guiyang, China

**Keywords:** density functional calculations, Fe(IV)-Oxo-Corrolazine, CH_4_ oxidation, oriented external electric fields, catalysis

## Abstract

Methane is the simplest alkane and can be used as an alternative energy source for oil and coal, but the greenhouse effect caused by its leakage into the air is not negligible, and its conversion into liquid methanol not only facilitates transportation, but also contributes to carbon neutrality. In order to find an efficient method for converting methane to methanol, CH_4_ oxidation catalyzed by Fe(IV)-Oxo-corrolazine (Fe(IV)-Oxo-Cz) and its reaction mechanism regulation by oriented external electric fields (OEEFs) are systematically studied by density functional calculations. The calculations show that Fe(IV)-Oxo-Cz can abstract one H atom from CH_4_ to form the intermediate with OH group connecting on the corrolazine ring, with the energy barrier of 25.44 kcal mol^−1^. And then the product methanol is formed through the following rebound reaction. Moreover, the energy barrier can be reduced to 20.72 kcal mol^−1^ through a two-state reaction pathway. Furthermore, the effect of OEEFs on the reaction is investigated. We found that OEEFs can effectively regulate the reaction by adjusting the stability of the reactant and the transition state through the interaction of electric field-molecular dipole moment. When the electric field is negative, the energy barrier of the reaction decreases with the increase of electric intensity. Moreover, the OEEF aligned along the intrinsic Fe**‒**O reaction axis can effectively regulate the ability of forming the OH on the corrolazine ring by adjusting the charges of O and H atoms. When the electric field intensity is −0.010 a.u., the OH can be directly rebounded to the CH_3_· before it is connecting on the corrolazine ring, thus forming the product directly from the transition state without passing through the intermediate with only an energy barrier of 17.34 kcal mol^−1^, which greatly improves the selectivity of the reaction.

## 1 Introduction

Compared with oil or coal, methane is an environment-friendly energy, but it is also a greenhouse gas, and its greenhouse effect is much larger than that of carbon dioxide (Chong et al., 2016; Huang et al., 2018; Denning et al., 2021; Huang et al., 2021; Sánchez-López et al., 2021). A quarter of the greenhouse effect caused by man-made greenhouse gases is caused by the leakage of methane into the atmosphere (Kemp et al., 2016). In 2021, the United Nations called for a reduction in methane emission in the atmosphere, aiming to reduce global methane emission by 30% by the end of the century (Brenneis et al., 2022). Therefore, if an efficient method can be found to convert methane into methanol efficiently and economically, it can not only solve the difficulty of methane transportation, but also provide a large number of cheap raw materials for industrial production and reduce methane pipelines leakage (Park et al., 2019; Yan et al., 2020; Januario et al., 2021), thus providing feasible methods for methane emission reduction.

The high valent metal-oxygen systems have been characterized as key intermediates of heme and non heme enzymes (Solomon et al., 2000; Ogliaro et al., 2001; Meunier et al., 2004; Shaik et al., 2005; Rittle and Green, 2010; Visser et al., 2013; Adam et al., 2018; Huang and Groves, 2018; Dubey and Shaik, 2019; Ehudin et al., 2019; Cummins et al., 2020; Shaik and Dubey, 2021), which can effectively hydroxylate aliphatic hydrocarbons (Altun et al., 2007; Hazan et al., 2007), epoxidation (Niwa and Nakada, 2012; Nayak et al., 2020), halogenation (Liu and Groves, 2015), N-demethylation (Yang et al., 2018), and dehydrogenation reactions (Kumar et al., 2009). In particular, Fe(IV)-oxo porphyrin π-cation radical species, known as Cpd-I in heme proteins such as cytochrome P450, can mediate many key oxidative processes (Meunier et al., 2004; Shaik et al., 2005; Cho et al., 2012; Zhang et al., 2017; Caddell Haatveit et al., 2019; Caulfield et al., 2019). Corrolazines, formed by replacing the meso-position carbon atoms of corroles with N atoms, are very similar in structure to porphyrins, but have more π electrons than porphyrins, and can better stabilize high-valent metals (Ramdhanie et al., 2001; Goldberg et al., 2003; Fox et al., 2004; Lansky et al., 2005; Lansky and Goldberg, 2006; Goldberg 2007; McGown et al., 2009; Prokop et al., 2011; Pierloot et al., 2012; Baglia et al., 2015; Joslin et al., 2016; Jung et al., 2016; Zaragoza et al., 2016; Ghosh 2017; Zhu et al., 2018; Dedić et al., 2021; Wang et al., 2021; Zhu et al., 2021). As Fe is the active center metal of methane monooxygenase (Shteinman 2020; Freakley et al., 2021), which can selectively convert methane to methanol under natural environmental conditions. Fe-corrolazine is very likely to catalyze the oxidation of methane to methanol under very mild conditions. Therefore, it is necessary to study the oxidation of methane catalyzed by Fe-Oxo-corrolazine.

Recently, Sason Shaik et al. (Hirao et al., 2008; Gorin et al., 2012; Fried and Boxer, 2015; Li et al., 2015; Akamatsu et al., 2017; Che et al., 2018; Ciampi et al., 2018; He et al., 2018; Yu and Coote, 2019; Shaik et al., 2020; Shaik et al., 2004b; Kraskov et al., 2021; Yu et al., 2021; Zhang et al., 2021; de Visser et al., 2022) found that oriented external electric fields (OEEFs) can be used as a new type of catalyst to catalyze reactions by stabilizing transition states through the interactions between OEEFs and the molecular dipole moments, and even can increase the selectivity of reactions through adjusting the direction of OEEFs. Through theoretical calculations, our group also found that the OEEFs can modulate the reaction process through the interactions with the dipole moment of the reaction molecules (Wang et al., 2019).

Herein, we systematically study the reaction process of the oxidation of methane to methanol catalyzed by Fe(IV)-Oxo-corrolazine, and discuss the regulation of its reaction mechanism catalyzed by OEEFs, which provides a theoretical basis for the direct and efficient conversion of methane to methanol.

## 2 Computational Details

All the calculations were performed in Gaussian16 package (Frisch et al., 2016), using the B3LYP-D3(BJ) (Stephens et al., 1994; Grimme et al., 2010; Grimme et al., 2011) hybrid functional with the LANL2TZ (Roy et al., 2008) basis set coupled with the effective core potential for Fe atom and the all-electron 6–31++G (d,p) (Hariharan and Pople, 1973) basis set for other atoms. The structures of reactant (RC), transition state (TS), intermediate (INT) and product (P) were fully optimized without any symmetry constraints. Then the natures of these optimized structures were assessed by frequencies calculation, for RC, INT, and P with only real frequencies, and for TS with only one imaginary frequency. Moreover, all TS species were further verified by intrinsic reaction coordinate (IRC) calculations. The calculated output file was analyzed by Multiwfn to obtain the spin density (Lu and Chen, 2012).

**FIGURE 1:**
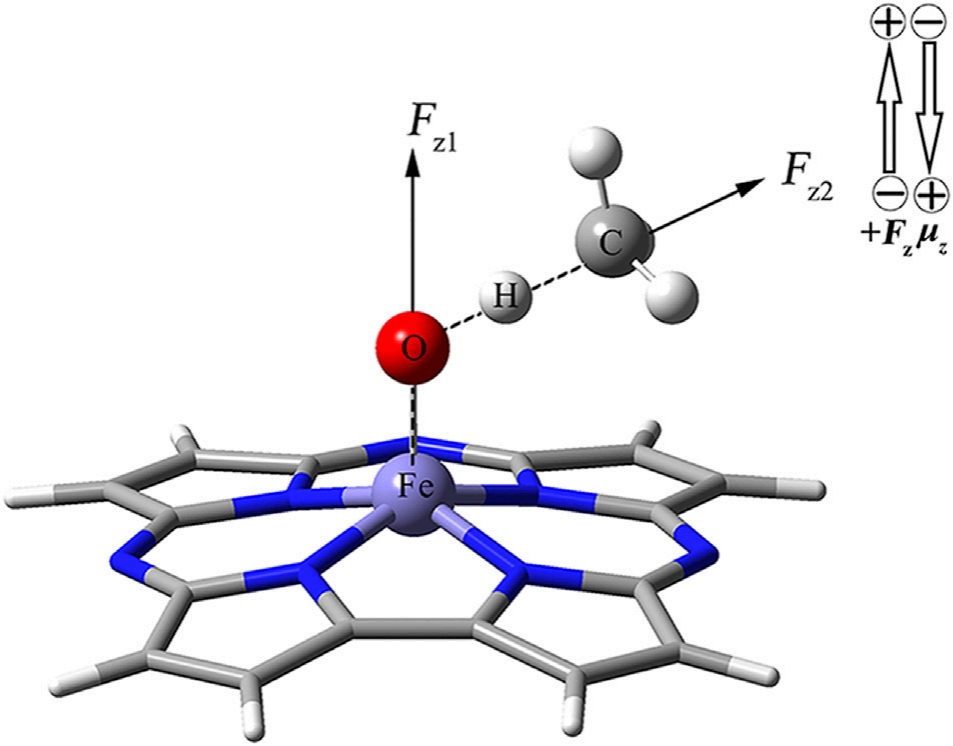
Definitions of two OEEFs *F*_z1_ is along the Fe‒O axis perpendicular to the corrolazine ring, and *F*_z2_ is along the O‒H axis.

As shown in Figure 1, using the keyword “Field = M ± N″, the two OEEFs, *F*_z1_ and *F*_z2_ along the Fe‒O axis and O‒H axis respectively, were applied to regulate the CH_4_ oxidation reaction catalyzed by Fe(IV)-Oxo-Cz. The positive direction of the electric field vector follows the Gaussian 16 convention, i.e., the direction from negative charge to positive charge is *F*_z_ > 0. The electric field intensity ranges from −0.010 a.u to +0.010 a.u. for *F*_z1_ and *F*_z2_ (1 a.u. = 51.4 V Å^−1^).

## 3 Results and Discussion

### 3.1 CH_4_ Oxidation Catalyzed by Fe(IV)-Oxo-Corrolazine Under the Field-Free Condition

#### 3.1.1 Structure and Electronic Properties of Fe(IV)-Oxo-Cz

**FIGURE 2:**
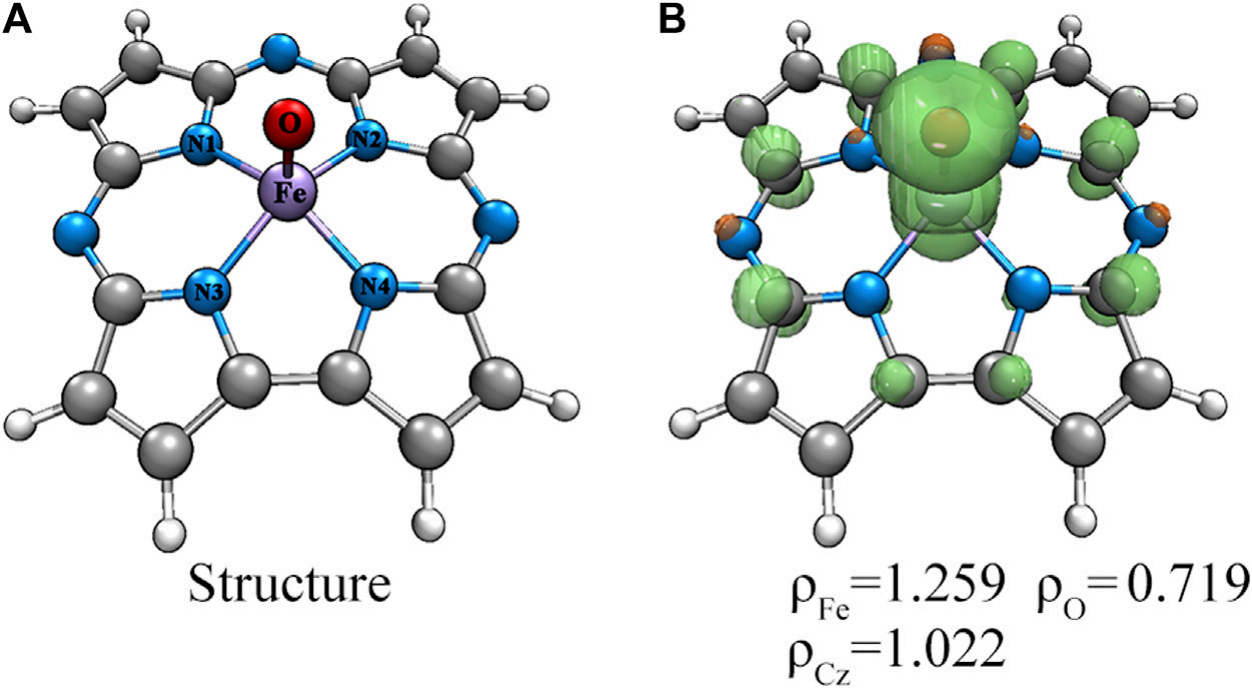
Structure **(A)** and spin densities **(B)** of the quartet Fe(IV)-Oxo-Cz.

**FIGURE 3:**
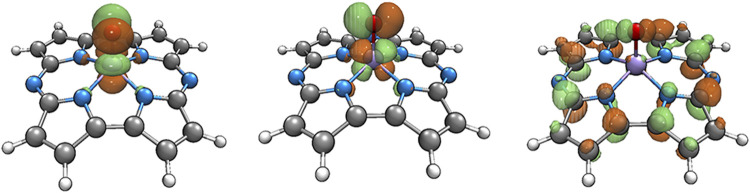
The single electron occupying molecular orbitals of the quartet Fe(IV)-Oxo-Cz.

The geometry structures of Fe(IV)-Oxo-Cz in doublet, quartet and sextet states were optimized and their relative energies and selective structure parameters are collected in Table 1. As seen from Table 1, the quartet state is lower in free energies than the double and sextet states by 10.25 and 40.05 kcal mol^−1^, respectively (absolute energies in Supplementary Table S1). Moreover, the calculated Fe**‒**O bond length in quartet state is 1.615 Å, which is close to the experimentally determined value of 1.640 Å (Cho et al., 2012). Therefore, the quartet state is the ground state, and due to the stronger interaction between the Fe atom and the O atom, the Fe atom deviates upward from the corrolazine ring, as shown in Figure 2. Furthermore, the spin densities and NPA charges for the different spin states of Fe(IV)-Oxo-Cz were calculated (see Supplementary Tables S2, S3). For the quartet state, the Fe**‒**O moiety only occupied two single electrons, and the remaining one single electron was occupied by the corrolazine ring as shown in Figure 2. And the single electron occupying molecular orbitals (SOMO) of the quartet state Fe(IV)-Oxo-Cz is shown in Figure 3. Two single electrons are in the orthogonal π orbitals of the Fe**‒**O moiety and the other mainly distributes on the corrolazine ring, which is exactly the same as the electronic configuration of Cpd-I (Huang and Groves, 2017; Zaragoza et al., 2017), reflecting the potential enzymatic catalytic activity of Fe(IV)-Oxo-Cz.

**TABLE 1:** Selected Bond Lengths (Å), Mulliken spin density of Fe, and relative energies (ΔG, kcal·mol^−1^) of Fe(IV)-Oxo-Cz, in doublet, quartet and sextet states.

States	d_Fe‒N1_	d_Fe‒N2_	d_Fe‒N3_	d_Fe‒N4_	d_Fe‒O_	ΔG	spin density
Doublet	1.895	1.895	1.892	1.892	1.568	10.25	0.905
Quarte	1.904	1.904	1.902	1.902	1.615	0	1.259
Sextet	1.910	1.910	1.899	1.899	1.612	40.05	1.253

#### 3.1.2 CH_4_ Oxidation Catalyzed by Fe(IV)-Oxo-Corrolazine Under the Field-free Condition

**FIGURE 4:**
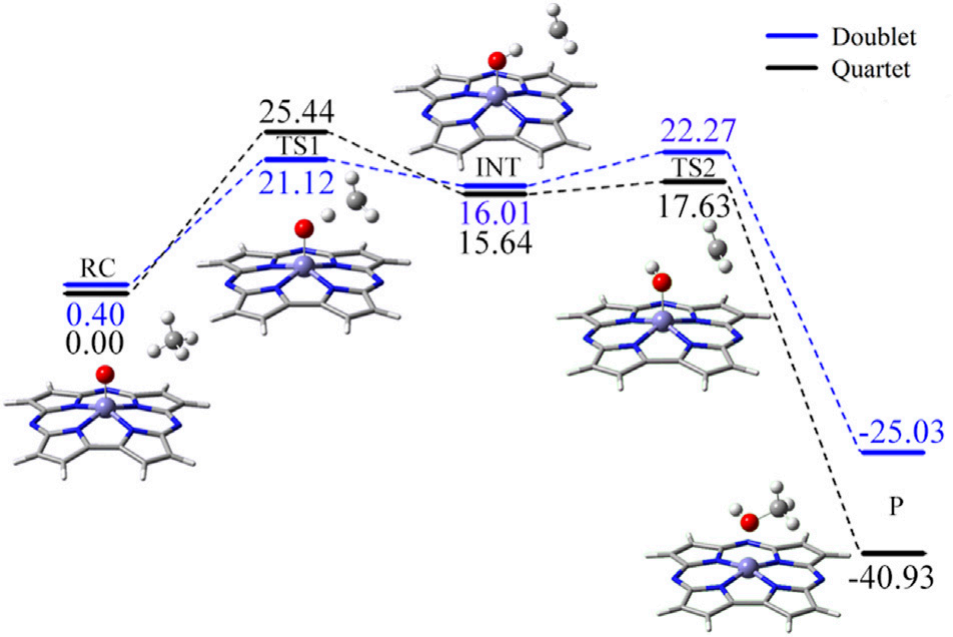
The predicted reaction pathway of CH_4_ oxidation catalyzed Fe(IV)-Oxo-Cz in the electric field free.

Based on the quartet ground state structure of Fe(IV)-Oxo-Cz, the reaction between Fe(IV)-Oxo-Cz and CH_4_ in quartet state in the absence of OEEF was studied. As illustrated in Figure 4; Table 2, the terminal O of Fe**‒**O first abstracts a hydrogen atom of CH_4_, and the bond length of Fe**‒**O bond increases from 1.615 Å of the reactant complex (RC) to 1.725 Å of the transition state (TS1), while the distance between O atom and H atom decreases significantly from 2.393 Å of RC to 1.187 Å of TS1 to yield O**‒**H bond, with the energy barrier of 25.44 kcal mol^−1^. With the progress of the reaction, the Fe**‒**O bond length grows gradually and the O**‒**H bond length further decreases, yielding the intermediates (INT) of Fe(IV)**‒**OH and CH_3_·. Then the product (P) CH_3_OH is produced through the rebound reaction where the newly formed OH is rapidly rebounded to the CH_3_· from Fe-corrolazine. (Cummins et al., 2020; Huang and Groves, 2017; Shaik et al., 2004a). And in this step, the reaction barrier is only 1.98 kcal mol^−1^, and the reaction energy of 40.93 kcal mol^−1^ will promote the reaction to the right.

**TABLE 2:** Selected Bond Lengths (Å) and the relative electronic energies (ΔG, kcal·mol^−1^) of species involved in the reaction of CH_4_ oxidation catalyzed for the quartet of Fe(IV)-Oxo-Cz.

Complexes	d_Fe‒O_	d_O‒H_	d_C‒H_	d_C‒O_	ΔG
RC	1.615	2.393	1.092	3.478	0.00
TS1	1.725	1.187	1.353	2.540	25.44
INT	1.780	0.979	2.166	3.142	15.64
TS2	1.811	0.975	2.906	2.880	17.63
P	2.186	0.968	1.998	1.445	-40.93

Moreover, considering that the reaction may proceed at different potential energy surfaces, we further calculated the double state potential energy surface. As shown in Figure 4 and Supplementary Table S4, the TS1 of the double state is lower than that of the quartet state, indicating that the reaction is a two-state reaction and the reaction is easier to carry out (Schröder et al., 2000; Stuyver et al., 2020). However, considering that the quartet state of RC is the ground state, and the quartet state of P is much more stable than the double state, and that when the OEEF with *F*_z1_ = −0.010 a.u. is applied, the energy order of the quartet and double states of reactant dose not changed and there is no energy crossing points along the reaction pathway, so we further study the effect of OEEFs on the reaction in quartet state in detail. For comparison, we also selected the representative external electric fields −0.010, −0.004, −0.002, +0.002, +0.004, and +0.010 a.u. for the calculations of the double state reaction, as shown in Supplementary Tables S5, S6.” As shown in Supplementary Table S7, the OEEF does not change the rate-determining step of the reaction.

### 3.2 OEEFs Regulating the Reaction Mechanism

#### 3.2.1 The Effect of OEEF on the Stabilities of the RC and TS1

**FIGURE 5:**
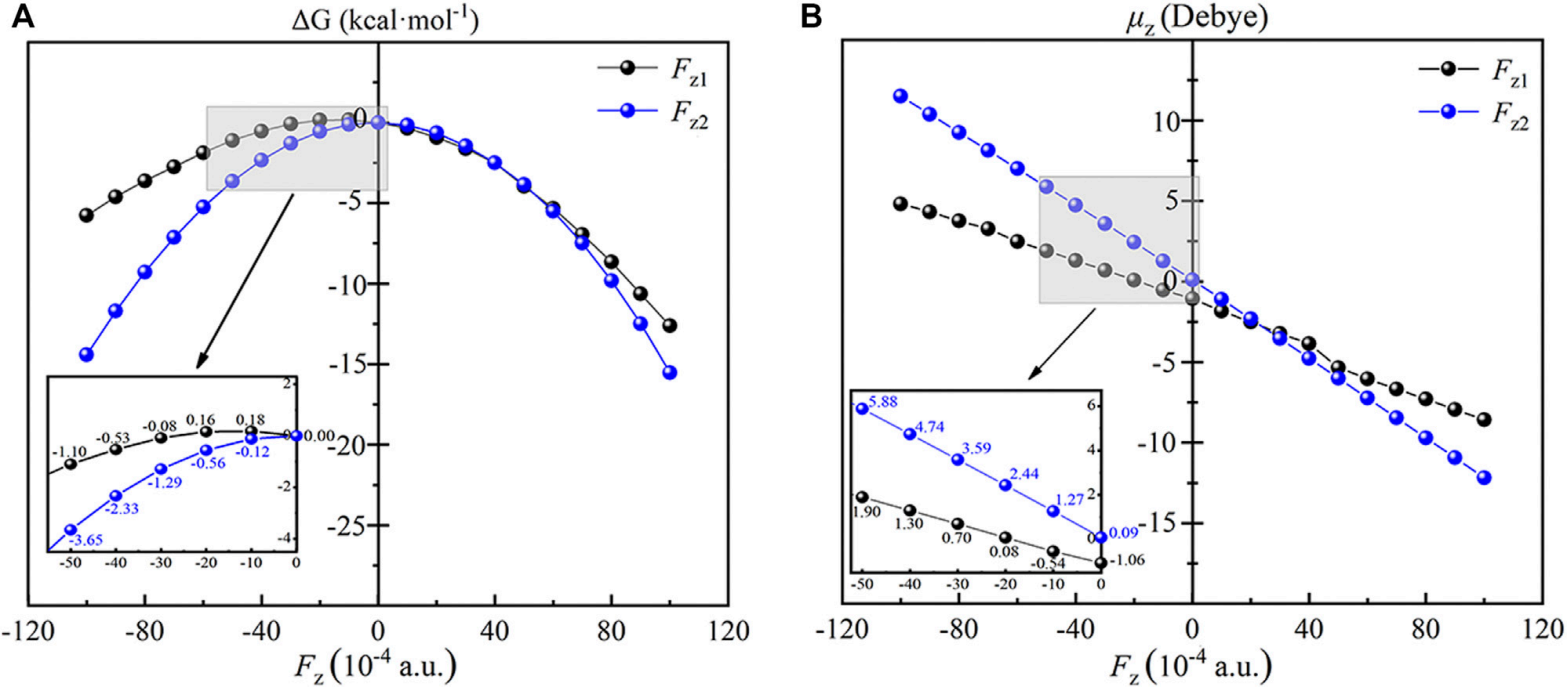
Plots of the relative energies **(A)** and the dipole moments **(B)** of the TS1 as a function of the applied OEEFs. The inset is the enlarged view at *F*_z1_ and *F*_z2_ = −0.005 a.u. to 0 a.u. black curve for *F*_z1_ and blue curve for *F*_z2_.

In order to explore the regulation mechanism of OEEFs in the reaction, we first systematically studied the effect of OEEFs on the TS1 of the reaction. As shown in Figure 5A, due to the application of OEEFs, the relative energies of the reaction TS1 change significantly. For *F*_z_ > 0, the structure of TS1 is stabilized by OEEFs, and its relative energy decreases with the increase of the electric field intensity. While for *F*_z_ < 0, OEEFs in different directions have different effects on the TS1. For *F*_z1_, when it is in the range of 0 ∼ −0.002 a.u., the TS1 is destabilized by the OEEF, and its relative energy increases with the increase of the electric field intensity. However, as the electric field intensity further increases more than -0.002 a.u. the TS1 is stabilized by the OEEF again, and its relative energy decreases with the increase of the electric field intensity. For *F*_z2_, the TS1 is always stabilized by the OEEF, and its relative energy decreases with the increase of the electric field intensity.

To explore the essential reason for the stability change of the TS1 effected by OEEFs, the dipole moments in z orientation of the TS1 of Fe(IV)-Oxo-Cz and CH_4_ at different electric field intensities are analyzed. As shown in Figure 5B, for *F*_z1_ > 0, the dipole moment in z1 direction of the TS1 increases from -1.06 D in the electric field free to -8.58 D in *F*_z1_ = +0.010 a.u. Therefore, the TS1 is stabilized by the applied OEEFs originating from the attractive interaction between the increased dipole moment in z1 direction and the OEEF. When the OEEF is reversed to *F*_z1_ < 0, the interaction between the dipole moment and the OEEF becomes complex. For −0.002 a.u. < *F*_z1_ < 0, the OEEF decreases the dipole moment of in z1 direction of the TS1, and the repulsion between *F*_z1_ and the dipole moment of the TS1 destabilizes the TS1. However, when *F*_z1_ becomes more negative, it flips the orientation of the molecular dipole of the TS1. Therefore, the increasing OEEF increases the dipole moment in z1 orientation of the TS1, thus again stabilizing the TS1 originating from the attraction between the increased dipole moment and *F*_z1_. For *F*_z2_, the orientation of molecular dipole moment in z2 direction is opposite to the direction of the OEEF, and the dipole moment always increases with the increase of electric field intensity, thus always stabilizing the TS1 of the retion originating from the attractive interaction between the increased dipole moment in z2 direction and the OEEF.

**FIGURE 6:**
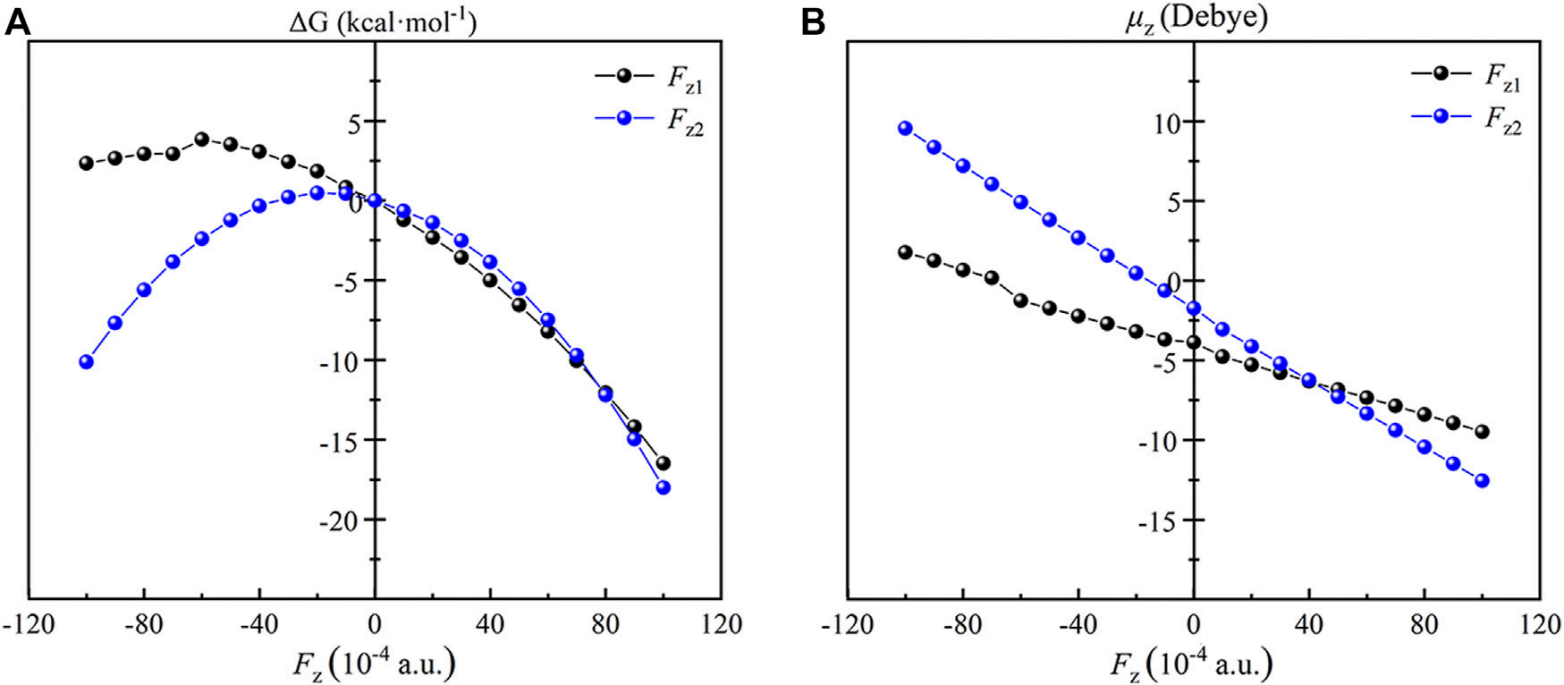
Plots of the relative energies **(A)** and the dipole moments **(B)** of the RC as a function of the applied OEEFs.

Similar to the TS1, the RC of the reaction of Fe(IV)-Oxo-Cz and CH_4_ are also effected by the OEEF remarkably. As shown in Figure 6, for *F*_z1_ and *F*_z2_ > 0, the dipole moment in z orientation of the RC increases with the increase of OEEFs. Therefore, the RC is stabilized by OEEFs, originating from the attraction between OEEFs and the increased dipole moment in z orientation. For *F*_z1_ and *F*_z2_ < 0, the dipole moment in z orientation first decreases with the increase of the electric field intensity in the initial part of *F*_z_ < 0, and then increases with the increase of the electric field intensity, which is more than -0.006 a.u. for *F*_z1_ and -0.002 a.u. for *F*_z2_ resulting from the reverse of the molecular dipole in z direction of the RC. Thus, the RC is first destabilized and then stabilized by OEEFs resulting from the repulsion and attraction between OEEFs and the increased dipole moment in z orientation, respectively.

#### 3.2.2 The Effect of OEEFs on the Energy Barrier of CH_4_ Oxidation Catalyzed by Fe(IV)-Oxo-Corrolazine

**FIGURE 7:**
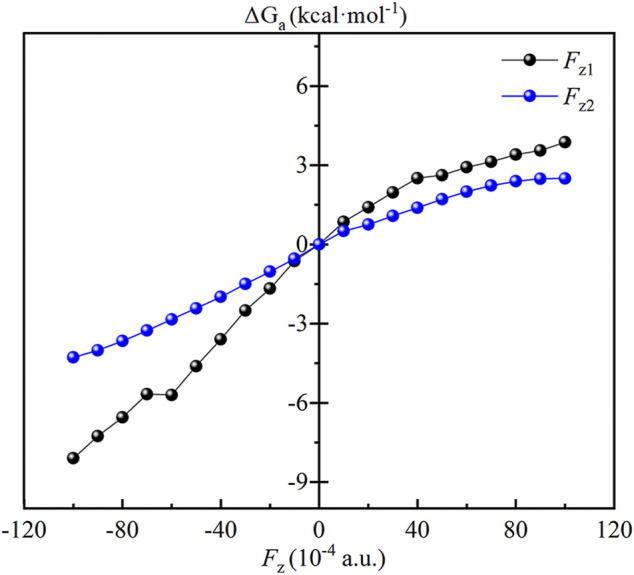
Plots of the relative energy barrier (ΔG_a_, kcal·mol^−1^) of the CH_4_ oxidation catalyzed Fe(IV)-Oxo-Cz.

The effect of OEEFs on the energy barrier of the reaction of CH_4_ oxidation by Fe(IV)-Oxo-Cz is further investigated. As shown in Table 3; Figure 7, for *F*_z1_ and *F*_z2_ > 0, the energy barriers of the reaction increase with the increase of electric field intensity, because the dipole moments in z direction of the RC and TS1 increase with the increase of electric field intensity, and the dipole moments of the RC is always larger than the TS1 in the electric intensity range, thus resulting in its stronger stabilization by OEEFs. However, for *F*_z1_ and *F*_z2_ < 0, the stabilization of the TS1 by OEEFs is always stronger than that of the RC, originating from the stronger dipole moment of the TS1, so the energy barriers of the reaction decrease with the increase of electric field intensity, in which the energy barriers decrease to 17.34 and 21.16 kcal mol^−1^ for *F*_z1_ = −0.010 and *F*_z2_ = −0.010 a.u., respectively, thus greatly promoting the reaction. Especially for *F*_z1_, it can more effectively regulate the reaction than *F*_z2_, resulting from its greater slope of energy barrier curve in Figure 7. Moreover, as its direction is nearly perpendicular to the corrolazine ring, *F*_z1_ can be more easily aligned, thus making it easier to apply in practice.

**TABLE 3:** The dipole moments of the RC, TS1 and the energy barrier (ΔG, kcal·mol^−1^) of CH_4_ oxidation catalyzed by Fe(IV)-Oxo-Cz under different electric field intensities.

*F*_z1_ (10^−4^ a.u.)	−100	−80	−60	−40	−20	0	20	40	60	80	100
*μ*_z1_ (RC)	1.76	0.66	−1.26	−2.23	−3.21	−3.89	−5.28	−6.31	−7.35	−8.40	−9.48
*μ*_z1_ (TS1)	4.82	3.76	2.48	1.30	0.08	−1.06	−2.52	−3.87	−6.04	−7.29	−8.58
ΔG_a1_	17.34	18.89	19.74	21.85	23.77	25.44	26.85	27.95	28.36	28.84	29.31
***F*_z2_ (10^−4^ a.u.)**	−100	−80	−60	−40	−20	0	20	40	60	80	100
*μ*_z2_ (RC)	9.55	7.20	4.92	2.68	0.46	−1.74	−4.13	−6.24	−8.34	−10.44	−12.56
*μ*_z2_ (TS1)	11.50	9.26	7.01	4.74	2.44	0.09	−2.32	−4.77	−7.23	−9.70	−12.18
ΔG_a2_	21.16	21.78	22.60	23.45	24.41	25.44	26.19	26.83	27.44	27.83	27.94

#### 3.2.3 OEEFs Optimizing the Process of CH_4_ Oxidation Catalyzed by Fe(IV)-Oxo-Corrolazine

As shown in Table 4, for *F*_z1_ < 0, with the increase of electric field intensity, the negative charge of O atom and the positive charge of H atom in the TS1 decrease, while the O**‒**H distance increases. Therefore, the ability of H to transfer to O to form stable the OH groups on the corrolazine ring decreases, which is the key process of forming the INT. When the intensity of the electric field reaches -0.010 a.u., both the negative charge of the O atom and the positive charge of the H atom reach the smallest, and the OH distance is the largest. Therefore, before the OH group forming on the corrolazine ring, it directly returns to the C atom through the rebound reaction from the P, as shown in Supplementary Figure S1, thereby simplifying the process of the reaction without passing through the INT to the product, thus avoiding the coupling between the intermediates to generate other products (Cho et al., 2012), greatly improving the selectivity of the reaction, and being beneficial to industrial applications.

**TABLE 4:** The O‒H Lengths (Å), the NPA charges (|e|) of the O and H atoms of the TS1 under different electric field intensities.

*F*_z1_ (10^–4^ a.u.)	−100	−80	−60	−40	−20	0
O	−0.402	−0.426	−0.449	−0.471	−0.494	−0.517
H	0.317	0.325	0.334	0.343	0.351	0.361
d_O**‒**H_	1.309	1.281	1.255	1.232	1.210	1.187
***Fz2 (10^–4^ a.u.)***	−100	−80	−60	−40	−20	0
O	−0.463	−0.474	−0.484	−0.494	−0.505	−0.517
H	0.325	0.331	0.338	0.345	0.353	0.361
d_O**‒**H_	1.282	1.263	1.245	1.226	1.207	1.187

## 4 Conclusion

Extensive density functional calculations have been carried out to explore the CH_4_ oxidation reaction catalyzed by Fe(IV)-Oxo-Cz and its regulatory mechanism by OEEFs. The calculations show one H atom of CH_4_ is captured by Fe(IV)-Oxo-Cz to form INT, in which OH group is connecting on the corrolazine ring, and then the product methanol is formed through the following rebound reaction. And the energy barrier of the reaction is 25.44 kcal mol^−1^. Moreover, the energy barrier can be reduced to 20.72 kcal mol^−1^ through a two-state reaction pathway. To facilitate the reaction, we applied OEEFs *F*_z1_ and *F*_z2_ along the Fe**‒**O axis and the O**‒**H axis to modulate the reaction, respectively. When the positive OEEFs are applied, the energy barrier of the reaction increases with the increase of the electric field intensity. However, while flipping OEEFs to the negative direction, the energy barrier of the reaction decreases with the increase of the electric field intensity originating from the interaction of electric field-molecular dipole moment, which can facilitate the reaction. Especially, the *F*_z1_ is easier be applied in practice because its direction is along the intrinsic Fe**‒**O reaction axis approximately perpendicular to the corrolazine ring, and it can effectively modulate the ability of forming the OH on the corrolazine ring by adjusting the charge of O and H atoms. When its intensity is −0.010 a.u., *F*_z1_ can simplify the reaction path to directly form the reaction product from the transition state without passing through the intermediate, with only an energy barrier of 17.34 kcal mol^−1^, in which the OH is directly rebounded to CH_3_· before it is connecting on the corrolazine ring, thus greatly improving the selectivity of the reaction.

## Data Availability

The original contributions presented in the study are included in the article/Supplementary Material, further inquiries can be directed to the corresponding authors.
