# Supplementary material for: Oriented External Electric Fields Regurating the Reaction Mechanism of CH4 Oxidation Catalyzed by Fe(IV)-Oxo-Corrolazine: Insight from Density Functional Calculations
Source: Front Chem. 2022 Jun 29;10:896944. doi: 10.3389/fchem.2022.896944 (PMC9277104; doi:10.3389/fchem.2022.896944)
Supplement: Supplementary file 1 [file DataSheet1.docx]

Supplementary Material

# Supplementary Tables

**Table S1.** The relative energies (ΔG, kcal·mol^-1^) and absolute energies (G, kcal·mol^-1^) of Fe(IV)-Oxo-Cz, in doublet, quartet and sextet states.

| Energies | doublet | quartet | sextet |
| --- | --- | --- | --- |
| absolute energies (G) | -750751.0739 | -750761.3199 | -750721.2685 |
| relative energies (ΔG) | 10.25 | 0.00 | 40.05 |

**Table S2.** The spin densities of Fe(IV)-Oxo-Cz, in doublet, quartet and sextet states.

| Atoms | spin densities | | |
| --- | --- | --- | --- |
|  | doublet | quartet | sextet |
| Fe | 0.905 | 1.259 | 1.253 |
| O | 0.107 | 0.719 | 0.727 |

**Table S3.** The NPA charges (|e|) of Fe(IV)-Oxo-Cz, in doublet, quartet and sextet states.

| Atoms | NPA charges(\|e\|) | | |
| --- | --- | --- | --- |
|  | doublet | quartet | sextet |
| Fe | 0.533 | 0.536 | 0.545 |
| O | -0.162 | -0.331 | -0.326 |

**Table S4.** Selected Bond Lengths (Å) and the relative electronic energies (ΔG, kcal·mol^-1^) of species involved in the reaction of CH_4_ oxidation catalyzed for the doublet of Fe(IV)-Oxo-Cz.

| Complexes | d_Fe_**_‒_**_O_ | d_O_**_‒_**_H_ | d_C_**_‒_**_H_ | d_C_**_‒_**_O_ | ΔG |
| --- | --- | --- | --- | --- | --- |
| RC | 1.614 | 2.389 | 1.093 | 3.477 | 0.00 |
| TS1 | 1.709 | 1.197 | 1.319 | 2.515 | 20.72 |
| INT | 1.770 | 0.985 | 2.057 | 3.041 | 15.61 |
| TS2 | 1.790 | 0.972 | 2.731 | 2.420 | 21.87 |
| P | 2.110 | 0.968 | 1.999 | 1.447 | -25.43 |

**Table S5.** The effect on the dipole moments (*μ*_z_), the spin density (ρ_spin_) of Fe**‒**O for the RC, TS1, and the energy barrier (ΔG, kcal·mol^-1^) of CH_4_ oxidation catalyzed by Fe(IV)-Oxo-Cz at doublet state under selected external electric field intensities of *F*_z1_.

| *F*_z1_ (×10^-4^ a.u.) | RC | | | TS1 | | | Energy Barrier  (kcal·mol^-1^) |
| --- | --- | --- | --- | --- | --- | --- | --- |
|  | ΔG | *μ*_z_ | ρ_spin_ (Fe**‒**O) | ΔG | *μ*_z_ | ρ_spin_ (Fe**‒**O) |  |
| -100 | 5.94 | 3.69 | 1.044 | 6.19 | 8.57 | 0.908 | 20.97 |
| -40 | 3.14 | -2.22 | 1.951 | -1.21 | 2.23 | 1.543 | 16.38 |
| -20 | 1.45 | -2.67 | 1.950 | -0.26 | 1.13 | 1.562 | 19.00 |
| 0 | 0.00 | -3.91 | 1.953 | 0.00 | 0.12 | 1.585 | 20.72 |
| 20 | -2.24 | -4.87 | 1.952 | -0.19 | -1.08 | 1.615 | 22.77 |
| 40 | -4.92 | -5.99 | 1.953 | -1.13 | -2.22 | 1.655 | 24.50 |
| 100 | -17.03 | -9.80 | 1.950 | -14.29 | -9.33 | 2.396 | 23.46 |

**Table S6.** The effect on the dipole moments (*μ*_z_) of the RC, TS1, and the energy barrier (ΔG, kcal·mol^-1^) of CH_4_ oxidation catalyzed by Fe(IV)-Oxo-Cz at doublet state under selected external electric field intensities of *F*_z2_.

| *F*_z2_ (×10^-4^ a.u.) | RC | | | TS1 | | | Energy barrier  (kcal·mol^-1^) |
| --- | --- | --- | --- | --- | --- | --- | --- |
|  | ΔG | *μ*_z_ | ρ_spin_ (Fe**‒**O) | ΔG | *μ*_z_ | ρ_spin_ (Fe**‒**O) |  |
| -100 | -9.03 | 9.02 | 1.955 | -2.90 | 12.04 | 0.983 | 26.81 |
| -40 | -0.02 | 2.27 | 1.955 | -2.79 | 5.16 | 1.556 | 17.90 |
| -20 | 0.64 | 0.10 | 1.954 | -0.83 | 2.98 | 1.570 | 19.21 |
| 0 | 0.00 | -2.06 | 1.953 | 0.00 | -1.31 | 1.585 | 20.72 |
| 20 | -2.06 | -4.36 | 1.951 | -0.15 | -1.43 | 1.604 | 22.59 |
| 40 | -4.24 | -6.46 | 1.950 | -1.86 | -3.69 | 1.630 | 23.05 |
| 100 | -18.59 | -12.66 | 1.946 | -17.69 | -11.86 | 2.319 | 21.58 |

As shown in Tables S5 and S6, for *F*_z_ > 0, the dipole moment of the RC increases. Therefore, the RC is stabilized by the applied OEEFs originating from the attractive interaction between the increased dipole moments and the OEEFs. While for -0.004 a.u. < *F*_z1_ < 0, the OEEF decreases the dipole moment of in z1 direction of the RC, and the repulsion between *F*_z1_ and the dipole moment in z1 direction of the RC destabilizes the RC. However, when *F*_z1_ is further increased to *F*_z1_ = -0.010 a.u., it flips the orientation of the dipole moments of the RC, but the RC is still destabilized by the OEEF, which is due to the change in the electronic configuration. For -0.004 a.u < *F*_z1_ < 0., the Fe**‒**O moiety has a spin density greater than 1.950, while the corrolazine ring moiety has approximately one electron with opposite spin, thus forming a weak-field high-spin electronic configuration. However, for *F*_z1_ = -0.010 a.u., the Fe**‒**O moiety has a spin electron density of 1.044, thus forming a strong-field low-spin electron configuration. For *F*_z2_ < 0, the RC are initially destabilized by the electric field and then stabilized by the electric field, originating from the repulsive and attractive effects of the electric field and dipole moment, respectively.

As shown in Tables S5 and S6, similar to RC, the TS1 is stabilized by the OEEFs for -0.004 a.u. < *F*_z1_ < 0.01 a.u. and -0.010 a.u. < *F*_z2_ < 0.010 a.u the TS1 is stabilized by the OEEFs originating from the attractive interaction between the increased dipole moments and the OEEFs. However, for *F*_z1_ = -0.010 a.u. the TS1 is destabilized by the applied OEEFs due to its electron configuration change.

**Table S7.** The effect on the reaction of CH_4_ oxidation catalyzed by Fe(IV)-Oxo-Cz at quartet states under the external electric field intensities of *F*_z1_ = 0.00, -0.005 and -0.010 a.u..

| Multiplicity | *F*_z1_ (×10^-4^ a.u.) | Energy barrier (kcal·mol^-1^) | | | | |
| --- | --- | --- | --- | --- | --- | --- |
|  |  | RC | TS1 | INT | TS2 | P |
| Quartet state | 0 | 0.00 | 25.44 | 15.64 | 17.63 | -40.93 |
|  | -50 | 0.00 | 20.83 | 11.44 | 12.46 | -50.44 |
|  | -100 | 0.00 | 17.34 | --- | --- | -58.53 |

As shown in Table S7, for *F*_z1_ = -0.005 a.u., the relative energy of TS1 is 20.83 kcal·mol^-1^, while the relative energy of TS2 is 12.46 kcal·mol^-1^, so TS1 remains the rate-determining step.

# Supplementary Figure

#
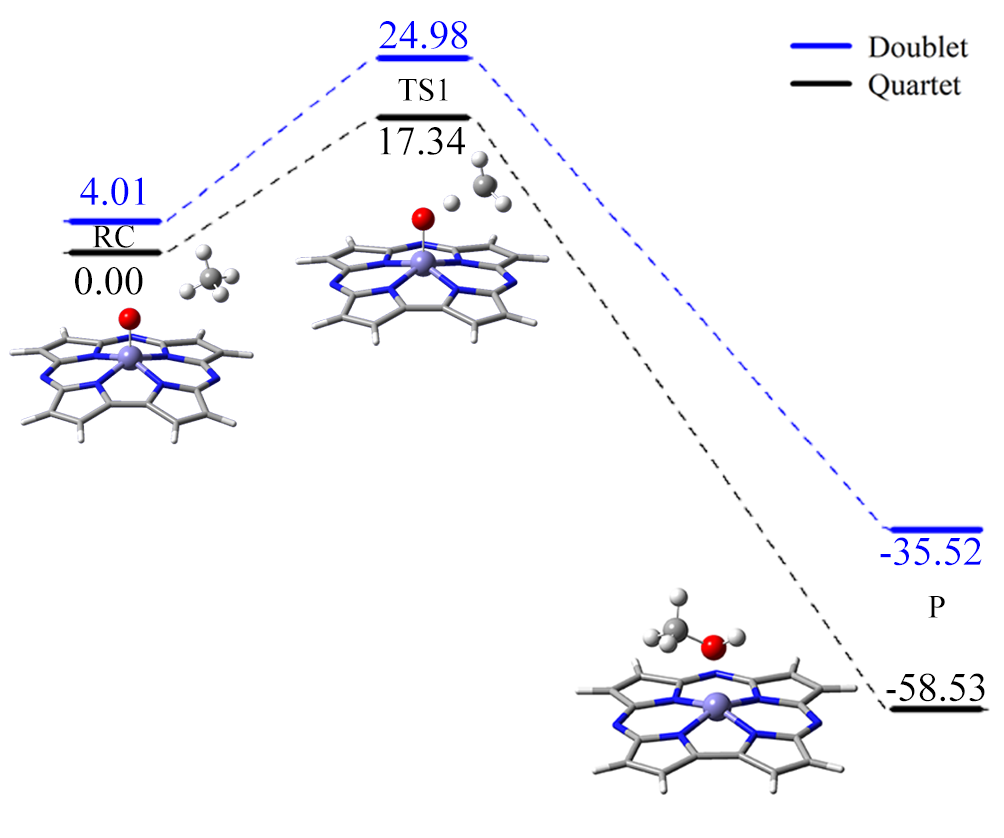


**Figure S1**. The predicted reaction pathway of CH_4_ oxidation catalyzed by the Fe(IV)-Oxo-Cz at doublet and quartet states under the electric field of *F*_z1_ = -0.010 a.u. The presented relative free energy values (ΔG, kcal·mol^-1^).

As shown in Table S7 and Figure S1, for *F*_z1_ =0.010 a.u., TS2 disappeared and the two-step reaction process became a one-step with OEEF.

# Coordinates

All geometry optimizations were performed at the B3LYP-D3(BJ)/6-31G++(d,p), LANL2TZ(Fe) level of theory with Gaussian 16.

**Coordinates in the doublet state (RC^2^, TS1^2^, INT^2^, TS2^2^, P^2^)**

**RC^2^**

Fe -0.11542900 0.02250900 0.39068200

H -0.58349800 5.01652800 -0.56445800

H -0.84279400 -4.86067700 -0.71085000

H -3.12186800 4.13342500 -0.20040600

H 1.79179600 -4.50669600 -1.10760200

H 4.01729300 2.57576100 -1.19376700

H -3.92345900 -3.23079000 -0.31363600

H -5.14308900 -0.83821200 -0.20951500

H 4.74527400 -0.03652200 -1.25274100

N -0.80934500 1.73271000 -0.07828500

N 0.25740700 -1.79335100 -0.03887600

N 1.56788900 0.47632400 -0.37747300

N -1.88289200 -0.66097300 0.23275600

C -0.92996100 4.00964000 -0.37892100

C -0.27435500 -3.95466700 -0.55264400

C -2.20099200 3.56732600 -0.19252500

C 1.07449200 -3.77635200 -0.76007400

C -0.04431100 2.84119500 -0.32178000

C -0.81276500 -2.68295100 -0.12296700

C -2.14800800 2.10954700 -0.02099800

C 1.38782100 -2.37950100 -0.46963400

N 1.27535300 2.87334200 -0.53212800

N -3.20667100 1.32143100 0.06684000

N 2.55697600 -1.72906100 -0.68711700

C 2.00597900 1.75570700 -0.59130600

C -2.04498200 -2.03090500 0.03364100

C -3.06039100 -0.02610000 0.10455400

C 2.60904700 -0.40845700 -0.64339900

C 3.42330700 1.70178400 -0.96668500

C -3.46061600 -2.26875600 -0.14251500

C -4.08831600 -1.04475000 -0.09341200

C 3.78828400 0.39383800 -0.99330300

O 0.13317500 0.13558500 1.98099200

C 3.53289800 -0.23040700 2.61184600

H 4.06821300 0.58924600 2.12471500

H 2.45578600 -0.08824500 2.49541500

H 3.83007500 -1.17737700 2.15316300

H 3.78822400 -0.24643700 3.67471200

**TS1^2^**

Fe 0.08466300 -0.01797800 0.34114600

H -0.03745100 -5.02972800 -0.58044300

H 1.28720900 4.79860600 -0.56645000

H 2.59383800 -4.42915900 -0.29633600

H -1.38746200 4.76161100 -0.83601800

H -4.36046600 -2.08080000 -0.99778800

H 4.22903300 2.78426100 -0.27241100

H 5.17996900 0.27101900 -0.17180700

H -4.78536000 0.59950700 -1.03056600

N 0.57935700 -1.79137000 -0.14186500

N -0.07871600 1.81602100 -0.13147900

N -1.64330800 -0.26493200 -0.38880900

N 1.89450100 0.46142500 0.07756500

C 0.41646300 -4.06278800 -0.41520800

C 0.63537500 3.94297000 -0.45533100

C 1.74594000 -3.75920300 -0.26850900

C -0.74240200 3.92727200 -0.59830100

C -0.32576800 -2.82668900 -0.35002100

C 1.05625600 2.59891700 -0.17463100

C 1.86352700 -2.32695800 -0.10719400

C -1.19342300 2.56637500 -0.42106000

N -1.64085100 -2.69452700 -0.51502900

N 3.01533800 -1.65234700 -0.03196900

N -2.41238700 2.05278900 -0.58906900

C -2.25114400 -1.51040200 -0.55206500

C 2.22580200 1.79957400 -0.05594000

C 3.02842800 -0.31936500 0.01181700

C -2.60745300 0.72834200 -0.57197400

C -3.64853400 -1.28732900 -0.81875200

C 3.65269300 1.87719200 -0.15209800

C 4.14703600 0.58214400 -0.10216800

C -3.86428000 0.07011400 -0.83044000

O -0.23880600 -0.08291200 2.01831600

C -2.64020200 0.20335100 2.70944300

H -3.15691300 -0.60468600 2.19584200

H -1.38032500 0.05006300 2.35143600

H -2.88928000 1.20200800 2.35651600

H -2.57100600 0.09751700 3.79085400

**INT^2^**

Fe -0.10120800 0.01561300 0.33889100

H -0.15610700 5.03335200 -0.55919300

H -1.15101100 -4.83618900 -0.55139000

H -2.76172300 4.34380300 -0.24340100

H 1.51633800 -4.71008800 -0.85665000

H 4.25596200 2.23097900 -1.07334900

H -4.16147000 -2.92117100 -0.22092700

H -5.19631500 -0.44294600 -0.09988200

H 4.76887900 -0.43353300 -1.12121500

N -0.66223100 1.77221400 -0.13986900

N 0.11407900 -1.80475000 -0.15483400

N 1.61724000 0.32454900 -0.39974600

N -1.90129100 -0.52362300 0.10060700

C -0.57561600 4.05053500 -0.39695100

C -0.52755000 -3.95791400 -0.45516600

C -1.89197200 3.70196100 -0.23391800

C 0.84639400 -3.89640500 -0.61628200

C 0.20739700 2.83863700 -0.35035800

C -0.99059800 -2.62621300 -0.17558500

C -1.95870900 2.26488300 -0.08110600

C 1.25336100 -2.51965800 -0.45067800

N 1.52349300 2.75011000 -0.53156300

N -3.08942200 1.55106200 0.00829100

N 2.45076800 -1.96561000 -0.63099500

C 2.17781400 1.58856200 -0.57633200

C -2.18783400 -1.86672700 -0.03249600

C -3.06103200 0.22026700 0.05257600

C 2.60699900 -0.63353300 -0.60990600

C 3.57539800 1.41431800 -0.87751100

C -3.61531300 -1.99481200 -0.10718300

C -4.15245800 -0.71884000 -0.04700300

C 3.83567300 0.06452400 -0.89833300

O 0.22600100 0.08994400 2.07635600

C 3.18869800 -0.18591100 2.70219200

H 3.44998800 0.66939900 2.09075100

H 1.17612200 -0.00530800 2.31945500

H 3.27858900 -1.18170700 2.28462300

H 3.05175500 -0.06181100 3.77017700

**TS2^2^**

Fe -0.07894600 0.00939000 0.31628200

H 2.52607400 4.22818600 -0.82762600

H -3.53396900 -3.60150100 -0.33888500

H -0.04635700 5.03987600 -0.54902700

H -1.20231600 -4.91389400 -0.58673800

H 4.80150500 -0.50836800 -1.05231100

H -5.08032500 -0.37598600 -0.21076400

H -4.65057800 2.27614700 -0.26118600

H 3.82883600 -3.03810300 -0.93553700

N 0.37432700 1.75777000 -0.25580400

N -0.85548500 -1.67750300 -0.03890700

N 1.55080400 -0.68523700 -0.38025700

N -1.89944000 0.48866700 0.10177000

C 1.65213100 3.62510400 -0.62499700

C -2.54154700 -3.17796600 -0.26669300

C 0.34964600 4.03632800 -0.47959900

C -1.34019000 -3.85843700 -0.39767000

C 1.67904900 2.18926600 -0.49718100

C -2.23467800 -1.79339000 -0.05423700

C -0.46972500 2.86750400 -0.25648800

C -0.26828200 -2.89966800 -0.28242100

N 2.75044900 1.40865000 -0.63559200

N -1.80640800 2.86545500 -0.16293100

N 1.04270700 -3.07447300 -0.45700700

C 2.69170400 0.07574800 -0.60351600

C -2.85109700 -0.51190200 0.01952500

C -2.48832300 1.72474700 -0.04364700

C 1.88220000 -2.03122200 -0.49911800

C 3.79110600 -0.82865000 -0.84041400

C -4.12848300 0.12812900 -0.11385700

C -3.91057400 1.49679900 -0.14401500

C 3.29917900 -2.10903500 -0.77752800

O 0.34515200 0.05884500 2.05470400

C 2.61862600 -0.20886100 2.83805600

H 2.99892500 0.69202400 2.37520800

H 0.15156100 0.90905800 2.48510400

H 2.67491900 -1.14504000 2.30119900

H 2.33998200 -0.20756700 3.88351700

**P^2^**

Fe -0.07163600 -0.01336300 0.11357300

H -4.56683700 -2.31051700 -0.24511300

H 4.62310600 1.42011800 -0.79966800

H -2.74011000 -4.31537100 -0.27749700

H 3.26168900 3.73892700 -0.72197800

H -4.16807600 2.93071000 -0.33754600

H 4.34250600 -2.16236200 -0.78706100

H 2.64389800 -4.24441900 -0.64945100

H -2.05917800 4.63421500 -0.41822200

N -1.41797800 -1.27165300 -0.12110900

N 1.37756800 1.08287600 -0.27245100

N -1.21557000 1.43323500 -0.13369700

N 1.19838200 -1.32751200 -0.19291700

C -3.48899500 -2.23599200 -0.20721200

C 3.56183100 1.54215400 -0.63143000

C -2.56477200 -3.25008200 -0.22037500

C 2.85998900 2.74314700 -0.59405900

C -2.76858300 -0.98333200 -0.15595200

C 2.61320500 0.48767400 -0.43419900

C -1.24611500 -2.65574800 -0.17950500

C 1.46602000 2.43703300 -0.38186100

N -3.32111200 0.23102900 -0.16529600

N -0.09945700 -3.32873400 -0.25256800

N 0.40390000 3.25988200 -0.33962800

C -2.59392000 1.35076100 -0.18196500

C 2.50751400 -0.92991200 -0.40015300

C 1.07613700 -2.67975400 -0.29868900

C -0.83382400 2.77417400 -0.24652400

C -3.11454400 2.69460400 -0.28155900

C 3.27925100 -2.11851000 -0.59459000

C 2.40237500 -3.19763500 -0.52645500

C -2.04621300 3.55770400 -0.31864500

O 0.16394600 0.14949600 2.20397900

C 1.48877400 0.09140200 2.78221400

H 2.10372200 0.91687700 2.41547700

H -0.28301300 0.96459900 2.47472100

H 1.91649500 -0.85622400 2.45932600

H 1.40786600 0.11494300 3.87169100

**Coordinates in the quartet state(RC^4^, TS1^4^, INT^4^, TS2^4^, P^4^)**

**RC^4^**

Fe 0.11508300 -0.02256300 0.38503700

H 0.46409300 -5.02631900 -0.57236600

H 0.94986100 4.84967100 -0.68887600

H 3.02231600 -4.20352000 -0.20921400

H -1.69317400 4.55961400 -1.07746700

H -4.07886900 -2.47552700 -1.20033500

H 4.00159800 3.14152000 -0.29783800

H 5.16466400 0.72153900 -0.19414900

H -4.74457500 0.15297300 -1.25499000

N 0.76664800 -1.74969100 -0.08073900

N -0.21486800 1.80099600 -0.04745200

N -1.58209500 -0.43660900 -0.37437000

N 1.89786500 0.61861500 0.21900500

C 0.83376300 -4.02824800 -0.38401200

C 0.36238400 3.95444400 -0.53894600

C 2.11518700 -3.61564200 -0.20080800

C -0.99179700 3.80973200 -0.73919600

C -0.02432500 -2.83914000 -0.32537700

C 0.87445200 2.66622000 -0.12517800

C 2.09679100 -2.15707200 -0.02618100

C -1.33500300 2.41727200 -0.46205600

N -1.34437300 -2.83916000 -0.53778700

N 3.17425000 -1.39431200 0.06287400

N -2.51870400 1.79332400 -0.68040500

C -2.04958500 -1.70478100 -0.59212000

C 2.09211800 1.98457500 0.02861600

C 3.06126900 -0.04341300 0.10255200

C -2.60093200 0.47371800 -0.64143700

C -3.46504400 -1.61658500 -0.96871600

C 3.51475400 2.18982100 -0.13566300

C 4.11418900 0.95162400 -0.08359100

C -3.79803700 -0.30016200 -0.99595900

O -0.11749600 -0.10971200 1.98092000

C -3.51792400 0.23363700 2.62387600

H -4.03734900 -0.58773900 2.12271300

H -2.43789600 0.10113000 2.52508100

H -3.81432600 1.18060000 2.16455400

H -3.79314900 0.24224000 3.68183400

**TS1^4^**

Fe 0.07883000 -0.02201400 0.32328000

H 0.82530500 -4.99663100 -0.50008900

H 0.43747900 4.91125100 -0.68054100

H 3.31014900 -3.94740900 -0.23432800

H -2.18313200 4.39523400 -0.96800600

H -3.95230300 -2.84514400 -0.97607000

H 3.67519500 3.45239500 -0.38226400

H 5.04106400 1.14106200 -0.28063300

H -4.83805100 -0.28493300 -1.05525000

N 0.86667300 -1.69351800 -0.11882800

N -0.40452600 1.74573300 -0.14858500

N -1.59731400 -0.59021500 -0.34470500

N 1.79122400 0.76319300 0.11166700

C 1.10556700 -3.96276200 -0.35481000

C -0.05383700 3.95760500 -0.54520100

C 2.35916100 -3.43380200 -0.21449900

C -1.40560400 3.69428900 -0.69764500

C 0.15411600 -2.86789100 -0.30663900

C 0.59218500 2.72117200 -0.21275000

C 2.22786700 -1.99364300 -0.07886400

C -1.61553000 2.28084600 -0.47256000

N -1.16396000 -2.97346600 -0.46167300

N 3.24324400 -1.13094400 -0.03252300

N -2.73984200 1.55717500 -0.62526600

C -1.97403600 -1.90940400 -0.50671400

C 1.87583200 2.14451300 -0.07033700

C 3.02254900 0.19212800 -0.00483900

C -2.71730500 0.22990300 -0.57115900

C -3.39345100 -1.93715100 -0.79717000

C 3.26758800 2.46375500 -0.22254500

C 3.97302800 1.27463600 -0.17819700

C -3.84289800 -0.64311600 -0.83211800

O -0.14190600 -0.11132600 2.03184800

C -2.52118300 0.27191600 2.83532700

H -3.04631800 -0.57109400 2.38896300

H -1.24831100 0.07555700 2.41857400

H -2.77916200 1.24104300 2.41201500

H -2.44901000 0.24795700 3.92127600

**INT^4^**

Fe -0.10347700 0.01426100 0.33769600

H -0.76946700 4.99882800 -0.49612300

H -0.55109200 -4.91630300 -0.61092200

H -3.27150600 3.99201300 -0.19334000

H 2.08007400 -4.46060200 -0.91710200

H 3.95247800 2.76548000 -1.03784000

H -3.77335800 -3.39036600 -0.25350600

H -5.10439900 -1.05935900 -0.10645000

H 4.78647800 0.18421000 -1.12729500

N -0.87440100 1.69476100 -0.12243500

N 0.33440800 -1.75819300 -0.17406000

N 1.56638200 0.54110300 -0.39730200

N -1.82363100 -0.73740600 0.08714400

C -1.06595900 3.97001600 -0.34832700

C -0.03966200 -3.96962800 -0.50338900

C -2.32984400 3.46118700 -0.19165100

C 1.31635200 -3.73826000 -0.66482400

C -0.14105000 2.86204400 -0.31669000

C -0.66138800 -2.70871000 -0.20666500

C -2.22009500 2.02476900 -0.05806100

C 1.55222900 -2.32478300 -0.48180900

N 1.17597700 2.93739300 -0.49554900

N -3.25608100 1.17679800 0.02112300

N 2.67168900 -1.62570200 -0.65947600

C 1.96823900 1.86566300 -0.55416000

C -1.94317400 -2.10257900 -0.05716600

C -3.06557900 -0.14027800 0.04953900

C 2.66458400 -0.28504200 -0.62240000

C 3.37703900 1.86878200 -0.85560200

C -3.34511400 -2.40492900 -0.13165100

C -4.03441700 -1.20563700 -0.05868300

C 3.79967600 0.56157900 -0.89832700

O 0.17020900 0.11381900 2.09402900

C 3.23812000 -0.15718800 2.71358200

H 3.44163700 0.74755500 2.15343300

H 1.11120600 0.02431700 2.35088400

H 3.30780900 -1.11691700 2.21583200

H 3.13873500 -0.11349200 3.79170400

**TS2^4^**

Fe -0.09124600 0.01454900 0.32230400

H 0.37353000 4.99418300 -0.61573400

H -1.59807200 -4.71817900 -0.52250600

H -2.28964000 4.57434900 -0.32070100

H 1.07112300 -4.86036500 -0.79067200

H 4.50094000 1.76355300 -1.03872100

H -4.41510400 -2.51560600 -0.21363800

H -5.20280500 0.05145000 -0.13122000

H 4.74126600 -0.93800500 -1.04660000

N -0.45824200 1.81225600 -0.16207200

N -0.04516500 -1.82880800 -0.11386800

N 1.67228000 0.13778900 -0.40002700

N -1.93343100 -0.34918900 0.04199700

C -0.14265000 4.05937100 -0.44651700

C -0.89080500 -3.90587900 -0.42230200

C -1.49008200 3.84701800 -0.29320500

C 0.48260800 -3.98240000 -0.56263800

C 0.51501800 2.77861400 -0.37905000

C -1.22240200 -2.53229400 -0.15722100

C -1.70440600 2.42962300 -0.12714300

C 1.02329300 -2.65161400 -0.39983400

N 1.82119700 2.55618800 -0.54766000

N -2.90219000 1.83240000 -0.04823300

N 2.26831300 -2.22238100 -0.57933400

C 2.35559000 1.33807900 -0.57674200

C -2.34514400 -1.65355400 -0.05354900

C -3.00952500 0.50665200 0.00009000

C 2.55617000 -0.90967200 -0.57791800

C 3.73769900 1.02165600 -0.84905500

C -3.78095900 -1.64416200 -0.12139400

C -4.18976700 -0.32337300 -0.08174700

C 3.85933700 -0.34404400 -0.85035300

O 0.14097800 0.12059900 2.11526900

C 2.91424500 -0.27694300 2.78176800

H 3.36330800 0.42436700 2.09035900

H 0.34748800 1.03092100 2.39762400

H 2.56457200 -1.23468100 2.42442200

H 2.81930600 -0.02532100 3.83058400

**P^4^**

Fe -0.06510100 0.00659000 0.10696800

H -0.03233500 5.03999200 -0.48807700

H -1.05281100 -4.82964100 -0.54990300

H -2.65493900 4.36351400 -0.34094300

H 1.62775400 -4.71145100 -0.73463200

H 4.40926000 2.23484300 -0.71114900

H -4.06599700 -2.92538300 -0.34241200

H -5.11161500 -0.44784500 -0.29810500

H 4.92678600 -0.42763100 -0.76010700

N -0.57039800 1.78617000 -0.14818000

N 0.21560400 -1.80444800 -0.13927500

N 1.72293400 0.32949300 -0.33155200

N -1.83695100 -0.50430600 -0.03385500

C -0.46412500 4.05597400 -0.36887900

C -0.43614000 -3.94777800 -0.44282000

C -1.79248800 3.71329900 -0.29336600

C 0.95239300 -3.88982600 -0.53945800

C 0.31111400 2.83820900 -0.29781300

C -0.90450400 -2.61874700 -0.20804100

C -1.87870000 2.27533100 -0.17129500

C 1.35328400 -2.51592800 -0.37755200

N 1.64106400 2.75370500 -0.39337000

N -3.00998600 1.57153500 -0.14383800

N 2.57307500 -1.96089800 -0.48519200

C 2.28740300 1.58493000 -0.42784000

C -2.10301800 -1.85865500 -0.13924300

C -2.98180400 0.22830800 -0.11475100

C 2.72098900 -0.63596500 -0.46496700

C 3.71271300 1.41524200 -0.60136600

C -3.52235200 -1.99574700 -0.24430100

C -4.06646700 -0.71469000 -0.22310100

C 3.97487900 0.06688100 -0.62481900

O 0.20276700 0.14255000 2.27234400

C 1.43659100 -0.38121800 2.81190800

H 2.29284000 0.19476900 2.45105000

H 0.07858000 1.06087700 2.55057100

H 1.51242400 -1.40688500 2.45260300

H 1.39454200 -0.37338300 3.90425200

**Coordinates of the quartet state under the *F*_z1_**

**RC (*F*_z1_=+0.001 a.u.)**

Fe -0.00640500 0.27644000 0.35420800

H 2.39566300 -4.22864400 0.16419900

H -1.40092700 4.71310100 -1.63444400

H 4.37132900 -2.37563300 0.03051000

H -3.68422600 3.29789200 -1.57977900

H -2.82463300 -3.94917800 -0.22659200

H 2.10116400 4.52251400 -1.40927500

H 4.19297000 2.87107000 -1.06830500

H -4.55676400 -1.89647900 -0.60195900

N 1.29396500 -1.08973700 0.09186000

N -1.10908400 1.66966800 -0.32442100

N -1.40209800 -0.94478800 -0.08003800

N 1.31753800 1.53862900 -0.16650600

C 2.31295200 -3.15112100 0.14424800

C -1.53965900 3.71255900 -1.24885000

C 3.30232400 -2.22238200 0.07846600

C -2.71124700 2.99068300 -1.22260500

C 1.03352800 -2.43279800 0.13443400

C -0.50205800 2.86513800 -0.70268800

C 2.67100900 -0.89731500 0.02069200

C -2.40813500 1.67365300 -0.66862600

N -0.16814200 -3.01728000 0.10825100

N 3.32149400 0.23890000 -0.16935800

N -3.22204700 0.59131700 -0.60444500

C -1.29262600 -2.30825000 -0.02926500

C 0.89633500 2.78936900 -0.61232800

C 2.64302900 1.40057700 -0.33889200

C -2.72750900 -0.60738600 -0.34248300

C -2.62772900 -2.88654100 -0.21695600

C 2.08063500 3.51596000 -1.01500400

C 3.15468900 2.67264000 -0.84195200

C -3.49513500 -1.85820100 -0.40206300

O -0.09355600 0.44168100 1.95940800

C -2.13009600 -2.03070400 3.28523300

H -1.67760300 -2.98275800 2.99435000

H -1.50620000 -1.20566000 2.93300800

H -3.12637900 -1.95574100 2.84129800

H -2.21501600 -1.98832500 4.37454500

**TS1 (*F*_z1_=+0.001 a.u.)**

Fe -0.12328900 -0.01841100 0.45340600

H 2.51390400 -4.34103600 -0.14060500

H -1.84923100 4.52212900 -0.92136700

H 4.37440600 -2.37077000 -0.18793600

H -4.05051900 2.98271700 -0.91592900

H -2.74212500 -4.32196800 -0.27970000

H 1.70524400 4.50510900 -0.85413300

H 3.89210300 2.94894600 -0.74003500

H -4.59030300 -2.34574000 -0.43510200

N 1.24246000 -1.27325600 0.04159200

N -1.30915700 1.35653300 -0.08131700

N -1.46545700 -1.26782200 -0.01164900

N 1.10953200 1.35881400 0.02854100

C 2.36214300 -3.27240200 -0.08129500

C -1.90551100 3.47259900 -0.66754200

C 3.30103400 -2.27849400 -0.09892500

C -3.04212900 2.68092400 -0.66893200

C 1.05458900 -2.64605800 -0.00358400

C -0.79678700 2.63513900 -0.31144600

C 2.60783300 -1.00285300 -0.03711900

C -2.64912600 1.33163500 -0.32461600

N -0.11474600 -3.28144000 -0.02063100

N 3.18895100 0.19162900 -0.14664600

N -3.39323800 0.20958800 -0.31961000

C -1.28678200 -2.63591300 -0.05423400

C 0.61558100 2.63307700 -0.25815500

C 2.45405400 1.31383200 -0.18163500

C -2.83329900 -0.98642900 -0.18273500

C -2.58907300 -3.25421700 -0.20731500

C 1.74519500 3.46007900 -0.57962500

C 2.87178000 2.65992900 -0.52921500

C -3.52425200 -2.25598000 -0.28066600

O -0.19032900 -0.02849100 2.17636800

C -2.46489800 -0.58359800 3.17413100

H -2.62168100 -1.60036000 2.81754300

H -1.23861400 -0.27600700 2.64711500

H -3.12155000 0.15973800 2.72561800

H -2.32471400 -0.48717100 4.24938700

**RC (*F*_z1_=+0.002 a.u.)**

Fe -0.01662500 0.28560700 0.36336100

H 2.37608600 -4.22543400 0.16895300

H -1.38954700 4.71138500 -1.66366900

H 4.35633100 -2.37641900 0.04164700

H -3.67646300 3.30208400 -1.61270200

H -2.83747100 -3.93631600 -0.23971800

H 2.10295300 4.51492900 -1.42491000

H 4.19075900 2.86131200 -1.07046500

H -4.56470500 -1.88059100 -0.62287100

N 1.28183800 -1.08353500 0.10583600

N -1.11424600 1.67915200 -0.32263900

N -1.41243900 -0.93364700 -0.07672000

N 1.31209800 1.54356900 -0.15540300

C 2.29624100 -3.14759000 0.15574800

C -1.53423000 3.71592400 -1.26729200

C 3.28779800 -2.22098600 0.09310200

C -2.70747200 2.99722800 -1.24291000

C 1.01877200 -2.42630200 0.14565000

C -0.50252500 2.87027700 -0.70718400

C 2.65942600 -0.89464100 0.03685000

C -2.41085800 1.68394500 -0.67668700

N -0.18355300 -3.00878300 0.11612400

N 3.31333000 0.23958200 -0.15223600

N -3.22754000 0.60416200 -0.61254100

C -1.30569600 -2.29750300 -0.02683100

C 0.89469300 2.79200700 -0.61138000

C 2.63743600 1.40168000 -0.32722200

C -2.73594700 -0.59471000 -0.34674400

C -2.64019100 -2.87383700 -0.22298400

C 2.08061500 3.51297800 -1.01919800

C 3.15248900 2.66864200 -0.83917100

C -3.50512900 -1.84412900 -0.41200400

O -0.10964200 0.45160600 1.96900600

C -2.08411100 -2.06581200 3.29292900

H -1.60538000 -3.00519900 3.00308700

H -1.48046800 -1.22465000 2.94327900

H -3.07960800 -2.01683800 2.84368300

H -2.17566800 -2.02628200 4.38210300

**TS1 (*F*_z1_=+0.002 a.u.)**

Fe -0.12215000 -0.01136800 0.46521800

H 2.50970500 -4.33622100 -0.14635300

H -1.83936900 4.52398300 -0.94152200

H 4.37201300 -2.36782400 -0.19586000

H -4.04229200 2.98725200 -0.93270100

H -2.74274800 -4.31226600 -0.28351000

H 1.70636400 4.50227900 -0.88045600

H 3.89183000 2.94467100 -0.76513800

H -4.58949900 -2.33486700 -0.44049100

N 1.24222900 -1.26717600 0.05229200

N -1.30692900 1.36401700 -0.07229400

N -1.46499400 -1.25987700 -0.00053600

N 1.11323900 1.36570400 0.04251600

C 2.35992800 -3.26778600 -0.07914100

C -1.89859900 3.47739800 -0.67654000

C 3.29943400 -2.27519300 -0.09798700

C -3.03577000 2.68729300 -0.67608800

C 1.05289300 -2.63961800 0.00461400

C -0.79222800 2.64077800 -0.30998100

C 2.60721300 -0.99842300 -0.03087300

C -2.64488900 1.33980400 -0.32085900

N -0.11632600 -3.27426500 -0.01095600

N 3.18973800 0.19479600 -0.14234400

N -3.39094400 0.21824600 -0.31427500

C -1.28780700 -2.62683700 -0.04532900

C 0.61902300 2.63788100 -0.25549000

C 2.45517600 1.31752900 -0.17817500

C -2.83248000 -0.97723500 -0.17589100

C -2.59032400 -3.24494100 -0.20405000

C 1.74770700 3.46118000 -0.59165200

C 2.87304700 2.66040700 -0.54082600

C -3.52449600 -2.24656800 -0.27829800

O -0.20075800 -0.02706300 2.18688900

C -2.47479800 -0.62130200 3.17903200

H -2.62142400 -1.63351500 2.80579700

H -1.23598800 -0.29109500 2.64874500

H -3.13451900 0.12404600 2.73847500

H -2.33820700 -0.53982300 4.25603800

**RC (*F*_z1_=+0.003 a.u.)**

Fe -0.02600400 0.29351100 0.37238600

H 2.35861500 -4.22258500 0.17098000

H -1.37907800 4.70876000 -1.69037400

H 4.34271800 -2.37713200 0.04994700

H -3.66917600 3.30494400 -1.64249000

H -2.84855600 -3.92503400 -0.25337000

H 2.10409000 4.50722200 -1.43948400

H 4.18831500 2.85208600 -1.07247100

H -4.57131200 -1.86672500 -0.64304200

N 1.27080900 -1.07804200 0.11904200

N -1.11906400 1.68750200 -0.32002200

N -1.42169400 -0.92385800 -0.07331000

N 1.30698600 1.54800800 -0.14416900

C 2.28119100 -3.14449600 0.16501400

C -1.52928600 3.71827300 -1.28373300

C 3.27467100 -2.21972400 0.10545900

C -2.70400800 3.00248800 -1.26092600

C 1.00552100 -2.42061800 0.15559100

C -0.50307900 2.87454300 -0.71060900

C 2.64889800 -0.89227000 0.05163800

C -2.41336000 1.69288300 -0.68323800

N -0.19733600 -3.00139300 0.12304300

N 3.30580300 0.24022200 -0.13615400

N -3.23246800 0.61540600 -0.61931200

C -1.31735800 -2.28812200 -0.02478600

C 0.89297900 2.79413300 -0.60978900

C 2.63216000 1.40265900 -0.31582000

C -2.74348400 -0.58361600 -0.35042900

C -2.65106000 -2.86273300 -0.22923400

C 2.08022700 3.50982800 -1.02278000

C 3.15018900 2.66474200 -0.83622700

C -3.51383700 -1.83179800 -0.42158700

O -0.12438400 0.45975900 1.97854000

C -2.04252300 -2.09521600 3.30026600

H -1.54146700 -3.02273500 3.01009900

H -1.45665700 -1.24018200 2.95370700

H -3.03664500 -2.06808600 2.84616700

H -2.13998400 -2.05973900 4.38935200

**TS1 (*F*_z1_=+0.003 a.u.)**

Fe -0.12110900 -0.00361600 0.47768900

H 2.50243500 -4.33190000 -0.15365500

H -1.82712800 4.52701100 -0.96196200

H 4.36763000 -2.36647100 -0.20653000

H -4.03248000 2.99408800 -0.95002600

H -2.74612300 -4.30013100 -0.28725700

H 1.70848200 4.49895800 -0.90728600

H 3.89179300 2.93870500 -0.79294700

H -4.59046400 -2.32057300 -0.44466100

N 1.24086600 -1.26086900 0.06199400

N -1.30464300 1.37334300 -0.06057800

N -1.46568400 -1.25033900 0.01190000

N 1.11747700 1.37294600 0.05705800

C 2.35538200 -3.26360700 -0.07865300

C -1.89006800 3.48371300 -0.68518200

C 3.29602900 -2.27289400 -0.09918500

C -3.02819600 2.69581000 -0.68293700

C 1.04925300 -2.63286000 0.01199100

C -0.78678500 2.64773600 -0.30670700

C 2.60548500 -0.99456700 -0.02638500

C -2.64029000 1.35028400 -0.31556800

N -0.12013500 -3.26616800 -0.00143600

N 3.19005700 0.19702400 -0.14002200

N -3.38895300 0.22954400 -0.30751300

C -1.29075000 -2.61614700 -0.03585100

C 0.62333000 2.64313500 -0.25208700

C 2.45639000 1.32070300 -0.17578200

C -2.83265600 -0.96564900 -0.16758900

C -2.59385300 -3.23337300 -0.20029600

C 1.75109000 3.46194200 -0.60434200

C 2.87476800 2.65999100 -0.55413900

C -3.52649900 -2.23439800 -0.27480300

O -0.20887700 -0.02683600 2.19840900

C -2.48200600 -0.66439400 3.18477400

H -2.61340500 -1.67475900 2.80127300

H -1.23039700 -0.30844400 2.65119800

H -3.14683100 0.07742200 2.74598900

H -2.35113800 -0.59036100 4.26310300

**RC (*F*_z1_=+0.004 a.u.)**

Fe -0.04020200 0.30462800 0.38004700

H 2.33289100 -4.21883900 0.18099000

H -1.36565300 4.70827000 -1.72471900

H 4.32264900 -2.37875100 0.06822200

H -3.66003500 3.31187200 -1.68293300

H -2.86702200 -3.90882300 -0.26692500

H 2.10806100 4.49900500 -1.45444600

H 4.18634000 2.84094900 -1.06893300

H -4.58272800 -1.84685800 -0.66866400

N 1.25436800 -1.07083600 0.13550800

N -1.12591000 1.69906700 -0.32271000

N -1.43582100 -0.91029500 -0.07272500

N 1.29915700 1.55388500 -0.13300800

C 2.25884400 -3.14048600 0.18131100

C -1.52292000 3.72330800 -1.30746100

C 3.25513100 -2.21844900 0.12584500

C -2.69973400 3.01142300 -1.28773100

C 0.98558700 -2.41294200 0.16957000

C -0.50394200 2.88120600 -0.71902000

C 2.63317300 -0.88939200 0.07204200

C -2.41724700 1.70577700 -0.69715000

N -0.21823500 -2.99097200 0.13241200

N 3.29441800 0.24064900 -0.11421700

N -3.23987800 0.63146600 -0.63338800

C -1.33522900 -2.27495100 -0.02316000

C 0.89066800 2.79764200 -0.61016000

C 2.62446400 1.40380500 -0.30125900

C -2.75507500 -0.56783600 -0.35918300

C -2.66843300 -2.84687100 -0.23671000

C 2.08082100 3.50665900 -1.02604800

C 3.14770900 2.66010300 -0.82985800

C -3.52775300 -1.81416700 -0.43518800

O -0.14816100 0.47221000 1.98639200

C -1.97882600 -2.13975700 3.31105000

H -1.44983400 -3.05112600 3.01955700

H -1.41714800 -1.26714700 2.96786200

H -2.97131300 -2.14059100 2.85254200

H -2.08183500 -2.10996800 4.40012200

**TS1 (*F*_z1_=+0.004 a.u.)**

Fe -0.11738200 -0.00265900 0.48887200

H 2.45577100 -4.34004600 -0.28032200

H -1.78925400 4.56656400 -0.87073300

H 4.33907900 -2.39183300 -0.31352900

H -4.00936700 3.05514000 -0.86593300

H -2.78875600 -4.25593300 -0.36020900

H 1.73497300 4.50260100 -0.86068700

H 3.90430400 2.92019700 -0.80302800

H -4.61619300 -2.25699500 -0.45724000

N 1.22757700 -1.26262900 0.02981600

N -1.29367900 1.39640600 -0.00935600

N -1.47845100 -1.22669700 0.01063400

N 1.13180800 1.37268100 0.08859500

C 2.32106800 -3.27282400 -0.17360000

C -1.86168400 3.52138100 -0.60356300

C 3.27052100 -2.29133300 -0.18423400

C -3.00717800 2.74489500 -0.60469500

C 1.02200300 -2.63122400 -0.04970100

C -0.76473200 2.66923100 -0.24200200

C 2.59281900 -1.00781800 -0.07180500

C -2.62950400 1.39075000 -0.25761800

N -0.15255700 -3.25347200 -0.06295600

N 3.18802900 0.17953200 -0.16827800

N -3.38986900 0.27669000 -0.26468600

C -1.31730800 -2.59088200 -0.07194400

C 0.64464500 2.65075200 -0.20094600

C 2.46413400 1.31050000 -0.17257400

C -2.84420000 -0.92519800 -0.15428000

C -2.62745500 -3.19333600 -0.24367000

C 1.77397500 3.46333400 -0.56505400

C 2.88977600 2.65042700 -0.54413000

C -3.55111200 -2.18519500 -0.28778900

O -0.19837900 -0.07014800 2.20915500

C -2.46326100 -0.76038500 3.20038600

H -2.32470000 -0.71336700 4.27932500

H -1.20129700 -0.37323000 2.65573500

H -2.58841400 -1.76086900 2.79027200

H -3.13375000 -0.01100900 2.78377200

**RC (*F*_z1_=+0.005 a.u.)**

Fe -0.04342600 0.30808200 0.38979300

H 2.32665400 -4.21704100 0.17213900

H -1.35929100 4.70259800 -1.73931800

H 4.31774600 -2.37786000 0.06310200

H -3.65492400 3.30859400 -1.69801400

H -2.86755100 -3.90464800 -0.28120000

H 2.10554400 4.49215300 -1.46622300

H 4.18318000 2.83440800 -1.07666800

H -4.58229500 -1.84165800 -0.68257300

N 1.25061300 -1.06783000 0.14396100

N -1.12809600 1.70265600 -0.31434600

N -1.43866300 -0.90604500 -0.06676200

N 1.29737100 1.55633300 -0.12224300

C 2.25377000 -3.13863600 0.18120400

C -1.51998900 3.72187300 -1.31346600

C 3.25066800 -2.21713200 0.12753000

C -2.69731800 3.01133700 -1.29393700

C 0.98134100 -2.41006500 0.17389700

C -0.50423900 2.88211900 -0.71597300

C 2.62947200 -0.88767900 0.07924300

C -2.41790600 1.70888300 -0.69495500

N -0.22238000 -2.98783300 0.13566400

N 3.29185400 0.24173100 -0.10572100

N -3.24139700 0.63567400 -0.63160100

C -1.33846900 -2.27105400 -0.02134900

C 0.88957400 2.79800100 -0.60571600

C 2.62225800 1.40465000 -0.29401100

C -2.75703300 -0.56359500 -0.35735400

C -2.67040300 -2.84271500 -0.24194500

C 2.07910600 3.50378100 -1.02866500

C 3.14558200 2.65746000 -0.83029400

C -3.52925800 -1.80961500 -0.44035500

O -0.15224500 0.47217000 1.99742000

C -1.96676900 -2.14722700 3.31445300

H -1.42780900 -3.05310200 3.02418300

H -1.41310100 -1.26926500 2.97153100

H -2.95774000 -2.15864900 2.85286900

H -2.07308100 -2.11798800 4.40357600

**TS1 (*F*_z1_=+0.005 a.u.)**

Fe -0.12338600 0.05389800 0.53677500

H 2.46199000 -4.29988200 -0.10711400

H -1.76062900 4.54537700 -1.10809700

H 4.34202800 -2.35024100 -0.18422400

H -3.97709500 3.02855400 -1.10119800

H -2.76821300 -4.23477200 -0.31788400

H 1.70592700 4.48314100 -1.07974100

H 3.87844700 2.90900200 -0.96316800

H -4.60212500 -2.24715800 -0.50198600

N 1.22531700 -1.21431400 0.11908900

N -1.30475200 1.43689000 -0.01356800

N -1.48517000 -1.19786600 0.08803800

N 1.14076900 1.43765600 0.15331300

C 2.32868900 -3.23026700 -0.02555000

C -1.84540800 3.51972400 -0.77656200

C 3.27265000 -2.25129500 -0.05979400

C -2.98711200 2.74309000 -0.77371900

C 1.02141800 -2.58017700 0.07409300

C -0.76375600 2.69146000 -0.30971800

C 2.58441500 -0.95894700 0.01346700

C -2.61574900 1.41284800 -0.32253800

N -0.14697800 -3.21122600 0.06497600

N 3.18134300 0.22101700 -0.11530700

N -3.38087400 0.29008500 -0.31189800

C -1.31619300 -2.54642400 0.01696600

C 0.63976700 2.68213800 -0.23988600

C 2.45171100 1.35516100 -0.15610900

C -2.83885500 -0.90044200 -0.14360400

C -2.62269100 -3.17009300 -0.19983400

C 1.76003500 3.47249300 -0.69926600

C 2.87344500 2.66731500 -0.64600600

C -3.54499500 -2.17210700 -0.28952400

O -0.27782900 -0.00591500 2.27189900

C -2.46868600 -0.95421900 3.24237400

H -2.48601800 -1.97640200 2.87134100

H -1.21261900 -0.41943400 2.67165000

H -3.20648200 -0.28873500 2.79925000

H -2.32033300 -0.84593100 4.31506100

**RC (*F*_z1_=+0.006 a.u.)**

Fe -0.05030700 0.30600100 0.39432200

H 2.31624600 -4.22200500 0.17639200

H -1.34862700 4.68570400 -1.77519000

H 4.30947000 -2.38457400 0.07320500

H -3.64600300 3.29493400 -1.73734900

H -2.87120500 -3.90687600 -0.29572400

H 2.10670500 4.47419200 -1.48692500

H 4.18193900 2.81750800 -1.08166900

H -4.58314400 -1.84346400 -0.70704700

N 1.24364100 -1.07141400 0.15542900

N -1.13125100 1.69911800 -0.31842700

N -1.44386000 -0.90821900 -0.06741300

N 1.29386500 1.55133200 -0.11622900

C 2.24475800 -3.14356300 0.19164800

C -1.51434100 3.71050300 -1.33868100

C 3.24270700 -2.22297000 0.14084500

C -2.69244100 3.00173100 -1.32089700

C 0.97340800 -2.41366500 0.18336200

C -0.50390200 2.87453700 -0.72678100

C 2.62281300 -0.89309100 0.09310900

C -2.41833200 1.70411500 -0.70906600

N -0.23024900 -2.99093200 0.14190600

N 3.28724300 0.23504700 -0.09129500

N -3.24327900 0.63252600 -0.64514800

C -1.34476500 -2.27346500 -0.02154300

C 0.88872400 2.78977200 -0.61049800

C 2.61894800 1.39740400 -0.28621300

C -2.76058100 -0.56608500 -0.36609500

C -2.67514200 -2.84497500 -0.25030400

C 2.07927400 3.49096400 -1.03795500

C 3.14448900 2.64521400 -0.83137800

C -3.53266200 -1.81172300 -0.45385100

O -0.16572300 0.47169700 2.00233700

C -1.92539300 -2.17945900 3.31951200

H -1.36904700 -3.07421100 3.02772200

H -1.38848000 -1.29072400 2.97724500

H -2.91534600 -2.20939400 2.85655500

H -2.03333000 -2.15366500 4.40892800

**TS1 (*F*_z1_=+0.006 a.u.)**

Fe -0.12770400 0.06815300 0.54867800

H 2.44275800 -4.29501500 -0.09734800

H -1.74210800 4.54864400 -1.14660400

H 4.32937600 -2.35160500 -0.17658300

H -3.96363000 3.03960000 -1.13692800

H -2.78047700 -4.21531700 -0.32364700

H 1.71228600 4.47565500 -1.11196900

H 3.88030000 2.89652200 -0.98374800

H -4.60835200 -2.22273700 -0.51517700

N 1.21646700 -1.20510900 0.13199400

N -1.30449800 1.45233000 -0.00880400

N -1.49339300 -1.18165100 0.10068500

N 1.14254200 1.44799300 0.16519600

C 2.31391600 -3.22518200 -0.01155900

C -1.83358200 3.52833800 -0.80061600

C 3.26082500 -2.24960700 -0.04743200

C -2.97759800 2.75612600 -0.79607700

C 1.00887600 -2.57002700 0.08857100

C -0.75777600 2.70122100 -0.31648200

C 2.57634600 -0.95460100 0.02581600

C -2.61296400 1.42958300 -0.32748000

N -0.16098600 -3.19873000 0.07999400

N 3.17781800 0.22220800 -0.10368600

N -3.38233700 0.30983200 -0.31394300

C -1.32756500 -2.53037900 0.02762200

C 0.64457600 2.68892200 -0.24300500

C 2.45156900 1.35882000 -0.14938400

C -2.84367400 -0.88127300 -0.13970100

C -2.63436200 -3.15157700 -0.19820900

C 1.76565300 3.47099300 -0.71579800

C 2.87663500 2.66364200 -0.65596000

C -3.55317000 -2.15148900 -0.29187700

O -0.28663600 0.00487600 2.28659900

C -2.44900400 -1.01969100 3.25284400

H -2.43440000 -2.04294300 2.88499400

H -1.20464800 -0.44103400 2.67789400

H -3.20728600 -0.37879500 2.80818900

H -2.30179400 -0.90269700 4.32494100

**RC (*F*_z1_=+0.007 a.u.)**

Fe -0.05522400 0.30203600 0.40008800

H 2.30960400 -4.22649000 0.15814900

H -1.34517200 4.67422700 -1.78901700

H 4.30355400 -2.38941000 0.06145800

H -3.64341600 3.28539600 -1.75250700

H -2.87261600 -3.90984300 -0.31652400

H 2.10147700 4.46223600 -1.49805200

H 4.17648900 2.80632200 -1.08967200

H -4.58425400 -1.84544500 -0.72420800

N 1.23849300 -1.07517800 0.15818500

N -1.13550400 1.69580200 -0.31267500

N -1.44798300 -0.91119400 -0.06720900

N 1.28999700 1.54744200 -0.10794900

C 2.23890400 -3.14817100 0.18354600

C -1.51402000 3.70291800 -1.34505400

C 3.23723600 -2.22775300 0.13593400

C -2.69248500 2.99521000 -1.32795100

C 0.96820800 -2.41763900 0.18040700

C -0.50656200 2.86903700 -0.72528100

C 2.61776900 -0.89763900 0.09528100

C -2.42112400 1.70037800 -0.70907600

N -0.23522200 -2.99492300 0.13737100

N 3.28298100 0.23030700 -0.08619200

N -3.24674900 0.62972800 -0.64609900

C -1.34901200 -2.27681600 -0.02640600

C 0.88526900 2.78403600 -0.60749400

C 2.61476000 1.39245300 -0.28130000

C -2.76410700 -0.56903700 -0.36847800

C -2.67820700 -2.84807900 -0.26140200

C 2.07505000 3.48269800 -1.04098300

C 3.14011600 2.63736900 -0.83269500

C -3.53560900 -1.81439900 -0.46332800

O -0.17175200 0.46200400 2.00963700

C -1.87472900 -2.22339700 3.32224500

H -1.30506100 -3.10636000 3.02062400

H -1.35449400 -1.32344900 2.98307600

H -2.86593100 -2.26665500 2.86296400

H -1.97833500 -2.20719500 4.41264600

**TS1 (*F*_z1_=+0.007 a.u.)**

Fe -0.13339800 0.08288400 0.55878000

H 2.42689400 -4.28734600 -0.08545900

H -1.72879600 4.55089200 -1.18499700

H 4.31820400 -2.34834200 -0.16571700

H -3.95394900 3.04765100 -1.17282300

H -2.78962400 -4.19787800 -0.32940400

H 1.71493000 4.47066600 -1.14175600

H 3.87972500 2.88864500 -1.00011100

H -4.61314400 -2.20211100 -0.52932500

N 1.20778800 -1.19425300 0.14425200

N -1.30627500 1.46685200 -0.00690700

N -1.50117100 -1.16619000 0.11101400

N 1.14161400 1.45951700 0.17489800

C 2.30127700 -3.21741600 0.00389600

C -1.82588900 3.53580700 -0.82538800

C 3.25027500 -2.24423400 -0.03298600

C -2.97157100 2.76689200 -0.81938800

C 0.99787000 -2.55863400 0.10295200

C -0.75512100 2.71083500 -0.32516400

C 2.56836900 -0.94738000 0.03903400

C -2.61254600 1.44435400 -0.33459400

N -0.17287300 -3.18594500 0.09442900

N 3.17336600 0.22694500 -0.09098500

N -3.38495000 0.32701500 -0.31805100

C -1.33739800 -2.51522100 0.03698600

C 0.64611500 2.69682100 -0.24741500

C 2.44952500 1.36510900 -0.14258700

C -2.84863500 -0.86421200 -0.13774000

C -2.64375700 -3.13491400 -0.19733300

C 1.76785800 3.47180700 -0.73105800

C 2.87716300 2.66325500 -0.66381800

C -3.56006100 -2.13350200 -0.29540300

O -0.29606400 0.01638500 2.29928800

C -2.42378600 -1.08861500 3.26507500

H -2.36896200 -2.11397400 2.90730000

H -1.19528100 -0.46215100 2.68315300

H -3.20543800 -0.48163300 2.81352500

H -2.28028400 -0.95438100 4.33582700

**RC (*F*_z1_=+0.008 a.u.)**

Fe -0.05920000 0.29688600 0.40530900

H 2.30235700 -4.23370200 0.15109400

H -1.33637000 4.65719900 -1.81424500

H 4.29787700 -2.39800600 0.05921200

H -3.63604900 3.27130000 -1.77874200

H -2.87407100 -3.91436000 -0.33058400

H 2.10139600 4.44413600 -1.51565400

H 4.17496400 2.78912200 -1.09795500

H -4.58389400 -1.84925800 -0.74174100

N 1.23410600 -1.08131300 0.16560900

N -1.13732300 1.69007800 -0.31198300

N -1.45103300 -0.91588800 -0.06600000

N 1.28821300 1.54044000 -0.10198700

C 2.23289000 -3.15550600 0.18464000

C -1.50923400 3.69078900 -1.36117400

C 3.23203500 -2.23577000 0.13935400

C -2.68837800 2.98467500 -1.34452300

C 0.96314800 -2.42383400 0.18432000

C -0.50582900 2.86000100 -0.73056100

C 2.61357300 -0.90526400 0.10271100

C -2.42098000 1.69387700 -0.71560200

N -0.24024800 -3.00070400 0.13984500

N 3.28028700 0.22181700 -0.07764600

N -3.24780200 0.62462700 -0.65193300

C -1.35290200 -2.28182400 -0.02684000

C 0.88504400 2.77439600 -0.60964000

C 2.61286400 1.38361200 -0.27627600

C -2.76612500 -0.57379300 -0.37222000

C -2.68084500 -2.85276400 -0.26816500

C 2.07494600 3.46921100 -1.04877200

C 3.13926700 2.62435900 -0.83553500

C -3.53744800 -1.81874800 -0.47200300

O -0.17911200 0.45576600 2.01581500

C -1.82517800 -2.25912400 3.32868500

H -1.24295200 -3.13061000 3.01792700

H -1.32237500 -1.34893900 2.99003200

H -2.81826900 -2.31684700 2.87503600

H -1.92212300 -2.25048600 4.42019900

**TS1 (*F*_z1_=+0.008 a.u.)**

Fe -0.14069100 0.09956300 0.56850600

H 2.40486700 -4.28078100 -0.07249800

H -1.71197400 4.55509700 -1.22623500

H 4.30284700 -2.34815400 -0.15430100

H -3.94231600 3.06007900 -1.21069100

H -2.80468700 -4.17610500 -0.33313400

H 1.72106100 4.46436000 -1.17326400

H 3.88086800 2.87734700 -1.01673700

H -4.62162200 -2.17529300 -0.54216600

N 1.19621000 -1.18322300 0.15691000

N -1.30797300 1.48425200 -0.00596800

N -1.51161800 -1.14759700 0.12161100

N 1.14051400 1.47132500 0.18351300

C 2.28356400 -3.21063400 0.02025400

C -1.81581300 3.54579200 -0.85244900

C 3.23568000 -2.24079200 -0.01790000

C -2.96399900 2.78139400 -0.84454800

C 0.98257800 -2.54693100 0.11808600

C -0.75108500 2.72246200 -0.33559200

C 2.55775600 -0.94145700 0.05260600

C -2.61215500 1.46307500 -0.34274000

N -0.18972100 -3.17158500 0.10991000

N 3.16753400 0.22973300 -0.07824700

N -3.38869000 0.34910700 -0.32238300

C -1.35141500 -2.49730900 0.04723100

C 0.64900300 2.70530300 -0.25346700

C 2.44741800 1.37011800 -0.13634500

C -2.85603600 -0.84273600 -0.13537600

C -2.65790600 -3.11404600 -0.19497800

C 1.77233000 3.47160500 -0.74774900

C 2.87909600 2.66072600 -0.67240200

C -3.57065800 -2.11032400 -0.29775800

O -0.30668400 0.03049400 2.31136100

C -2.38875800 -1.16741800 3.27915200

H -2.28504000 -2.19286300 2.93286400

H -1.18299100 -0.48464100 2.68814600

H -3.19755600 -0.60274300 2.82075800

H -2.24993700 -1.01319900 4.34802100

**RC (*F*_z1_=+0.009 a.u.)**

Fe -0.06118900 0.28773400 0.41036200

H 2.29969000 -4.24397300 0.15285300

H -1.32565700 4.63186300 -1.84741800

H 4.29591300 -2.40877100 0.06490800

H -3.62615800 3.24789700 -1.81264300

H -2.87060000 -3.92544500 -0.34088100

H 2.10289100 4.42000800 -1.53772800

H 4.17551400 2.76773500 -1.10618200

H -4.57934600 -1.86113500 -0.75999800

N 1.23266500 -1.09110400 0.17620200

N -1.13719600 1.67866200 -0.31468900

N -1.45098600 -0.92602900 -0.06466500

N 1.28824100 1.52939300 -0.09679300

C 2.23087000 -3.16593400 0.19288900

C -1.50249700 3.67104500 -1.38403800

C 3.23041000 -2.24641800 0.14951500

C -2.68199900 2.96600700 -1.36772100

C 0.96172400 -2.43379600 0.19308000

C -0.50335000 2.84489600 -0.74045600

C 2.61231100 -0.91591300 0.11433500

C -2.41850600 1.68038900 -0.72663500

N -0.24130300 -3.01097700 0.14684000

N 3.28008400 0.21037900 -0.06599800

N -3.24605900 0.61224900 -0.66134400

C -1.35302200 -2.29216700 -0.02487800

C 0.88647200 2.75965500 -0.61484100

C 2.61303600 1.37116200 -0.27057900

C -2.76495400 -0.58515900 -0.37733900

C -2.67922400 -2.86384800 -0.27267700

C 2.07651000 3.45049500 -1.05963700

C 3.14034600 2.60706200 -0.83911600

C -3.53538400 -1.83025200 -0.48071500

O -0.18608700 0.44760200 2.02153300

C -1.76653800 -2.29801300 3.33905400

H -1.17114300 -3.15742400 3.01987900

H -1.28304100 -1.37770100 2.99910200

H -2.76159400 -2.37235700 2.89208900

H -1.85557500 -2.29509100 4.43171300

**TS1 (*F*_z1_=+0.009 a.u.)**

Fe -0.15352200 0.12233300 0.57701400

H 2.37502400 -4.27172200 -0.04506500

H -1.69281100 4.56233200 -1.28023900

H 4.28138900 -2.34711800 -0.12775100

H -3.92954200 3.07748900 -1.26307200

H -2.82681500 -4.14794200 -0.33586200

H 1.72969300 4.45868600 -1.20912700

H 3.88260800 2.86524200 -1.02944200

H -4.63472400 -2.14084200 -0.56180000

N 1.17869000 -1.16825000 0.17303200

N -1.31248900 1.50746400 -0.01126200

N -1.52757300 -1.12298100 0.13100800

N 1.13582200 1.48728500 0.19129600

C 2.25861400 -3.20106400 0.04820000

C -1.80500900 3.55983400 -0.89079000

C 3.21469400 -2.23535900 0.00902500

C -2.95635500 2.80094400 -0.88189800

C 0.96039100 -2.53123600 0.13966400

C -0.74816100 2.73861400 -0.35351600

C 2.54160500 -0.93289700 0.07320600

C -2.61397500 1.48786500 -0.35983100

N -0.21393700 -3.15236400 0.13057800

N 3.15756600 0.23439100 -0.05889900

N -3.39547300 0.37804400 -0.33517000

C -1.37202600 -2.47369600 0.05877700

C 0.65052800 2.71741700 -0.26373400

C 2.44245000 1.37748500 -0.12741700

C -2.86802400 -0.81450100 -0.13785700

C -2.67851400 -3.08669100 -0.19324200

C 1.77731300 3.47293300 -0.76714500

C 2.88061400 2.65896900 -0.67941700

C -3.58653600 -2.08002800 -0.30455400

O -0.32343700 0.05215100 2.32231200

C -2.32811300 -1.27637600 3.29811800

H -2.13750800 -2.30058200 2.98687100

H -1.16535400 -0.51286300 2.69274400

H -3.17515800 -0.79423500 2.81548700

H -2.20994600 -1.07473100 4.36175700

**RC (*F*_z1_=+0.010 a.u.)**

Fe -0.06513400 0.28166000 0.41420100

H 2.29490800 -4.25152000 0.15977300

H -1.31494800 4.60745200 -1.88606700

H 4.29197600 -2.41703600 0.07524700

H -3.61624900 3.22543700 -1.85273400

H -2.86892100 -3.93393900 -0.35008600

H 2.10394000 4.39687400 -1.56308900

H 4.17510600 2.74747500 -1.11554100

H -4.57600900 -1.87070300 -0.78047900

N 1.22926700 -1.09812100 0.18725700

N -1.13851200 1.66989700 -0.32010600

N -1.45271300 -0.93345300 -0.06420100

N 1.28670700 1.52097900 -0.09283000

C 2.22673600 -3.17361500 0.20497200

C -1.49619100 3.65274800 -1.41179700

C 3.22676300 -2.25441000 0.16326300

C -2.67604000 2.94877700 -1.39621600

C 0.95822400 -2.44095700 0.20383700

C -0.50189800 2.83191000 -0.75378300

C 2.60910900 -0.92392000 0.12745200

C -2.41709400 1.66909400 -0.74149300

N -0.24443700 -3.01840300 0.15567900

N 3.27815200 0.20137600 -0.05343900

N -3.24544200 0.60216600 -0.67435100

C -1.35506200 -2.29975400 -0.02245800

C 0.88676700 2.74706600 -0.62258100

C 2.61170300 1.36099700 -0.26543200

C -2.76525200 -0.59400000 -0.38462500

C -2.67937300 -2.87231100 -0.27704100

C 2.07723400 3.43329700 -1.07308100

C 3.14034600 2.59133000 -0.84416400

C -3.53481100 -1.83926400 -0.49098400

O -0.19533800 0.44330400 2.02596500

C -1.70598900 -2.33264300 3.35104700

H -1.10080800 -3.17977900 3.01796900

H -1.24263400 -1.40240100 3.00945900

H -2.70533900 -2.42119400 2.91630100

H -1.78053800 -2.33763500 4.44525300

**TS1 (*F*_z1_=+0.010 a.u.)**

Fe -0.16484900 0.14135900 0.58582400

H 2.34944700 -4.26396700 -0.02461200

H -1.67569400 4.56578900 -1.32857400

H 4.26282400 -2.34608500 -0.10656900

H -3.91780800 3.08973300 -1.30997800

H -2.84484700 -4.12433300 -0.33973700

H 1.73649000 4.45191200 -1.24013100

H 3.88352800 2.85356200 -1.03952800

H -4.64518100 -2.11211400 -0.57935400

N 1.16362300 -1.15589000 0.18884400

N -1.31682400 1.52687100 -0.01373000

N -1.54109200 -1.10237800 0.13951600

N 1.13134000 1.50035600 0.19914900

C 2.23727800 -3.19306700 0.07121100

C -1.79552800 3.57003200 -0.92428900

C 3.19669700 -2.23081800 0.03193100

C -2.94951900 2.81589000 -0.91457200

C 0.94149900 -2.51818300 0.15855800

C -0.74592400 2.75146300 -0.36800500

C 2.52770900 -0.92588000 0.09235300

C -2.61567500 1.50813000 -0.37360200

N -0.23450200 -3.13640700 0.14820700

N 3.14882500 0.23809900 -0.04048100

N -3.40131400 0.40197100 -0.34524100

C -1.38938800 -2.45408100 0.06866500

C 0.65143500 2.72685700 -0.27177900

C 2.43786300 1.38330400 -0.11830100

C -2.87816900 -0.79105600 -0.13934800

C -2.69559200 -3.06390100 -0.19213200

C 1.78107800 3.47278600 -0.78325800

C 2.88149200 2.65646700 -0.68433100

C -3.59969800 -2.05480300 -0.31036700

O -0.33934300 0.07016700 2.33318100

C -2.27650100 -1.36447900 3.31348900

H -2.01714800 -2.37846000 3.01897000

H -1.15094300 -0.53307500 2.69680100

H -3.15004600 -0.94537400 2.81966400

H -2.17354200 -1.13639800 4.37355700

**RC (*F*_z1_=-0.001 a.u.)**

Fe 0.15222700 0.13138100 0.39187400

H 2.71111900 -4.24569800 -0.20691200

H -1.54237000 4.64100300 -1.15137100

H 4.60072300 -2.30600600 -0.34682400

H -3.76022600 3.12597300 -1.06672300

H -2.54043200 -4.16367100 -0.24912000

H 1.98324900 4.58572500 -1.15415200

H 4.15317200 3.00320600 -1.06777300

H -4.36843400 -2.16549000 -0.39148100

N 1.48401600 -1.15783100 -0.04263100

N -1.04555100 1.52027400 -0.11552300

N -1.22310800 -1.11573900 -0.03404000

N 1.38945500 1.48197000 -0.12044300

C 2.58388800 -3.17300600 -0.16901500

C -1.61477300 3.60624900 -0.84624200

C 3.53040800 -2.20068500 -0.23777100

C -2.75318500 2.83317900 -0.80433900

C 1.27857700 -2.51035900 -0.06249800

C -0.51069000 2.76621200 -0.43593000

C 2.84567000 -0.90207100 -0.18021000

C -2.36358200 1.49203800 -0.37599700

N 0.09984900 -3.14176200 -0.04827300

N 3.43754500 0.27147200 -0.33366300

N -3.12877000 0.37297600 -0.34363000

C -1.05844100 -2.47425900 -0.06839500

C 0.89362600 2.74396400 -0.43867300

C 2.70626800 1.41313900 -0.37760200

C -2.57407800 -0.81818600 -0.19019500

C -2.38142500 -3.09573100 -0.19966100

C 2.02329300 3.54913000 -0.84929200

C 3.13732900 2.74114300 -0.80687400

C -3.29661800 -2.09470300 -0.27083300

O 0.17115400 0.18427300 2.00525600

C -2.89709200 -0.95564400 3.18829500

H -3.02361300 -1.98936500 2.85493700

H -1.90023700 -0.60009600 2.91707300

H -3.65353700 -0.32721400 2.71023000

H -3.02134900 -0.90958100 4.27320400

**TS1 (*F*_z1_=-0.001 a.u.)**

Fe -0.12596200 -0.03206200 0.43026100

H 2.52386200 -4.34877200 -0.13006300

H -1.86869700 4.51824300 -0.88095000

H 4.37999800 -2.37405800 -0.16858300

H -4.06626200 2.97293600 -0.88549200

H -2.73841700 -4.34113000 -0.28146300

H 1.70172400 4.51121700 -0.79861500

H 3.89167800 2.95865300 -0.68544200

H -4.59006100 -2.36786200 -0.43381200

N 1.24345600 -1.28410800 0.02097500

N -1.31385000 1.34194700 -0.09953700

N -1.46582600 -1.28348800 -0.03563200

N 1.10222600 1.34673700 0.00396000

C 2.36790100 -3.28001800 -0.08539900

C -1.91923900 3.46313100 -0.64992100

C 3.30509600 -2.28319800 -0.09863800

C -3.05448800 2.66803300 -0.65667900

C 1.05895900 -2.65760100 -0.02088400

C -0.80607400 2.62427900 -0.31368300

C 2.60932800 -1.00990200 -0.04697800

C -2.65717600 1.31540900 -0.33396400

N -0.11021000 -3.29489700 -0.04290700

N 3.18751300 0.18724200 -0.15085400

N -3.39722300 0.19197800 -0.33381300

C -1.28361700 -2.65328800 -0.07577000

C 0.60833600 2.62443500 -0.26094600

C 2.45149000 1.30824200 -0.18457900

C -2.83408900 -1.00480900 -0.20042100

C -2.58497200 -3.27267400 -0.22106500

C 1.73952200 3.45903900 -0.55246100

C 2.86864900 2.66049900 -0.50223900

C -3.52235300 -2.27512500 -0.29288000

O -0.17213000 -0.03363800 2.15548500

C -2.44827300 -0.51341900 3.16887100

H -2.63102700 -1.53531300 2.83999500

H -1.24461600 -0.24940800 2.64426500

H -3.09709300 0.23155200 2.71160400

H -2.29608300 -0.39480200 4.24025400

**RC (*F*_z1_=-0.002 a.u.)**

Fe 0.16363700 0.11150700 0.38843100

H 2.73939400 -4.25064400 -0.23369000

H -1.55926200 4.63309400 -1.08779500

H 4.62020200 -2.30228400 -0.36868700

H -3.76994800 3.10722700 -1.00967100

H -2.51809200 -4.18958000 -0.24584900

H 1.97344600 4.59255600 -1.10477400

H 4.14943200 3.01719600 -1.04384000

H -4.35403100 -2.19739800 -0.36770600

N 1.49865300 -1.16902300 -0.06156300

N -1.04212500 1.50040200 -0.10046800

N -1.20917200 -1.13770900 -0.03988400

N 1.39267700 1.47184300 -0.11743300

C 2.60688300 -3.17855300 -0.19655100

C -1.62452800 3.59291900 -0.79992800

C 3.54910400 -2.20177400 -0.26276900

C -2.75937600 2.81418600 -0.76144500

C 1.29853700 -2.52214400 -0.08585600

C -0.51402900 2.75257500 -0.40771800

C 2.85886700 -0.90617200 -0.19913700

C -2.36161800 1.46917900 -0.35262500

N 0.12199300 -3.15809500 -0.07014900

N 3.44500000 0.27132600 -0.34606400

N -3.12185000 0.34639600 -0.32673500

C -1.03942300 -2.49538500 -0.07921200

C 0.89090100 2.73609800 -0.41697900

C 2.70926600 1.41096300 -0.37684000

C -2.56225100 -0.84391600 -0.18335300

C -2.36170200 -3.12107800 -0.19999200

C 2.01702000 3.55082200 -0.81842100

C 3.13430900 2.74639800 -0.78906900

C -3.28098400 -2.12294500 -0.26090000

O 0.19320200 0.14921900 2.00134000

C -2.94536300 -0.83510000 3.17443400

H -3.10590300 -1.86343000 2.83855400

H -1.93541600 -0.51425700 2.90840800

H -3.67802600 -0.18004800 2.69517500

H -3.07170300 -0.78779400 4.25880300

**TS1 (*F*_z1_=-0.002 a.u.)**

Fe -0.12737000 -0.03836100 0.41896200

H 2.52735300 -4.35270900 -0.12519900

H -1.87705700 4.51662400 -0.86211600

H 4.38178900 -2.37629700 -0.15975100

H -4.07318000 2.96900800 -0.87135900

H -2.73771600 -4.34939700 -0.28268600

H 1.70028900 4.51355200 -0.77285100

H 3.89138500 2.96223100 -0.66084200

H -4.59066600 -2.37718900 -0.43309500

N 1.24344800 -1.28923600 0.01022000

N -1.31608200 1.33559300 -0.10791700

N -1.46648500 -1.29041400 -0.04714600

N 1.09899100 1.34118600 -0.00793900

C 2.36971200 -3.28391100 -0.08792400

C -1.92515300 3.45905500 -0.64194900

C 3.30627000 -2.28592900 -0.09920300

C -3.05991800 2.66259200 -0.65122600

C 1.06023000 -2.66301700 -0.02982900

C -0.81014200 2.61945800 -0.31480400

C 2.60951200 -1.01353000 -0.05259200

C -2.66085100 1.30848900 -0.33839500

N -0.10893200 -3.30100500 -0.05407800

N 3.18658400 0.18470600 -0.15383900

N -3.39926400 0.18449300 -0.34061300

C -1.28289500 -2.66093600 -0.08637200

C 0.60520300 2.62038700 -0.26256900

C 2.45021100 1.30531200 -0.18678400

C -2.83487100 -1.01269900 -0.20886400

C -2.58395100 -3.28066800 -0.22801400

C 1.73710000 3.45820600 -0.54041400

C 2.86723800 2.66021900 -0.49061900

C -3.52217200 -2.28329800 -0.29888400

O -0.16242200 -0.03724900 2.14511400

C -2.43904700 -0.48122300 3.16952800

H -2.63554400 -1.50489200 2.85415200

H -1.24603900 -0.23768400 2.64388000

H -3.08428300 0.26512600 2.70932900

H -2.27927600 -0.35259300 4.23868900

**RC (*F*_z1_=-0.003 a.u.)**

Fe 0.16283800 0.12017800 0.38300900

H 2.73459500 -4.24780100 -0.20682900

H -1.55674300 4.64776300 -1.07887300

H 4.61795200 -2.30269200 -0.34692400

H -3.76921400 3.12461700 -0.99911900

H -2.52707300 -4.17890200 -0.22380800

H 1.98442600 4.60176800 -1.09447300

H 4.15751400 3.02259300 -1.03014500

H -4.35965500 -2.18436600 -0.35114400

N 1.49663100 -1.16405500 -0.05970900

N -1.04019900 1.50983400 -0.11008200

N -1.21209800 -1.12859600 -0.03889600

N 1.39383600 1.47755700 -0.12624600

C 2.60262100 -3.17530800 -0.18067300

C -1.62162500 3.60538000 -0.79895800

C 3.54625900 -2.19994300 -0.24941500

C -2.75758100 2.82788900 -0.75967500

C 1.29446800 -2.51683700 -0.07760300

C -0.51074800 2.76226400 -0.41369100

C 2.85759800 -0.90298200 -0.19494400

C -2.36056200 1.48078900 -0.35654600

N 0.11652900 -3.15071900 -0.06179800

N 3.44509900 0.27361300 -0.34512300

N -3.12220600 0.35859000 -0.32846300

C -1.04429100 -2.48651300 -0.07248400

C 0.89480200 2.74360100 -0.42248100

C 2.71143900 1.41488600 -0.37891200

C -2.56490900 -0.83252800 -0.18143900

C -2.36813300 -3.11038100 -0.18803200

C 2.02417800 3.55773100 -0.81600500

C 3.14017200 2.75115500 -0.78500500

C -3.28583900 -2.11092100 -0.25158200

O 0.19104700 0.16800800 1.99496100

C -2.93717900 -0.85222500 3.17845200

H -3.09398300 -1.88285500 2.84786800

H -1.92754500 -0.52937600 2.91395300

H -3.67083200 -0.20123700 2.69502400

H -3.06595200 -0.79998700 4.26213300

**TS1 (*F*_z1_=-0.003 a.u.)**

Fe -0.12889700 -0.04501300 0.40772100

H 2.53142100 -4.35642300 -0.12086800

H -1.88590200 4.51404500 -0.84425500

H 4.38381800 -2.37802500 -0.15092900

H -4.08029200 2.96374500 -0.85872900

H -2.73621400 -4.35838400 -0.28504900

H 1.69789700 4.51565600 -0.74672000

H 3.89038800 2.96600900 -0.63566900

H -4.59082600 -2.38759600 -0.43346200

N 1.24359500 -1.29440200 -0.00043500

N -1.31868000 1.32881300 -0.11583700

N -1.46700600 -1.29787900 -0.05885000

N 1.09552200 1.33558800 -0.01990600

C 2.37200200 -3.28765000 -0.09080800

C -1.93149800 3.45419200 -0.63467300

C 3.30774100 -2.28837700 -0.09983900

C -3.06562400 2.65617700 -0.64672600

C 1.06186800 -2.66846200 -0.03904100

C -0.81466300 2.61420600 -0.31580200

C 2.60976000 -1.01695400 -0.05807100

C -2.66468100 1.30082100 -0.34314300

N -0.10714300 -3.30737200 -0.06571700

N 3.18555200 0.18243600 -0.15653100

N -3.40132100 0.17611200 -0.34791600

C -1.28178200 -2.66901200 -0.09743100

C 0.60157600 2.61608800 -0.26419300

C 2.44859600 1.30254000 -0.18879200

C -2.83550300 -1.02134200 -0.21774100

C -2.58240600 -3.28938500 -0.23585000

C 1.73401000 3.45730500 -0.52815600

C 2.86527200 2.66010200 -0.47870200

C -3.52162700 -2.29239800 -0.30574400

O -0.15238500 -0.04188600 2.13456600

C -2.42986700 -0.44666100 3.17219200

H -2.64135900 -1.47184700 2.87155500

H -1.24691300 -0.22479500 2.64400300

H -3.07097800 0.30134800 2.70879800

H -2.26144800 -0.30732200 4.23874900

**RC (*F*_z1_=-0.004 a.u.)**

Fe 0.16698100 0.11733500 0.37827400

H 2.74271000 -4.24826600 -0.20141800

H -1.56168100 4.65324700 -1.04698300

H 4.62419700 -2.30179600 -0.34244500

H -3.77243800 3.12766600 -0.96857600

H -2.52376600 -4.18268300 -0.20897000

H 1.98746800 4.60965000 -1.06771200

H 4.16105400 3.03097900 -1.01172100

H -4.35731800 -2.18899600 -0.33107100

N 1.50116900 -1.16573200 -0.06655100

N -1.03758000 1.50756800 -0.10964000

N -1.20840800 -1.13189800 -0.04060200

N 1.39587300 1.47676700 -0.13053600

C 2.60902300 -3.17582200 -0.18225500

C -1.62357200 3.60752100 -0.77904400

C 3.55187200 -2.19958100 -0.25141500

C -2.75883400 2.82862800 -0.74051100

C 1.29981000 -2.51853800 -0.08221500

C -0.50993800 2.76257700 -0.40566900

C 2.86203000 -0.90296700 -0.20015400

C -2.35908600 1.47872600 -0.34900500

N 0.12184400 -3.15289100 -0.06567600

N 3.44814500 0.27464100 -0.34954300

N -3.11966800 0.35546500 -0.32218200

C -1.03981400 -2.48965000 -0.07278500

C 0.89621900 2.74483100 -0.41697400

C 2.71402200 1.41613000 -0.37987200

C -2.56185300 -0.83586200 -0.17719100

C -2.36433400 -3.11399300 -0.18071900

C 2.02621600 3.56229400 -0.80176700

C 3.14268000 2.75579200 -0.77509800

C -3.28265500 -2.11481300 -0.24160400

O 0.19940400 0.16474700 1.98949600

C -2.95101900 -0.81314100 3.17522700

H -3.11903700 -1.84271700 2.84662100

H -1.93717100 -0.50237700 2.91243700

H -3.67668000 -0.15445700 2.69010800

H -3.08047400 -0.75790500 4.25849400

**TS1 (*F*_z1_=-0.004 a.u.)**

Fe -0.13072200 -0.05163300 0.39661400

H 2.53497600 -4.36010200 -0.11695900

H -1.89427000 4.51144800 -0.82697300

H 4.38540700 -2.37982400 -0.14182800

H -4.08701200 2.95861900 -0.84743300

H -2.73489700 -4.36701200 -0.29028000

H 1.69536100 4.51742200 -0.72055200

H 3.88911900 2.96931800 -0.61047900

H -4.59107800 -2.39754700 -0.43660900

N 1.24343900 -1.29949600 -0.01115300

N -1.32142500 1.32224600 -0.12352800

N -1.46778900 -1.30518700 -0.07074900

N 1.09204000 1.33015500 -0.03114400

C 2.37390800 -3.29138900 -0.09392500

C -1.93755900 3.44945500 -0.62785900

C 3.30884600 -2.29089700 -0.10036400

C -3.07108100 2.64997300 -0.64306300

C 1.06312400 -2.67379900 -0.04862500

C -0.81912500 2.60909200 -0.31661800

C 2.60967500 -1.02038500 -0.06334900

C -2.66844200 1.29344700 -0.34833700

N -0.10569600 -3.31359100 -0.07818600

N 3.18429300 0.18009000 -0.15877500

N -3.40342200 0.16799900 -0.35618500

C -1.28098300 -2.67679200 -0.10944800

C 0.59792600 2.61185000 -0.26537800

C 2.44676500 1.29973400 -0.19032400

C -2.83631200 -1.02968800 -0.22760800

C -2.58113700 -3.29778500 -0.24581000

C 1.73086100 3.45628100 -0.51574500

C 2.86314700 2.65981400 -0.46662700

C -3.52128000 -2.30117300 -0.31470200

O -0.14263700 -0.04704200 2.12378300

C -2.41982200 -0.41268400 3.17815100

H -2.64427200 -1.44107200 2.89833000

H -1.24721200 -0.21235600 2.64487300

H -3.05871200 0.33305400 2.70792200

H -2.24200500 -0.25654300 4.24094200

**RC (*F*_z1_=-0.005 a.u.)**

Fe 0.16988100 0.11722900 0.37382100

H 2.74744700 -4.24777200 -0.19221000

H -1.56406400 4.66106000 -1.01945200

H 4.62820200 -2.30113700 -0.33450900

H -3.77398500 3.13430100 -0.94310000

H -2.52360300 -4.18254200 -0.19233600

H 1.99249200 4.61787800 -1.04323300

H 4.16564300 3.03854000 -0.99362200

H -4.35688700 -2.18890300 -0.31154100

N 1.50404100 -1.16572000 -0.07165900

N -1.03508400 1.50843000 -0.11030200

N -1.20626000 -1.13195400 -0.04236700

N 1.39788100 1.47779200 -0.13436500

C 2.61266400 -3.17533800 -0.18065200

C -1.62381500 3.61238000 -0.76272500

C 3.55530900 -2.19879100 -0.25041900

C -2.75885500 2.83272600 -0.72528200

C 1.30274200 -2.51842500 -0.08448800

C -0.50830200 2.76546400 -0.39942300

C 2.86500600 -0.90213300 -0.20329900

C -2.35748900 1.48028500 -0.34382200

N 0.12441400 -3.15261800 -0.06732500

N 3.45047200 0.27606100 -0.35237500

N -3.11770400 0.35642100 -0.31786800

C -1.03768200 -2.48965100 -0.07190700

C 0.89843500 2.74781200 -0.41241000

C 2.71657100 1.41814400 -0.38015000

C -2.56017100 -0.83531200 -0.17374200

C -2.36311000 -3.11380600 -0.17240300

C 2.02960600 3.56756300 -0.78889000

C 3.14605200 2.76051600 -0.76560100

C -3.28146800 -2.11444600 -0.23176200

O 0.20495900 0.16460000 1.98434800

C -2.95837500 -0.78734000 3.17467800

H -3.12951400 -1.81718900 2.84832400

H -1.94303800 -0.48054700 2.91302800

H -3.68161600 -0.12688000 2.68826700

H -3.08836400 -0.72996500 4.25761200

**TS1 (*F*_z1_=-0.005 a.u.)**

Fe -0.13277200 -0.05841600 0.38525100

H 2.53826500 -4.36384500 -0.11330500

H -1.90253400 4.50867900 -0.80977400

H 4.38673200 -2.38173900 -0.13218600

H -4.09363300 2.95340300 -0.83666700

H -2.73368000 -4.37565300 -0.29728100

H 1.69265500 4.51888200 -0.69391800

H 3.88761400 2.97230900 -0.58466900

H -4.59142000 -2.40751900 -0.44137400

N 1.24311200 -1.30465700 -0.02220700

N -1.32427900 1.31558800 -0.13148700

N -1.46873100 -1.31258300 -0.08336300

N 1.08851200 1.32471100 -0.04229600

C 2.37562900 -3.29522600 -0.09725600

C -1.94357700 3.44464000 -0.62124500

C 3.30976000 -2.29355100 -0.10068100

C -3.07649900 2.64372900 -0.63983700

C 1.06418300 -2.67918000 -0.05873200

C -0.82361300 2.60388900 -0.31754100

C 2.60938800 -1.02389400 -0.06850900

C -2.67220600 1.28604300 -0.35400900

N -0.10441000 -3.31986200 -0.09151400

N 3.18288700 0.17763000 -0.16062500

N -3.40559800 0.15984300 -0.36519700

C -1.28035300 -2.68456400 -0.12246200

C 0.59419800 2.60751700 -0.26645100

C 2.44475400 1.29684000 -0.19157400

C -2.83724300 -1.03806000 -0.23839100

C -2.58003000 -3.30622000 -0.25723900

C 1.72762200 3.45511000 -0.50301200

C 2.86088300 2.65938100 -0.45415000

C -3.52107800 -2.31000800 -0.32505400

O -0.13310900 -0.05322100 2.11245600

C -2.40900600 -0.37795900 3.18651600

H -2.64560300 -1.41019000 2.93170600

H -1.24705400 -0.19973800 2.64626500

H -3.04689000 0.36308600 2.70740200

H -2.22087900 -0.20119700 4.24441200

**RC (*F*_z1_=-0.006 a.u.)**

Fe 0.17282200 0.11759900 0.36899000

H 2.75058900 -4.24784600 -0.18102400

H -1.56500000 4.66988600 -0.99136600

H 4.63134000 -2.30181000 -0.32489500

H -3.77467200 3.14293000 -0.91613000

H -2.52500000 -4.18098100 -0.17536600

H 1.99936900 4.62565400 -1.01871000

H 4.17144800 3.04488600 -0.97512500

H -4.35731300 -2.18666100 -0.29137300

N 1.50641000 -1.16583500 -0.07683200

N -1.03207500 1.51010800 -0.11167600

N -1.20468200 -1.13116800 -0.04386000

N 1.40032900 1.47866900 -0.13906100

C 2.61510200 -3.17542200 -0.17768100

C -1.62295400 3.61833700 -0.74612500

C 3.55792600 -2.19893400 -0.24819000

C -2.75809500 2.83840100 -0.70931300

C 1.30466800 -2.51840300 -0.08614100

C -0.50572800 2.76893600 -0.39379100

C 2.86763600 -0.90195800 -0.20607000

C -2.35552500 1.48321200 -0.33840400

N 0.12572300 -3.15196200 -0.06839400

N 3.45290400 0.27658100 -0.35500400

N -3.11577500 0.35900600 -0.31295400

C -1.03659500 -2.48883600 -0.07069200

C 0.90162200 2.75085600 -0.40851900

C 2.71965400 1.41954400 -0.38076100

C -2.55892700 -0.83336100 -0.16982500

C -2.36315400 -3.11230600 -0.16373400

C 2.03436000 3.57243000 -0.77627300

C 3.15051600 2.76443600 -0.75611900

C -3.28118100 -2.11241800 -0.22144000

O 0.21053100 0.16568600 1.97883700

C -2.96551800 -0.76176200 3.17410200

H -3.14051900 -1.79182800 2.85027100

H -1.94871400 -0.45950300 2.91285600

H -3.68628200 -0.09924900 2.68668300

H -3.09533100 -0.70195600 4.25677500

**TS1 (*F*_z1_=-0.006 a.u.)**

Fe -0.13507300 -0.06571600 0.37399200

H 2.54120400 -4.36787900 -0.11057700

H -1.91032900 4.50554800 -0.79246300

H 4.38773000 -2.38401700 -0.12236700

H -4.09980900 2.94792200 -0.82699000

H -2.73257200 -4.38440800 -0.30722500

H 1.68970500 4.51973300 -0.66673200

H 3.88577600 2.97457100 -0.55881700

H -4.59182400 -2.41754800 -0.44881700

N 1.24255700 -1.31012200 -0.03359100

N -1.32725500 1.30868600 -0.13877800

N -1.46980900 -1.32025500 -0.09691400

N 1.08501800 1.31908600 -0.05227100

C 2.37711100 -3.29938500 -0.10126300

C -1.94929400 3.43958100 -0.61451800

C 3.31043000 -2.29658700 -0.10109100

C -3.08161100 2.63729000 -0.63714200

C 1.06498400 -2.68480800 -0.06969000

C -0.82802800 2.59844700 -0.31767100

C 2.60885900 -1.02773200 -0.07357300

C -2.67583400 1.27847700 -0.35996200

N -0.10332000 -3.32638600 -0.10627000

N 3.18129400 0.17477900 -0.16208100

N -3.40774500 0.15151900 -0.37528200

C -1.27990600 -2.69246700 -0.13700900

C 0.59043800 2.60290800 -0.26645900

C 2.44251700 1.29359700 -0.19220000

C -2.83826100 -1.04658200 -0.25050600

C -2.57910000 -3.31479600 -0.27104900

C 1.72427200 3.45352800 -0.48969200

C 2.85841500 2.65849900 -0.44135200

C -3.52100100 -2.31899300 -0.33765900

O -0.12414900 -0.06164600 2.10070600

C -2.39750700 -0.34083500 3.19807300

H -2.64906400 -1.37545800 2.96820100

H -1.24671800 -0.18684800 2.64857600

H -3.03374100 0.39730300 2.71211700

H -2.19636000 -0.14500300 4.25034700

**RC (*F*_z1_=-0.007 a.u.)**

Fe -0.56145500 -0.34026600 0.24391000

H 3.00171100 -3.96601900 -0.18690800

H -2.88000600 4.03475800 -0.78784800

H 4.35252600 -1.74153000 0.56638600

H -4.62840300 2.17549300 -1.62696000

H -1.97780500 -4.81878700 -1.73837100

H 0.49062500 4.60932900 0.24046500

H 2.87295300 3.45290800 0.67971000

H -4.12397200 -3.19510300 -2.07231700

N 1.09876400 -1.24666600 0.02889700

N -1.87957500 0.89833500 -0.34830900

N -1.45589700 -1.68340900 -0.77097100

N 0.41149300 1.28760700 0.36564000

C 2.62615400 -2.96109300 -0.05461500

C -2.75494600 2.96111600 -0.75645800

C 3.30384800 -1.84591300 0.32526000

C -3.65378800 2.00869500 -1.18950200

C 1.22319400 -2.57786800 -0.26218500

C -1.60114600 2.26007900 -0.24022900

C 2.34774300 -0.72992900 0.36362300

C -3.06088100 0.69604800 -0.95252500

N 0.25775100 -3.39215700 -0.70598900

N 2.66540600 0.53617800 0.58799100

N -3.52802700 -0.52403600 -1.33176300

C -0.98407700 -2.95371400 -0.95211500

C -0.27382900 2.48494400 0.16901700

C 1.72545500 1.51075600 0.52705300

C -2.76761300 -1.60428100 -1.22990300

C -2.06826200 -3.76402600 -1.51912000

C 0.70002700 3.55032800 0.30042900

C 1.92254700 2.96198000 0.52347500

C -3.14514400 -2.94886800 -1.68464600

O -0.99673300 -0.66466600 1.76066600

C 0.83807900 1.73914100 3.76340900

H 1.89586500 1.54741700 3.56169300

H 0.22381400 0.97254600 3.28563500

H 0.67296300 1.71941300 4.84239500

H 0.56734400 2.72635500 3.37817600

**TS1 (*F*_z1_=-0.007 a.u.)**

Fe -0.14709400 -0.15168900 0.35095300

H 2.50365400 -4.42790200 -0.40744600

H -1.88686500 4.50264400 -0.47753100

H 4.36339700 -2.46100900 -0.26578700

H -4.08660200 2.96698100 -0.62546900

H -2.77106800 -4.39532900 -0.63614000

H 1.71924200 4.48579400 -0.32816800

H 3.90370000 2.92173400 -0.31779600

H -4.61607300 -2.40961200 -0.64504400

N 1.22362500 -1.37576800 -0.12660300

N -1.32638900 1.26254500 -0.05345000

N -1.48884600 -1.36079400 -0.21659800

N 1.08478900 1.25122000 0.00667700

C 2.34593100 -3.36118200 -0.32957000

C -1.93269700 3.42672200 -0.37855900

C 3.28651900 -2.36728400 -0.25096100

C -3.07059300 2.63554900 -0.46055600

C 1.03756100 -2.74199700 -0.26490200

C -0.81739900 2.55974200 -0.14386100

C 2.59379500 -1.09940600 -0.14082100

C -2.67584600 1.25731200 -0.28333300

N -0.13509900 -3.37148400 -0.35727200

N 3.17454900 0.10235900 -0.14609600

N -3.41450400 0.14092600 -0.38666700

C -1.30718000 -2.72937500 -0.34899400

C 0.60170700 2.54936100 -0.10177200

C 2.44423100 1.22666700 -0.10850200

C -2.85266200 -1.06834900 -0.35013700

C -2.60966900 -3.33156200 -0.53002000

C 1.74461300 3.40807100 -0.24313800

C 2.87278400 2.60410300 -0.24418700

C -3.54430100 -2.32642800 -0.52984600

O -0.10869800 -0.26274900 2.07349400

C -2.34774500 -0.02338600 3.25582500

H -2.51944300 1.05162200 3.28952600

H -1.22995400 -0.14217800 2.65711200

H -2.14916400 -0.47708100 4.22605000

H -3.05020700 -0.57479000 2.63159000

**RC (*F*_z1_=-0.008 a.u.)**

Fe -0.56943500 -0.33313600 0.24407300

H 3.03467900 -3.93422400 -0.00667500

H -2.92974000 3.98419700 -0.92445300

H 4.35018100 -1.67372800 0.69830600

H -4.64492000 2.08203500 -1.73609600

H -1.91567500 -4.88807400 -1.60455200

H 0.42586300 4.62690100 0.13769100

H 2.81345200 3.50931900 0.64284500

H -4.07319500 -3.29876000 -2.01937400

N 1.10280600 -1.22810300 0.08346000

N -1.89134800 0.87358600 -0.40414400

N -1.43533200 -1.71620700 -0.74193800

N 0.38374800 1.30818900 0.33315200

C 2.64781200 -2.92879800 0.08218900

C -2.78883200 2.91340600 -0.86980900

C 3.30793500 -1.79532200 0.43801900

C -3.67082900 1.93840300 -1.28918800

C 1.24492300 -2.56660100 -0.16270300

C -1.63162800 2.24091200 -0.32505800

C 2.34063300 -0.68815600 0.42362100

C -3.06386100 0.64012700 -1.01401300

N 0.29451200 -3.40374000 -0.59676000

N 2.64109900 0.58783900 0.61397000

N -3.51203800 -0.59645400 -1.36398800

C -0.94835300 -2.98668800 -0.87412900

C -0.31241300 2.49207600 0.09782500

C 1.69212300 1.54991900 0.51189800

C -2.74201900 -1.66522300 -1.21900100

C -2.01752100 -3.82652800 -1.42678400

C 0.64701100 3.57186700 0.22106100

C 1.87236400 3.00351300 0.47773300

C -3.10029100 -3.02819200 -1.63288600

O -1.02715800 -0.61492000 1.76219400

C 0.86628300 1.80718400 3.73476600

H 0.70690900 1.80134200 4.81467500

H 0.24036100 1.04183400 3.27002500

H 0.60371300 2.79312700 3.34051000

H 1.92090200 1.60139500 3.53029300

**TS1 (*F*_z1_=-0.008 a.u.)**

Fe -0.14953100 -0.14369100 0.34246900

H 2.55399400 -4.40870000 -0.26651700

H -1.94224000 4.46813800 -0.60077300

H 4.39003500 -2.41738000 -0.17085000

H -4.12332900 2.90285900 -0.71746600

H -2.72126700 -4.44374100 -0.52644700

H 1.66696800 4.49766400 -0.43197000

H 3.86916000 2.95956100 -0.37112600

H -4.58917100 -2.48101100 -0.59658200

N 1.23725000 -1.36408100 -0.09674300

N -1.34388700 1.24581600 -0.10342800

N -1.47524700 -1.38345200 -0.19479600

N 1.06710000 1.26390700 -0.03601900

C 2.38347100 -3.34178500 -0.22807400

C -1.97481600 3.39384700 -0.48065500

C 3.31210200 -2.33510600 -0.17275000

C -3.10327700 2.58711400 -0.54660300

C 1.06758500 -2.73592400 -0.19347600

C -0.84987000 2.54619800 -0.22332500

C 2.60423500 -1.07230300 -0.10778300

C -2.69259500 1.21849700 -0.33556600

N -0.09748600 -3.38120200 -0.27403700

N 3.17095500 0.13565000 -0.14298100

N -3.41760800 0.09067500 -0.41222500

C -1.27706400 -2.75299600 -0.28800700

C 0.56965000 2.55346700 -0.17573000

C 2.42758600 1.25227500 -0.13945600

C -2.84198300 -1.11056200 -0.34022900

C -2.57207700 -3.37530100 -0.45480800

C 1.70362200 3.42192500 -0.32769700

C 2.84123800 2.63112500 -0.30277000

C -3.51832400 -2.38147800 -0.48562400

O -0.10471000 -0.20731100 2.06552200

C -2.33766800 0.02141500 3.26897400

H -2.12008800 -0.40714400 4.24670900

H -1.23271600 -0.09195600 2.66246700

H -3.04045000 -0.55491900 2.66777900

H -2.52754700 1.09390900 3.28259100

**RC (*F*_z1_=-0.009 a.u.)**

Fe -0.57230100 -0.32234200 0.23912500

H 3.02237300 -3.93240600 0.00324200

H -2.93455400 4.00327600 -0.89248300

H 4.34011800 -1.67627100 0.71610400

H -4.65099000 2.10725000 -1.71537200

H -1.93183500 -4.87348000 -1.60465200

H 0.42707100 4.63756300 0.17781800

H 2.81185800 3.51371600 0.67979400

H -4.08530500 -3.27867400 -2.01881600

N 1.09821900 -1.22053400 0.08039900

N -1.89124600 0.88942900 -0.40644500

N -1.43998500 -1.70141400 -0.75179800

N 0.38273400 1.31697400 0.33698400

C 2.63832900 -2.92537200 0.08619600

C -2.79081200 2.93222900 -0.85136200

C 3.29982900 -1.79390500 0.44621600

C -3.67358000 1.95992400 -1.27686200

C 1.23749200 -2.55933500 -0.16589700

C -1.63177100 2.25615100 -0.31556400

C 2.33562200 -0.68393000 0.42765400

C -3.06560400 0.65976900 -1.01399500

N 0.28587800 -3.39315700 -0.60439500

N 2.63755800 0.59129800 0.62205200

N -3.51509900 -0.57550900 -1.36907100

C -0.95623100 -2.97312200 -0.88259300

C -0.31230400 2.50339700 0.11079400

C 1.69058900 1.55553100 0.52294900

C -2.74729600 -1.64660000 -1.22671400

C -2.02855600 -3.81041600 -1.43286800

C 0.64782300 3.58144800 0.24744700

C 1.87186500 3.00992900 0.50254300

C -3.10959000 -3.00899100 -1.63867000

O -1.03484300 -0.60803400 1.75456900

C 0.92221500 1.79635100 3.74580400

H 0.77712400 1.77531500 4.82744600

H 0.28738500 1.03921600 3.27932400

H 0.65681100 2.78882600 3.37011700

H 1.97368700 1.59073800 3.52520400

**TS1 (*F*_z1_=-0.009 a.u.)**

Fe -0.14517500 -0.09578600 0.33857600

H 2.55680400 -4.38071900 -0.10878400

H -1.94260300 4.48460900 -0.74398400

H 4.39293000 -2.38787500 -0.08894800

H -4.12339200 2.91530100 -0.81092700

H -2.72088100 -4.42269100 -0.35992400

H 1.66866900 4.51835300 -0.57752100

H 3.87090900 2.98207000 -0.47211400

H -4.58879900 -2.46371600 -0.49448700

N 1.24114500 -1.33065300 -0.06784900

N -1.34154300 1.27863800 -0.15842300

N -1.47228800 -1.35331700 -0.14624900

N 1.06997800 1.29828300 -0.07914500

C 2.38653400 -3.31310400 -0.11706500

C -1.97380600 3.41354900 -0.59729300

C 3.31505200 -2.30520700 -0.10065400

C -3.10223500 2.60469600 -0.63701200

C 1.07073900 -2.70554900 -0.10788700

C -0.84819000 2.57409200 -0.31541000

C 2.60701100 -1.04008800 -0.08551300

C -2.69006900 1.24360700 -0.38348700

N -0.09483600 -3.35257600 -0.16109200

N 3.17368800 0.16631600 -0.16041000

N -3.41544900 0.11269400 -0.41707000

C -1.27466800 -2.72486600 -0.19293100

C 0.57158300 2.58377700 -0.26245500

C 2.43012100 1.28259200 -0.18872000

C -2.84064600 -1.08458600 -0.29954800

C -2.57083300 -3.35229400 -0.33155900

C 1.70484900 3.44588300 -0.44339700

C 2.84289000 2.65546700 -0.39590200

C -3.51724300 -2.36000300 -0.39481900

O -0.10065900 -0.10183300 2.06052200

C -2.35021500 -0.18071900 3.26051500

H -2.71544000 -1.18619100 3.05342500

H -1.24326200 -0.13212900 2.66436600

H -2.94484500 0.61580300 2.81489800

H -2.07382500 -0.00782900 4.30027600

**RC (*F*_z1_=-0.010 a.u.)**

Fe -0.57790200 -0.32018300 0.24151700

H 3.05989800 -3.89220700 0.13924100

H -3.00025400 3.94425300 -0.98790600

H 4.34473000 -1.60171700 0.79841000

H -4.68708900 2.00573700 -1.77246300

H -1.87642600 -4.93902100 -1.46945600

H 0.35399200 4.65038400 0.08688300

H 2.74967600 3.56962800 0.62898300

H -4.04689100 -3.38338700 -1.93826600

N 1.10408800 -1.20197200 0.11922100

N -1.90785800 0.85800100 -0.44359300

N -1.42360000 -1.73670500 -0.71539000

N 0.35529200 1.33310500 0.30272200

C 2.66407100 -2.88738800 0.18602800

C -2.83786000 2.87664700 -0.92912800

C 3.30918700 -1.73839700 0.51929200

C -3.70568600 1.88193000 -1.33540700

C 1.26093600 -2.54540800 -0.08817200

C -1.66913500 2.22991600 -0.37926200

C 2.33217500 -0.64075200 0.46140200

C -3.07864300 0.59709900 -1.04528200

N 0.32209500 -3.40200100 -0.51049800

N 2.61644100 0.64321200 0.62330800

N -3.51009100 -0.65343900 -1.37049200

C -0.92392300 -3.00561600 -0.80631800

C -0.35479400 2.50485000 0.04908400

C 1.65810100 1.59262700 0.49562400

C -2.72986600 -1.71101300 -1.19587300

C -1.98454100 -3.87167800 -1.33475100

C 0.58938500 3.59867900 0.17350700

C 1.81920400 3.04930600 0.44909500

C -3.07430900 -3.08947900 -1.56804400

O -1.04834800 -0.56762900 1.76076900

C 0.97954100 1.85649100 3.71542800

H 0.71330400 2.84666400 3.33410600

H 0.33478600 1.09764500 3.26532800

H 2.02734000 1.64380200 3.48441300

H 0.84766900 1.84656800 4.79886300

**TS1 (*F*_z1_=-0.010 a.u.)**

Fe -0.15782500 -0.14379700 0.32130200

H 2.59519200 -4.39239700 -0.13391700

H -1.99985900 4.42896800 -0.70963400

H 4.40857800 -2.37976900 -0.07569800

H -4.16268700 2.83618700 -0.80445700

H -2.68088200 -4.49217100 -0.43352100

H 1.61359700 4.50334000 -0.51261200

H 3.83163300 2.99037300 -0.40747400

H -4.56978000 -2.55282900 -0.55893000

N 1.24412800 -1.35900400 -0.08230300

N -1.36609100 1.22295600 -0.15698300

N -1.46821400 -1.41065000 -0.18877800

N 1.04486200 1.26839100 -0.08774900

C 2.41287900 -3.32670300 -0.13959400

C -2.01921900 3.35568100 -0.57761600

C 3.33015200 -2.30873200 -0.10311900

C -3.13854100 2.53436700 -0.63245300

C 1.08988800 -2.73425500 -0.13957600

C -0.88569800 2.52629600 -0.30090100

C 2.60816700 -1.05204500 -0.08517400

C -2.71305300 1.17503800 -0.39354200

N -0.06795800 -3.39413500 -0.21201900

N 3.16153500 0.16087100 -0.14680200

N -3.42524100 0.03691200 -0.44869000

C -1.25441400 -2.77958400 -0.24615300

C 0.53419900 2.54988100 -0.24903400

C 2.40597500 1.26972900 -0.17462500

C -2.83721500 -1.15595800 -0.34429200

C -2.54262800 -3.42038300 -0.39870100

C 1.66023700 3.42853500 -0.40223300

C 2.80645600 2.65052300 -0.35441300

C -3.49945900 -2.43803500 -0.45759600

O -0.09370100 -0.16850700 2.04002500

C -2.29008200 0.10299800 3.32078700

H -3.04660400 -0.39643300 2.71535600

H -1.22613700 -0.02931200 2.68112500

H -2.42055600 1.18131100 3.40645600

H -2.06963400 -0.39366000 4.26553900

**P (*F*_z1_=-0.010 a.u.)**

Fe -0.33503600 0.01692800 0.14811400

H 2.18414200 -4.36291200 0.48660000

H -1.72865600 4.60636100 -1.22124600

H 4.07344500 -2.43977400 0.78667900

H -3.92553200 3.12025400 -1.70316200

H -2.91493900 -4.24242000 -0.76736200

H 1.73722700 4.52747700 -0.34057200

H 3.83341700 2.93590200 0.22577600

H -4.64902500 -2.23362400 -1.34849000

N 1.02417700 -1.27109600 0.17334300

N -1.39701000 1.39753300 -0.50718700

N -1.61789900 -1.20644500 -0.46753500

N 0.96823100 1.33659400 0.04125600

C 2.06728400 -3.29093200 0.40886500

C -1.83342600 3.53893200 -1.08059300

C 3.02259900 -2.31838200 0.56297800

C -2.97079600 2.77183800 -1.33281300

C 0.80634000 -2.63201500 0.14283600

C -0.82900200 2.65758900 -0.57739200

C 2.37970000 -1.03119600 0.39910400

C -2.67061800 1.40627000 -0.99261600

N -0.35001200 -3.24601100 -0.11868700

N 3.00212800 0.14578800 0.41950100

N -3.42181900 0.30045500 -1.14882000

C -1.46651100 -2.57611800 -0.41260800

C 0.55427500 2.62301200 -0.24657000

C 2.31843600 1.28483200 0.21867500

C -2.91058200 -0.90394800 -0.88945400

C -2.73304600 -3.17652200 -0.76979500

C 1.71570000 3.45549500 -0.19855900

C 2.80317300 2.63524800 0.09331900

C -3.60907000 -2.16022700 -1.06105400

O -0.74456900 0.04550700 2.23514000

C 0.29259100 0.24463900 3.23825400

H 1.14583400 -0.35122300 2.91841000

H -1.58586700 0.40618400 2.54746600

H -0.06383100 -0.11221300 4.20568200

H 0.56726300 1.30049000 3.29065100

**Coordinates of the quartet state under the *F*_z2_**

**RC (*F*_z2_=+0.001 a.u.)**

O -0.46334100 1.89004600 0.63681100

C 1.68936000 2.36664600 3.30643400

Fe -0.32837600 0.38013600 0.07826400

H 4.59176800 0.48567900 -1.29626500

H -5.02835600 -1.47435000 -0.14661300

H 3.22210200 1.13202700 -3.54533700

H -4.15831700 -2.24516700 2.27613400

H 3.14135900 -1.40851800 3.37620100

H -4.05230700 -0.22905400 -3.29075500

H -1.96519300 0.55691600 -4.78824000

H 0.74312100 -2.08171200 4.45025600

N 1.28524200 0.38903400 -0.93233800

N -1.95253900 -0.45871600 0.60481300

N 0.53705200 -0.56907800 1.48519400

N -1.27847000 0.40795200 -1.56829900

C 3.52264300 0.54358900 -1.44412800

C -4.07032100 -1.25381600 0.30351000

C 2.83635700 0.86851100 -2.57049400

C -3.62755000 -1.65062400 1.54574000

C 2.53741600 0.22012300 -0.40560100

C -2.99477200 -0.51377600 -0.31815700

C 1.40265100 0.75301000 -2.27120500

C -2.25849000 -1.16918700 1.70329000

N 2.83743600 -0.22184700 0.81964200

N 0.43452700 0.87796200 -3.16420400

N -1.38308400 -1.45614700 2.69957300

C 1.89111200 -0.61744700 1.67688700

C -2.60550800 -0.01581300 -1.57142300

C -0.85337600 0.62967800 -2.82368800

C -0.09666100 -1.17672500 2.56596100

C 2.15030100 -1.24368000 2.97774100

C -3.06240500 0.03332100 -2.94407300

C -1.99583500 0.43513300 -3.71451800

C 0.94869700 -1.57774400 3.51628700

H 2.69058900 2.14170400 2.92890200

H 0.96464000 2.28507900 2.49268800

H 1.43525600 1.65728900 4.09891000

H 1.67452200 3.38241000 3.71069100

**TS1 (*F*_z2_=+0.001 a.u.)**

O -0.01004400 -0.01840100 0.12786500

C 0.06990300 0.22217800 2.65839400

Fe -0.07839200 -1.63577100 -0.46391600

H 4.67829500 -1.87093400 -2.28303300

H -4.93928500 -2.95498500 -0.13144700

H 3.14721800 -1.16968200 -4.40589100

H -3.90489600 -3.75233300 2.21627100

H 3.53625900 -3.53318500 2.57596200

H -4.16366000 -1.85450300 -3.42679000

H -2.17405500 -1.27510100 -5.13565300

H 1.20710200 -4.01301100 3.87801900

N 1.41956500 -1.76113300 -1.62484100

N -1.69601500 -2.34451300 0.21829000

N 0.83646900 -2.59888000 0.88457700

N -1.17842200 -1.53882900 -2.00457400

C 3.60460700 -1.75357700 -2.32657200

C -3.91469400 -2.84545900 0.19624800

C 2.83283000 -1.39381900 -3.39620100

C -3.38349100 -3.26065000 1.40671500

C 2.71874900 -1.99927400 -1.20244200

C -2.84532000 -2.27710800 -0.57192400

C 1.44249200 -1.40822700 -2.97334200

C -1.96739200 -2.96533100 1.40025400

N 3.08935800 -2.42220200 0.00368300

N 0.39501700 -1.21915700 -3.77542400

N -1.02976800 -3.29488300 2.30896300

C 2.20840700 -2.72783700 0.96459900

C -2.54040300 -1.81632100 -1.87333600

C -0.85775200 -1.34833900 -3.31429200

C 0.26348200 -3.12746300 2.05514300

C 2.52889000 -3.31318100 2.25092300

C -3.12192000 -1.70014700 -3.18201100

C -2.09908900 -1.40954000 -4.06545700

C 1.35194700 -3.55097500 2.91158700

H 1.01077900 -0.27081900 2.89781200

H 0.01839900 0.10336300 1.29822200

H -0.81881000 -0.31222600 2.98936800

H 0.05371900 1.29698800 2.83024800

**RC (*F*_z2_=+0.002 a.u.)**

O -0.48433500 1.87570400 0.67316800

C 1.75411900 2.39249800 3.24966100

Fe -0.33955800 0.37428400 0.09359600

H 4.58672400 0.49917500 -1.26352100

H -5.02697300 -1.50487000 -0.17219900

H 3.22332100 1.15767300 -3.51258300

H -4.16659300 -2.28906200 2.24988500

H 3.12325700 -1.42202100 3.39302100

H -4.04219700 -0.23334600 -3.30003400

H -1.95181800 0.57294300 -4.78094600

H 0.72260900 -2.11245600 4.45263500

N 1.27862900 0.39843900 -0.90920900

N -1.96210500 -0.47716400 0.60544500

N 0.52382700 -0.58382900 1.49634500

N -1.28187800 0.41211800 -1.55659000

C 3.51797100 0.55828100 -1.41341600

C -4.07345600 -1.28109400 0.28588700

C 2.83525400 0.88928300 -2.53997300

C -3.63518300 -1.68462400 1.52811400

C 2.52977200 0.22746800 -0.38000000

C -2.99923500 -0.53037400 -0.32362100

C 1.40073000 0.77006100 -2.24568600

C -2.27051300 -1.19702300 1.69696800

N 2.82608400 -0.22177300 0.84344600

N 0.43603700 0.89718100 -3.14187300

N -1.39853900 -1.48565100 2.69679100

C 1.87710600 -0.62652500 1.69367500

C -2.60608400 -0.02060100 -1.57058400

C -0.85141100 0.64209000 -2.80898400

C -0.11263100 -1.19985800 2.57043200

C 2.13302000 -1.25824600 2.99197400

C -3.05614400 0.03370900 -2.94615400

C -1.98846000 0.44573800 -3.70802200

C 0.92997800 -1.60083500 3.52317800

H 2.74271200 2.18142500 2.83314400

H 0.99998500 2.29996500 2.46418100

H 1.54070500 1.67881200 4.05048600

H 1.74011400 3.40822700 3.65448200

**TS1 (*F*_z2_=+0.002 a.u.)**

O -0.01363200 -0.01957400 0.14086800

C 0.08996200 0.21916500 2.67483100

Fe -0.08237200 -1.63031900 -0.46446500

H 4.67649900 -1.86529900 -2.28103400

H -4.94034400 -2.96546600 -0.14468500

H 3.14627200 -1.16662000 -4.40458000

H -3.90936200 -3.76113300 2.20535600

H 3.53076000 -3.52941700 2.57593000

H -4.16188800 -1.86716300 -3.43614100

H -2.17057200 -1.28691200 -5.14168600

H 1.20039100 -4.01317600 3.87638700

N 1.41655500 -1.75486400 -1.62322200

N -1.70047900 -2.34255700 0.21549100

N 0.83207500 -2.59386700 0.88546000

N -1.18092700 -1.53216200 -2.00605500

C 3.60292500 -1.74713500 -2.32443500

C -3.91747500 -2.85140800 0.18685500

C 2.83212200 -1.38885300 -3.39438000

C -3.38777500 -3.26530000 1.39842300

C 2.71593700 -1.99232900 -1.19991100

C -2.84803700 -2.27835200 -0.57779800

C 1.44079200 -1.40368600 -2.97165600

C -1.97271300 -2.96489000 1.39562600

N 3.08562900 -2.41428400 0.00615100

N 0.39442700 -1.21715400 -3.77516000

N -1.03554400 -3.29345200 2.30660300

C 2.20282400 -2.72155300 0.96618100

C -2.54217000 -1.81621500 -1.87796900

C -0.85827300 -1.34746000 -3.31556800

C 0.25772200 -3.12443900 2.05434700

C 2.52321800 -3.30862700 2.25183300

C -3.12159000 -1.70709900 -3.18908200

C -2.09844600 -1.41634900 -4.07066200

C 1.34584200 -3.54828100 2.91138500

H 1.03046800 -0.27935200 2.90392200

H 0.02575600 0.09836100 1.30148900

H -0.79894700 -0.31203700 3.01038900

H 0.08045200 1.29399100 2.84696200

**RC (*F*_z2_=+0.003 a.u.)**

O -0.49822500 1.86391000 0.70283300

C 1.79472600 2.40874100 3.21546400

Fe -0.34572000 0.37004100 0.10502600

H 4.58365300 0.51621200 -1.24356700

H -5.02102900 -1.53303400 -0.19101500

H 3.22196800 1.18561700 -3.49013100

H -4.16561000 -2.32895500 2.22922400

H 3.11682100 -1.43564600 3.39844400

H -4.03409200 -0.23736200 -3.30503400

H -1.94363100 0.58829400 -4.77409100

H 0.71595200 -2.14215200 4.44892300

N 1.27473600 0.40713900 -0.89343600

N -1.96584100 -0.49283700 0.60631400

N 0.51828000 -0.59565300 1.50287500

N -1.28371100 0.41675400 -1.54673400

C 3.51494500 0.57532200 -1.39363500

C -4.07124900 -1.30574200 0.27310300

C 2.83350000 0.91175400 -2.51916900

C -3.63508000 -1.71513500 1.51473100

C 2.52592700 0.23597700 -0.36372100

C -2.99963300 -0.54486500 -0.32672400

C 1.39881400 0.78728200 -2.22748700

C -2.27447000 -1.22154000 1.69184200

N 2.82105100 -0.21988600 0.85749800

N 0.43577300 0.91644400 -3.12493600

N -1.40387900 -1.51191900 2.69331700

C 1.87116500 -0.63373900 1.70265400

C -2.60504000 -0.02450700 -1.56865500

C -0.85047600 0.65441300 -2.79709000

C -0.11867500 -1.22024800 2.57144700

C 2.12655800 -1.27187600 2.99732400

C -3.05116100 0.03434200 -2.94613500

C -1.98396100 0.45606100 -3.70186400

C 0.92316000 -1.62245700 3.52384100

H 2.77459700 2.20874400 2.77388500

H 1.02270200 2.30909700 2.44836700

H 1.60858200 1.69042000 4.01919000

H 1.77961800 3.42374900 3.62250200

**TS1 (*F*_z2_=+0.003 a.u.)**

O -0.01421300 -0.02058400 0.15270100

C 0.11704400 0.21750200 2.69051500

Fe -0.08672400 -1.62484300 -0.46541000

H 4.67302300 -1.86509800 -2.28212500

H -4.94257400 -2.97195800 -0.15450600

H 3.14287700 -1.16980000 -4.40613100

H -3.91427500 -3.76501700 2.19790100

H 3.52511600 -3.52490600 2.57543100

H -4.16246600 -1.87942000 -3.44319600

H -2.17000400 -1.30163700 -5.14729600

H 1.19386800 -4.00989000 3.87593800

N 1.41223200 -1.75142100 -1.62303200

N -1.70566900 -2.33827000 0.21400000

N 0.82716000 -2.58881300 0.88602000

N -1.18474400 -1.52581100 -2.00742700

C 3.59963400 -1.74535000 -2.32484700

C -3.92123400 -2.85374700 0.18029700

C 2.82943400 -1.38909000 -3.39502700

C -3.39266100 -3.26588400 1.39298100

C 2.71191600 -1.98856500 -1.19927300

C -2.85176400 -2.27719900 -0.58175700

C 1.43731200 -1.40299500 -2.97160000

C -1.97849400 -2.96127000 1.39284200

N 3.08106600 -2.40843500 0.00699400

N 0.39171700 -1.21876000 -3.77609600

N -1.04150200 -3.28880500 2.30579800

C 2.19670400 -2.71576100 0.96695900

C -2.54526200 -1.81529700 -1.88143300

C -0.86070100 -1.34860700 -3.31714700

C 0.25176900 -3.11950700 2.05414100

C 2.51737500 -3.30314300 2.25249300

C -3.12337500 -1.71409700 -3.19469600

C -2.10021000 -1.42505000 -4.07536400

C 1.33972200 -3.54333200 2.91174500

H 1.05424800 -0.29168100 2.90927200

H 0.03844000 0.09405100 1.30335800

H -0.77496800 -0.30525000 3.03106600

H 0.12026500 1.29247300 2.86190900

**RC (*F*_z2_=+0.004 a.u.)**

O -0.49909000 1.85726700 0.72035900

C 1.83136900 2.42459300 3.18209000

Fe -0.34736000 0.36704600 0.11239800

H 4.58458000 0.49827400 -1.23328100

H -5.02205600 -1.53376300 -0.19701200

H 3.22626100 1.17203900 -3.48030800

H -4.17381300 -2.33127500 2.22547800

H 3.10785600 -1.44585900 3.40825800

H -4.02850400 -0.23898000 -3.30659100

H -1.93466200 0.58476500 -4.77099900

H 0.70378900 -2.14867600 4.45611100

N 1.27459100 0.40248200 -0.88335200

N -1.96921500 -0.49534700 0.60977900

N 0.51316800 -0.60256200 1.51042600

N -1.28233500 0.41779700 -1.54031500

C 3.51612300 0.56255400 -1.38286500

C -4.07407000 -1.30602400 0.27058400

C 2.83679600 0.90114000 -2.50885500

C -3.64116700 -1.71602800 1.51374800

C 2.52535600 0.22707300 -0.35329400

C -3.00101500 -0.54561200 -0.32563700

C 1.40150500 0.78166100 -2.21756800

C -2.28134700 -1.22377400 1.69447400

N 2.81785900 -0.22936900 0.86829500

N 0.43998000 0.91266800 -3.11616700

N -1.41286700 -1.51454100 2.69857400

C 1.86562000 -0.64186300 1.71184800

C -2.60334800 -0.02434300 -1.56616300

C -0.84627000 0.65319000 -2.79052800

C -0.12675500 -1.22512500 2.57809600

C 2.11812100 -1.27942300 3.00678300

C -3.04668900 0.03355800 -2.94534700

C -1.97827400 0.45409200 -3.69863800

C 0.91289300 -1.62807500 3.53186000

H 2.80358200 2.22346700 2.72454700

H 1.04679100 2.31895300 2.42854200

H 1.66006900 1.70960700 3.99230900

H 1.82133500 3.44211800 3.58338500

**TS1 (*F*_z2_=+0.004 a.u.)**

O -0.01518200 -0.02083700 0.16408300

C 0.14176600 0.21531200 2.70630200

Fe -0.09077100 -1.61904700 -0.46648500

H 4.67051400 -1.86627700 -2.28155800

H -4.94460700 -2.97750700 -0.16576000

H 3.14136700 -1.17546700 -4.40710600

H -3.92008900 -3.76681200 2.18992300

H 3.51851700 -3.51906900 2.57687700

H -4.16154300 -1.89264300 -3.45184500

H -2.16726300 -1.31844400 -5.15421400

H 1.18585600 -4.00436500 3.87690300

N 1.40863800 -1.74807500 -1.62243100

N -1.71081700 -2.33338900 0.21193200

N 0.82192100 -2.58287900 0.88719400

N -1.18761700 -1.51995800 -2.00960400

C 3.59733100 -1.74488300 -2.32415600

C -3.92491900 -2.85519500 0.17262700

C 2.82821000 -1.39109000 -3.39506700

C -3.39807200 -3.26490900 1.38693800

C 2.70843700 -1.98508600 -1.19766900

C -2.85504800 -2.27569800 -0.58665900

C 1.43512200 -1.40351500 -2.97136000

C -1.98477400 -2.95633200 1.38980300

N 3.07654100 -2.40242700 0.00912000

N 0.39062300 -1.22209300 -3.77743900

N -1.04834500 -3.28253000 2.30518500

C 2.19026800 -2.70925000 0.96886600

C -2.54734200 -1.81476700 -1.88608800

C -0.86165400 -1.35097200 -3.31952200

C 0.24508000 -3.11313500 2.05451300

C 2.51070400 -3.29640500 2.25463000

C -3.12369100 -1.72221700 -3.20172700

C -2.10021800 -1.43531500 -4.08130700

C 1.33249700 -3.53663300 2.91332300

H 1.07534100 -0.30457400 2.91493800

H 0.04908900 0.08984600 1.30483800

H -0.75349000 -0.29894100 3.05122700

H 0.15770000 1.29031000 2.87677200

**RC (*F*_z2_=+0.005 a.u.)**

O -0.49977100 1.85009500 0.73886800

C 1.85201300 2.43323300 3.16555300

Fe -0.34775700 0.36460200 0.11849000

H 4.58387900 0.49638500 -1.23247500

H -5.01769600 -1.54488500 -0.19926200

H 3.22331000 1.17436500 -3.47657400

H -4.17034700 -2.34612400 2.22259700

H 3.11028100 -1.45366000 3.40719000

H -4.02637600 -0.24381600 -3.30381200

H -1.93379300 0.58524900 -4.76613800

H 0.70665200 -2.16049800 4.45538300

N 1.27368800 0.40316700 -0.87759500

N -1.96881400 -0.50108900 0.61378200

N 0.51397300 -0.60888100 1.51371300

N -1.28296300 0.41996600 -1.53344900

C 3.51524900 0.56289300 -1.37971400

C -4.07139100 -1.31489700 0.27067400

C 2.83521200 0.90354100 -2.50448000

C -3.63854400 -1.72659600 1.51389700

C 2.52538700 0.22647100 -0.34945800

C -2.99997900 -0.55094000 -0.32234700

C 1.40014200 0.78430500 -2.21146100

C -2.28053600 -1.23234700 1.69680200

N 2.81854200 -0.23165100 0.87127400

N 0.43825000 0.91631500 -3.10929600

N -1.41164300 -1.52344200 2.70124900

C 1.86647100 -0.64679100 1.71417500

C -2.60261600 -0.02618900 -1.56157300

C -0.84680700 0.65578300 -2.78400400

C -0.12546800 -1.23311100 2.58041200

C 2.11997000 -1.28589300 3.00751800

C -3.04572000 0.03145500 -2.94153100

C -1.97842500 0.45450600 -3.69373400

C 0.91465900 -1.63646600 3.53270300

H 2.81847500 2.23454100 2.69508500

H 1.05815800 2.32258100 2.42237300

H 1.69417300 1.71860600 3.97904300

H 1.84344300 3.45192300 3.56437200

**TS1 (*F*_z2_=+0.005 a.u.)**

O -0.01695200 -0.02064000 0.17576500

C 0.16517500 0.21229400 2.72262900

Fe -0.09499700 -1.61319200 -0.46750700

H 4.66733100 -1.86361300 -2.28271600

H -4.94564800 -2.98626900 -0.17534000

H 3.13790000 -1.17807800 -4.40919500

H -3.92358100 -3.77150800 2.18318800

H 3.51425400 -3.51081800 2.57678300

H -4.16165600 -1.90806000 -3.45885200

H -2.16663300 -1.33569900 -5.16027900

H 1.18097500 -3.99845900 3.87706700

N 1.40447900 -1.74355300 -1.62245400

N -1.71538900 -2.32964600 0.21039700

N 0.81761100 -2.57663100 0.88782400

N -1.19129300 -1.51467800 -2.01138000

C 3.59424500 -1.74141200 -2.32480900

C -3.92760300 -2.85923800 0.16628400

C 2.82554700 -1.39049900 -3.39615200

C -3.40180100 -3.26636700 1.38201700

C 2.70476300 -1.97947100 -1.19718400

C -2.85800100 -2.27602900 -0.59050800

C 1.43167300 -1.40258100 -2.97166200

C -1.98961400 -2.95294900 1.38738000

N 3.07254900 -2.39414600 0.00996500

N 0.38782400 -1.22456900 -3.77894300

N -1.05320100 -3.27734100 2.30470200

C 2.18497400 -2.70127700 0.96977000

C -2.54984600 -1.81573500 -1.88989600

C -0.86399600 -1.35333400 -3.32164500

C 0.24022200 -3.10694200 2.05448200

C 2.50608300 -3.28802900 2.25556700

C -3.12505200 -1.73195000 -3.20757200

C -2.10182900 -1.44626900 -4.08647300

C 1.32769300 -3.52934500 2.91407600

H 1.09689700 -0.31480700 2.92080800

H 0.05872000 0.08518900 1.30650700

H -0.73143100 -0.29691500 3.07141800

H 0.18968900 1.28729800 2.89194000

**RC (*F*_z2_=+0.006 a.u.)**

O -0.50481100 1.84202500 0.75538900

C 1.85137000 2.43992800 3.16329500

Fe -0.34763400 0.36194800 0.12242000

H 4.58626900 0.50677000 -1.22297000

H -5.00751000 -1.56707600 -0.21415200

H 3.22671600 1.18656300 -3.46689100

H -4.16463400 -2.37018000 2.20896000

H 3.11047500 -1.45420600 3.41101600

H -4.01473200 -0.25672800 -3.31136200

H -1.92233300 0.58344400 -4.76685300

H 0.70666100 -2.17089600 4.45430000

N 1.27535000 0.40795300 -0.87045100

N -1.96662900 -0.51148200 0.61171000

N 0.51462100 -0.61389800 1.51640700

N -1.27955000 0.42005300 -1.53078500

C 3.51763200 0.57297200 -1.37018300

C -4.06443500 -1.33283800 0.26020100

C 2.83852800 0.91447300 -2.49509600

C -3.63350000 -1.74525200 1.50444200

C 2.52724000 0.23284100 -0.34152900

C -2.99501900 -0.56307000 -0.32728500

C 1.40332900 0.79186000 -2.20353800

C -2.27873200 -1.24610500 1.69248400

N 2.81951400 -0.22660100 0.87886100

N 0.44273200 0.92286500 -3.10268600

N -1.41102100 -1.53555100 2.69916100

C 1.86681900 -0.64704800 1.71892400

C -2.59662400 -0.03337100 -1.56424000

C -0.84121000 0.65794200 -2.78067200

C -0.12512800 -1.24131100 2.58074500

C 2.12004500 -1.28736800 3.01105400

C -3.03670300 0.02353300 -2.94583200

C -1.96994000 0.45204000 -3.69453800

C 0.91432600 -1.64278000 3.53372800

H 2.81591900 2.24509500 2.68742100

H 1.05419000 2.32274800 2.42451700

H 1.70190700 1.72575400 3.97899200

H 1.83947000 3.45990600 3.55919500

**TS1 (*F*_z2_=+0.006 a.u.)**

O -0.01859300 -0.02017100 0.18844500

C 0.18545900 0.20702800 2.74037900

Fe -0.09884500 -1.60718000 -0.46837500

H 4.66517100 -1.86023900 -2.28215500

H -4.94637700 -2.99447300 -0.18698300

H 3.13624400 -1.17900100 -4.40988000

H -3.92776300 -3.77661000 2.17453700

H 3.50924000 -3.50382100 2.57725800

H -4.16024700 -1.92155700 -3.46719400

H -2.16389300 -1.35024700 -5.16683000

H 1.17482100 -3.99422600 3.87679500

N 1.40108900 -1.73847700 -1.62193600

N -1.71981500 -2.32599500 0.20806900

N 0.81308400 -2.57099600 0.88843700

N -1.19396000 -1.50882700 -2.01344700

C 3.59219900 -1.73718300 -2.32408200

C -3.93010800 -2.86301300 0.15828100

C 2.82431300 -1.38867200 -3.39603500

C -3.40587300 -3.26804400 1.37553000

C 2.70173700 -1.97354100 -1.19580500

C -2.86044100 -2.27603600 -0.59542200

C 1.42954400 -1.40052900 -2.97126700

C -1.99478800 -2.95004500 1.38398000

N 3.06872600 -2.38612700 0.01167600

N 0.38661900 -1.22552200 -3.78007700

N -1.05879100 -3.27307900 2.30335200

C 2.17955600 -2.69394100 0.97109900

C -2.55129800 -1.81596500 -1.89463600

C -0.86485700 -1.35424900 -3.32394900

C 0.23480900 -3.10172300 2.05401500

C 2.50084000 -3.28074000 2.25677000

C -3.12494500 -1.74011600 -3.21438500

C -2.10166500 -1.45519800 -4.09219000

C 1.32196200 -3.52338100 2.91459700

H 1.11543700 -0.32660600 2.92843700

H 0.06672200 0.07949300 1.30915300

H -0.71254800 -0.29822300 3.09113100

H 0.21731800 1.28196100 2.90871000

**RC (*F*_z2_=+0.007 a.u.)**

O -0.49871400 1.83837800 0.76628200

C 1.86180200 2.44249900 3.15772300

Fe -0.34526500 0.36111500 0.12479800

H 4.58681000 0.49258400 -1.23237200

H -5.00555800 -1.56578400 -0.20843600

H 3.22371900 1.17317300 -3.47365800

H -4.16244000 -2.36790500 2.21529000

H 3.11587500 -1.45940800 3.40654200

H -4.01601100 -0.25994100 -3.30566800

H -1.92476800 0.57658600 -4.76430200

H 0.71221300 -2.17245700 4.45506100

N 1.27593900 0.40436800 -0.87055600

N -1.96471500 -0.51081400 0.61637400

N 0.51771200 -0.61680600 1.51757600

N -1.27944300 0.42147200 -1.52660700

C 3.51797500 0.56277600 -1.37614400

C -4.06290100 -1.33056500 0.26630900

C 2.83752200 0.90462300 -2.49997600

C -3.63147900 -1.74219100 1.51125200

C 2.52901400 0.22672300 -0.34461400

C -2.99385100 -0.56187400 -0.32164100

C 1.40262400 0.78613400 -2.20458900

C -2.27683000 -1.24423800 1.69814900

N 2.82246800 -0.23173700 0.87582300

N 0.44082900 0.91772700 -3.10211500

N -1.40810600 -1.53346500 2.70470800

C 1.87017600 -0.65085100 1.71751900

C -2.59623900 -0.03278000 -1.55941500

C -0.84214700 0.65558700 -2.77811700

C -0.12149500 -1.24168000 2.58341100

C 2.12469600 -1.29011200 3.00926900

C -3.03790800 0.02143900 -2.94118600

C -1.97218400 0.44788400 -3.69150600

C 0.91875300 -1.64357300 3.53450600

H 2.82325300 2.24568500 2.67649100

H 1.06061200 2.32230100 2.42359000

H 1.71700800 1.73057800 3.97644400

H 1.85229300 3.46428200 3.54949500

**TS1 (*F*_z2_=+0.007 a.u.)**

O -0.01917500 -0.01948000 0.20131900

C 0.20508200 0.20023000 2.75876900

Fe -0.10215600 -1.60133500 -0.46940300

H 4.66270900 -1.86029500 -2.28375300

H -4.94735100 -2.99882500 -0.19632100

H 3.13386100 -1.18244900 -4.41218400

H -3.93185700 -3.77803900 2.16802300

H 3.50494800 -3.49801600 2.57656300

H -4.15961800 -1.93211300 -3.47398400

H -2.16230600 -1.36283200 -5.17269600

H 1.16960000 -3.98980300 3.87630200

N 1.39778600 -1.73409400 -1.62215500

N -1.72369000 -2.32183600 0.20606100

N 0.80902500 -2.56621000 0.88842000

N -1.19650300 -1.50288000 -2.01533700

C 3.58992600 -1.73556900 -2.32505600

C -3.93257600 -2.86386300 0.15202400

C 2.82263800 -1.38899400 -3.39736000

C -3.40974100 -3.26687900 1.37073000

C 2.69882600 -1.96935200 -1.19573800

C -2.86262300 -2.27460600 -0.59934900

C 1.42718700 -1.39948400 -2.97168600

C -1.99945100 -2.94582100 1.38142300

N 3.06520800 -2.37997300 0.01199100

N 0.38493800 -1.22689800 -3.78165100

N -1.06375100 -3.26785900 2.30248500

C 2.17469100 -2.68785700 0.97142600

C -2.55270600 -1.81498600 -1.89867100

C -0.86603700 -1.35477800 -3.32625600

C 0.23010800 -3.09654400 2.05335400

C 2.49631600 -3.27431100 2.25703300

C -3.12536000 -1.74625600 -3.22012100

C -2.10220800 -1.46259700 -4.09729800

C 1.31700700 -3.51758300 2.91468100

H 1.13080200 -0.34376000 2.93712100

H 0.07492400 0.07293600 1.31192000

H -0.69685100 -0.29727000 3.11024400

H 0.24846200 1.27501300 2.92513000

**RC (*F*_z2_=+0.008 a.u.)**

O -0.49406800 1.83355300 0.77874500

C 1.85769100 2.43535000 3.16932200

Fe -0.34187600 0.36048000 0.12630800

H 4.58995700 0.48766100 -1.23548900

H -5.00010800 -1.56854800 -0.21269600

H 3.22552600 1.17100700 -3.47485400

H -4.15898300 -2.37287300 2.21134900

H 3.11992800 -1.46662800 3.40264800

H -4.01119500 -0.26110100 -3.30662200

H -1.92020100 0.57663500 -4.76435900

H 0.71585600 -2.18001700 4.45218700

N 1.27875900 0.40351300 -0.86954500

N -1.96147800 -0.51230400 0.61666800

N 0.52107700 -0.62059200 1.51762100

N -1.27621300 0.42481600 -1.52446700

C 3.52102800 0.56031800 -1.37723400

C -4.05872800 -1.33213000 0.26403200

C 2.84031800 0.90346100 -2.50037600

C -3.62799300 -1.74456100 1.50955200

C 2.53256800 0.22462600 -0.34493900

C -2.99009400 -0.56243300 -0.32175100

C 1.40549600 0.78627700 -2.20347100

C -2.27423500 -1.24660800 1.69791100

N 2.82611100 -0.23435200 0.87518900

N 0.44355800 0.91915600 -3.10043800

N -1.40557100 -1.53627400 2.70501600

C 1.87356300 -0.65465900 1.71663600

C -2.59220100 -0.03161500 -1.55909600

C -0.83858700 0.65773600 -2.77664100

C -0.11835000 -1.24536300 2.58313000

C 2.12837300 -1.29493400 3.00711600

C -3.03377900 0.02182000 -2.94145100

C -1.96858100 0.44867000 -3.69134900

C 0.92183200 -1.64842800 3.53287200

H 2.81922200 2.24008900 2.68766500

H 1.05703300 2.31315300 2.43473600

H 1.71407000 1.72275300 3.98787300

H 1.84621700 3.45764700 3.56023800

**TS1 (*F*_z2_=+0.008 a.u.)**

O -0.01889700 -0.01805000 0.21323800

C 0.22280500 0.19487600 2.77709900

Fe -0.10520400 -1.59542600 -0.47042400

H 4.66057500 -1.86106600 -2.28490100

H -4.94783000 -3.00372900 -0.20609900

H 3.13201400 -1.18700800 -4.41438700

H -3.93569900 -3.77985700 2.16127000

H 3.50060000 -3.49191300 2.57645700

H -4.15849100 -1.94320800 -3.48080700

H -2.16014800 -1.37631700 -5.17874200

H 1.16416200 -3.98463000 3.87639200

N 1.39475500 -1.73034500 -1.62231100

N -1.72753500 -2.31680800 0.20442800

N 0.80498800 -2.56109200 0.88876400

N -1.19862300 -1.49750600 -2.01735400

C 3.58798300 -1.73470700 -2.32573300

C -3.93469900 -2.86494500 0.14556600

C 2.82139200 -1.39029800 -3.39858300

C -3.41340600 -3.26584700 1.36582500

C 2.69614800 -1.96565400 -1.19546200

C -2.86454400 -2.27289900 -0.60315100

C 1.42525900 -1.39931900 -2.97213300

C -2.00405000 -2.94118600 1.37908600

N 3.06182100 -2.37403400 0.01269400

N 0.38375000 -1.22924100 -3.78337000

N -1.06877900 -3.26222400 2.30188200

C 2.16993400 -2.68162300 0.97215200

C -2.55363100 -1.81441200 -1.90285400

C -0.86673500 -1.35601700 -3.32878200

C 0.22541000 -3.09085100 2.05305600

C 2.49176800 -3.26755500 2.25778400

C -3.12526000 -1.75302900 -3.22594100

C -2.10219500 -1.47076300 -4.10257700

C 1.31193700 -3.51129000 2.91521900

H 1.14911300 -0.35093700 2.94553000

H 0.08255100 0.06717300 1.31383700

H -0.67872000 -0.30340500 3.12812700

H 0.26756400 1.26987400 2.94120500

**RC (*F*_z2_=+0.009 a.u.)**

O -0.48459100 1.83125100 0.78477800

C 1.84818000 2.42838400 3.18344100

Fe -0.33638100 0.36032100 0.12554300

H 4.59569900 0.46971800 -1.23987900

H -4.99638300 -1.56228300 -0.21482700

H 3.23190700 1.15293100 -3.47949800

H -4.15985800 -2.36292200 2.21248100

H 3.12166800 -1.47154100 3.40222400

H -4.00461300 -0.26408500 -3.30903600

H -1.91196000 0.56717000 -4.76769000

H 0.71534800 -2.17715800 4.45449300

N 1.28394400 0.39743200 -0.87045200

N -1.95786400 -0.50905300 0.61665100

N 0.52416800 -0.62177100 1.51845700

N -1.27024200 0.42570900 -1.52505300

C 3.52688000 0.54661700 -1.38012500

C -4.05590200 -1.32532800 0.26345500

C 2.84692000 0.88958900 -2.50364200

C -3.62718900 -1.73554000 1.51101000

C 2.53796100 0.21612000 -0.34641000

C -2.98574200 -0.55888700 -0.32225400

C 1.41186100 0.77730000 -2.20532900

C -2.27319400 -1.24007800 1.69964400

N 2.83039700 -0.24076700 0.87471400

N 0.45030800 0.91085300 -3.10236200

N -1.40546800 -1.52911700 2.70832000

C 1.87660100 -0.65817100 1.71683300

C -2.58626300 -0.03047000 -1.56061100

C -0.83160900 0.65342000 -2.77843300

C -0.11692400 -1.24217900 2.58520500

C 2.13014700 -1.29651000 3.00782000

C -3.02739500 0.01940700 -2.94371500

C -1.96173300 0.44270000 -3.69413800

C 0.92214400 -1.64593600 3.53493200

H 2.81126700 2.23345800 2.70476900

H 1.05052600 2.30521900 2.44553500

H 1.70192700 1.71523200 4.00124100

H 1.83467600 3.45117600 3.57355800

**TS1 (*F*_z2_=+0.009 a.u.)**

O -0.01989200 -0.01647000 0.22614700

C 0.23952300 0.18832500 2.79763400

Fe -0.10828800 -1.58938500 -0.47161000

H 4.65851500 -1.86053100 -2.28641400

H -4.94801900 -3.00901800 -0.21571700

H 3.13002600 -1.19059600 -4.41695000

H -3.93914400 -3.78158600 2.15481700

H 3.49686000 -3.48519600 2.57579300

H -4.15723600 -1.95510100 -3.48777600

H -2.15808100 -1.38977600 -5.18501700

H 1.15949600 -3.97968000 3.87593200

N 1.39178800 -1.72565600 -1.62268200

N -1.73102300 -2.31264900 0.20236300

N 0.80124600 -2.55567800 0.88884600

N -1.20074000 -1.49231100 -2.01963200

C 3.58609600 -1.73268700 -2.32674000

C -3.93650900 -2.86643800 0.13913700

C 2.82010500 -1.39060700 -3.40012200

C -3.41671400 -3.26494200 1.36105400

C 2.69357900 -1.96089800 -1.19548800

C -2.86612700 -2.27200500 -0.60716700

C 1.42328400 -1.39839300 -2.97278700

C -2.00825300 -2.93688600 1.37659700

N 3.05865600 -2.36708300 0.01305800

N 0.38245400 -1.23116900 -3.78533900

N -1.07329100 -3.25670400 2.30109400

C 2.16549200 -2.67475200 0.97252700

C -2.55434000 -1.81443700 -1.90732900

C -0.86747400 -1.35717000 -3.33157700

C 0.22120400 -3.08517400 2.05240600

C 2.48776800 -3.26028500 2.25808900

C -3.12507700 -1.76033900 -3.23196900

C -2.10221600 -1.47908600 -4.10810800

C 1.30748300 -3.50490000 2.91532200

H 1.16462000 -0.36226700 2.95553100

H 0.08893800 0.06063200 1.31654500

H -0.66349100 -0.30790700 3.14724100

H 0.28886600 1.26353700 2.95829000

**RC (*F*_z2_=+0.010 a.u.)**

O -0.47586900 1.82813500 0.79353200

C 1.83124800 2.41532500 3.20809400

Fe -0.33098600 0.36068500 0.12463600

H 4.59860000 0.46442800 -1.25310800

H -4.99004300 -1.56305000 -0.21185100

H 3.23013200 1.14830000 -3.48943800

H -4.15201700 -2.36319300 2.21551700

H 3.13248400 -1.47425100 3.39266700

H -4.00354300 -0.26663400 -3.30587900

H -1.91328300 0.56391700 -4.76798200

H 0.72730700 -2.17893300 4.45051600

N 1.28710100 0.39659300 -0.87449200

N -1.95207000 -0.50867000 0.61800000

N 0.53142900 -0.62350500 1.51565700

N -1.26760900 0.42849400 -1.52398400

C 3.52949700 0.54356600 -1.38981600

C -4.05000300 -1.32467600 0.26664800

C 2.84761000 0.88679600 -2.51195600

C -3.62021800 -1.73424800 1.51465800

C 2.54261500 0.21446800 -0.35348100

C -2.98083900 -0.55841100 -0.31955600

C 1.41306000 0.77607700 -2.20984600

C -2.26653900 -1.23910300 1.70198300

N 2.83694000 -0.24165800 0.86738400

N 0.44998800 0.91006400 -3.10497800

N -1.39740500 -1.52770400 2.71009700

C 1.88422700 -0.65949800 1.71119100

C -2.58297700 -0.02957000 -1.55885300

C -0.83063100 0.65383300 -2.77900900

C -0.10825200 -1.24222700 2.58396500

C 2.14000900 -1.29727600 3.00127600

C -3.02630400 0.01837700 -2.94171100

C -1.96221800 0.44111400 -3.69397900

C 0.93213800 -1.64608200 3.53121800

H 2.79827500 2.22418800 2.73585700

H 1.03991400 2.29106000 2.46334100

H 1.68065400 1.69937500 4.02285300

H 1.81160900 3.43773700 3.59953700

**TS1 (*F*_z2_=+0.010 a.u.)**

O -0.02024000 -0.01501700 0.23930400

C 0.25332800 0.18202200 2.82016300

Fe -0.11064700 -1.58340400 -0.47310200

H 4.65658400 -1.86212500 -2.28972800

H -4.94797500 -3.01197100 -0.22374700

H 3.12782000 -1.19574900 -4.42092100

H -3.94199500 -3.78090700 2.14985200

H 3.49417100 -3.47980600 2.57386300

H -4.15624300 -1.96602200 -3.49378800

H -2.15663800 -1.40306500 -5.19110000

H 1.15613400 -3.97489600 3.87510000

N 1.38921100 -1.72161400 -1.62382400

N -1.73375400 -2.30811700 0.20049900

N 0.79836000 -2.55051800 0.88851400

N -1.20255100 -1.48690900 -2.02199800

C 3.58438200 -1.73233800 -2.32925600

C -3.93788200 -2.86614200 0.13387300

C 2.81883600 -1.39232200 -3.40295400

C -3.41940800 -3.26218700 1.35741900

C 2.69135500 -1.95733700 -1.19671600

C -2.86715200 -2.27025200 -0.61055800

C 1.42138700 -1.39828700 -2.97425300

C -2.01163300 -2.93158100 1.37457400

N 3.05608000 -2.36140600 0.01218400

N 0.38106600 -1.23360800 -3.78782900

N -1.07673600 -3.25046800 2.30056000

C 2.16180300 -2.66870700 0.97196400

C -2.55469000 -1.81387100 -1.91143600

C -0.86824700 -1.35832000 -3.33452400

C 0.21802300 -3.07939400 2.05152300

C 2.48479800 -3.25391200 2.25749300

C -3.12500900 -1.76700500 -3.23744000

C -2.10257400 -1.48713500 -4.11345600

C 1.30421400 -3.49885900 2.91492600

H 1.17777300 -0.37200600 2.96798200

H 0.09380300 0.05384100 1.31962600

H -0.65109500 -0.31347400 3.16658800

H 0.30542200 1.25760100 2.97662000

**RC (*F*_z2_=-0.001 a.u.)**

O -0.03133600 1.98686000 0.40121000

C 0.37393700 2.07687300 3.87483200

Fe -0.06296900 0.43690300 -0.05079900

H 4.71915300 0.11316200 -1.80826200

H -4.89137000 -1.04861100 0.22047500

H 3.21656500 0.73133400 -3.97988400

H -3.87319100 -1.76730800 2.60100500

H 3.53378400 -1.42309100 3.06838300

H -4.09892300 -0.04598500 -3.05979700

H -2.09438000 0.48954600 -4.76830000

H 1.19152600 -1.86733800 4.36027300

N 1.45467100 0.26346700 -1.18738700

N -1.69481000 -0.23825300 0.65736200

N 0.84517900 -0.49806800 1.33775700

N -1.14620100 0.44736000 -1.61481200

C 3.64734800 0.23451400 -1.87789600

C -3.88365600 -0.88392100 0.57597800

C 2.89378600 0.54543000 -2.96507200

C -3.36617100 -1.25380100 1.79606000

C 2.73076100 0.03741600 -0.74897400

C -2.81310800 -0.26087000 -0.17288300

C 1.48477300 0.54838500 -2.54967500

C -1.95531600 -0.87235300 1.81369100

N 3.10072300 -0.35428000 0.47472400

N 0.45647900 0.69987200 -3.36863900

N -1.02282100 -1.18255800 2.74625000

C 2.20480100 -0.63342500 1.42666500

C -2.49712700 0.13594100 -1.48241500

C -0.81475200 0.57080700 -2.91084900

C 0.26396000 -1.00537300 2.49676500

C 2.52726600 -1.20921000 2.73771900

C -3.06499900 0.15080600 -2.81193800

C -2.03984700 0.42462400 -3.69055600

C 1.35428900 -1.43157800 3.38457800

H 1.32941300 1.59822900 4.10647100

H 0.25497400 2.15729600 2.79170400

H -0.43947400 1.47609300 4.29042300

H 0.35323200 3.07346500 4.32326200

**TS1 (*F*_z2_=-0.001 a.u.)**

O -0.00669200 -0.01677500 0.10664900

C 0.02938200 0.22404900 2.63351700

Fe -0.07150300 -1.64554600 -0.46277400

H 4.68313600 -1.87399800 -2.28264200

H -4.93640000 -2.94061300 -0.11252000

H 3.15178600 -1.16696600 -4.40511100

H -3.89704000 -3.74185000 2.23129500

H 3.54546500 -3.53911000 2.57720100

H -4.16423800 -1.83270300 -3.41343900

H -2.17701000 -1.25013400 -5.12633200

H 1.21795900 -4.01532100 3.87974000

N 1.42591300 -1.76886500 -1.62684100

N -1.68783300 -2.35100700 0.22076200

N 0.84400600 -2.60817100 0.88257400

N -1.17296500 -1.55019600 -2.00314000

C 3.60916400 -1.75918900 -2.32751500

C -3.90900600 -2.83906900 0.20885100

C 2.83622000 -1.39628800 -3.39692500

C -3.37578300 -3.25704700 1.41752600

C 2.72447900 -2.00776700 -1.20519900

C -2.83957400 -2.27804900 -0.56447600

C 1.44726200 -1.41161900 -2.97500100

C -1.95807300 -2.96993300 1.40554200

N 3.09618600 -2.43356600 0.00086300

N 0.39826700 -1.21801900 -3.77539900

N -1.01998200 -3.30111900 2.31049900

C 2.21819700 -2.73815600 0.96239300

C -2.53573500 -1.81784300 -1.86759600

C -0.85491100 -1.34704000 -3.31261900

C 0.27322100 -3.13501400 2.05508900

C 2.53847200 -3.32142700 2.24964800

C -3.12023900 -1.68790100 -3.17203500

C -2.09761800 -1.39501600 -4.05777300

C 1.36204600 -3.55772500 2.91109700

H 0.97545100 -0.24939000 2.89150400

H 0.00109200 0.11004500 1.29656600

H -0.85331500 -0.32519900 2.95624500

H -0.00970400 1.29827000 2.80577500

**RC (*F*_z2_=-0.002 a.u.)**

O -0.01619900 1.99127300 0.38455300

C 0.31827600 2.06361100 3.88746000

Fe -0.05432400 0.43837100 -0.05582600

H 4.72237900 0.10273400 -1.82208200

H -4.88817600 -1.02657200 0.23725400

H 3.21658600 0.71947000 -3.99239300

H -3.86389600 -1.74381200 2.61542400

H 3.54542800 -1.41940000 3.06218000

H -4.10224500 -0.03271000 -3.04956800

H -2.10113300 0.49261700 -4.76642600

H 1.20548200 -1.85444500 4.36007300

N 1.45967400 0.25756400 -1.19659100

N -1.68614500 -0.22832400 0.65919500

N 0.85489100 -0.49477000 1.33266600

N -1.14238600 0.44678300 -1.61736700

C 3.65049600 0.22425100 -1.89072900

C -3.87805700 -0.86638300 0.58789200

C 2.89493900 0.53449800 -2.97701600

C -3.35796100 -1.23558000 1.80643300

C 2.73589200 0.03041800 -0.75973700

C -2.80714000 -0.24942500 -0.16681900

C 1.48676700 0.54038400 -2.55920800

C -1.94465000 -0.85934700 1.81800800

N 3.10817900 -0.35912800 0.46394400

N 0.45678200 0.69344800 -3.37605900

N -1.01099400 -1.17125000 2.74731500

C 2.21451000 -0.63326600 1.41938800

C -2.49426100 0.14273100 -1.47911100

C -0.81460400 0.56877000 -2.91433100

C 0.27567700 -0.99749500 2.49490700

C 2.53877800 -1.20552600 2.73196600

C -3.06633400 0.15893800 -2.80575900

C -2.04227900 0.42766600 -3.68885500

C 1.36707400 -1.42333400 3.38210100

H 1.26943200 1.58243800 4.13131300

H 0.21640200 2.15156800 2.80314300

H -0.50205300 1.46193100 4.28758700

H 0.29253100 3.05700700 4.34227100

**TS1 (*F*_z2_=-0.002 a.u.)**

O -0.00490100 -0.01617900 0.09741400

C 0.01266800 0.22334700 2.62401700

Fe -0.06865700 -1.64979900 -0.46245900

H 4.68511200 -1.87457200 -2.28245100

H -4.93525500 -2.93399600 -0.10414700

H 3.15369700 -1.16526800 -4.40505100

H -3.89360600 -3.73693600 2.23793900

H 3.54944600 -3.54048100 2.57799300

H -4.16474700 -1.82335000 -3.40760700

H -2.17863100 -1.23944400 -5.12264000

H 1.22269800 -4.01526600 3.88063500

N 1.42845600 -1.77227900 -1.62823800

N -1.68438400 -2.35380200 0.22150500

N 0.84708200 -2.61227100 0.88124200

N -1.17085700 -1.55493800 -2.00294600

C 3.61098300 -1.76125100 -2.32816300

C -3.90658700 -2.83616800 0.21426500

C 2.83751900 -1.39714500 -3.39759500

C -3.37248400 -3.25536000 1.42216300

C 2.72673000 -2.01127100 -1.20677300

C -2.83714200 -2.27841200 -0.56137700

C 1.44912100 -1.41311600 -2.97618100

C -1.95406400 -2.97189400 1.40760300

N 3.09895900 -2.43819600 -0.00067100

N 0.39944000 -1.21755900 -3.77595700

N -1.01575600 -3.30377100 2.31079700

C 2.22236100 -2.74234600 0.96117200

C -2.53380600 -1.81848700 -1.86539600

C -0.85394000 -1.34641200 -3.31233700

C 0.27740300 -3.13813900 2.05471000

C 2.54261300 -3.32427200 2.24904700

C -3.11972400 -1.68275700 -3.16789600

C -2.09723000 -1.38884800 -4.05479300

C 1.36642000 -3.56003000 2.91080200

H 0.95904700 -0.24484600 2.89045600

H -0.00586300 0.11221200 1.29714900

H -0.86934900 -0.32924500 2.94294600

H -0.03268900 1.29728000 2.79682400

**RC (*F*_z2_=-0.003 a.u.)**

O -0.00184700 1.99538500 0.36733800

C 0.26532900 2.05078300 3.90118700

Fe -0.04591000 0.43953800 -0.06109400

H 4.72556100 0.09230200 -1.83547800

H -4.88503000 -1.00469100 0.25351400

H 3.21678300 0.70737800 -4.00473200

H -3.85499700 -1.72000100 2.62964500

H 3.55664800 -1.41531000 3.05641200

H -4.10538400 -0.01985100 -3.03993200

H -2.10763700 0.49541800 -4.76504300

H 1.21891000 -1.84099900 4.36016600

N 1.46454800 0.25143300 -1.20581200

N -1.67766600 -0.21879800 0.66062500

N 0.86429900 -0.49135800 1.32766700

N -1.13863500 0.44571200 -1.62030100

C 3.65359800 0.21388800 -1.90327900

C -3.87258500 -0.84902300 0.59937600

C 2.89615800 0.52335000 -2.98880400

C -3.35006100 -1.21727200 1.81656100

C 2.74086400 0.02331600 -0.77031900

C -2.80121800 -0.23843400 -0.16116500

C 1.48877900 0.53209200 -2.56877100

C -1.93429400 -0.84640700 1.82213500

N 3.11536800 -0.36389300 0.45342200

N 0.45720000 0.68665500 -3.38366200

N -0.99960200 -1.15976100 2.74832200

C 2.22390700 -0.63293500 1.41235600

C -2.49135000 0.14902800 -1.47634200

C -0.81438500 0.56631300 -2.91819200

C 0.28702100 -0.98935100 2.49315800

C 2.54988800 -1.20147400 2.72653700

C -3.06753200 0.16660600 -2.80010300

C -2.04453200 0.43033200 -3.68763900

C 1.37939000 -1.41463800 3.37988700

H 1.21163800 1.56667800 4.15776600

H 0.18035400 2.14456300 2.81585700

H -0.56205600 1.44955600 4.28694500

H 0.23534100 3.04174600 4.36063200

**TS1 (*F*_z2_=-0.003 a.u.)**

O -0.00290000 -0.01586400 0.08867400

C -0.00219300 0.22238000 2.61581900

Fe -0.06609000 -1.65382200 -0.46223900

H 4.68690000 -1.87521800 -2.28216500

H -4.93428200 -2.92772500 -0.09646000

H 3.15554900 -1.16392900 -4.40512700

H -3.89050900 -3.73226400 2.24403400

H 3.55296600 -3.54099200 2.57916800

H -4.16536100 -1.81445800 -3.40222500

H -2.18026100 -1.22943900 -5.11943400

H 1.22690300 -4.01439500 3.88181100

N 1.43072300 -1.77564500 -1.62977300

N -1.68133800 -2.35616400 0.22209800

N 0.84974900 -2.61613800 0.87986000

N -1.16896400 -1.55952300 -2.00307000

C 3.61261700 -1.76340700 -2.32882500

C -3.90443000 -2.83341200 0.21915200

C 2.83871900 -1.39825400 -3.39837600

C -3.36954600 -3.25373300 1.42634800

C 2.72867800 -2.01469400 -1.20834700

C -2.83498400 -2.27860100 -0.55859000

C 1.45080000 -1.41479700 -2.97750100

C -1.95048400 -2.97358700 1.40939400

N 3.10137000 -2.44249100 -0.00212500

N 0.40050000 -1.21739900 -3.77681100

N -1.01199800 -3.30606100 2.31095300

C 2.22608300 -2.74611100 0.96006500

C -2.53204200 -1.81916000 -1.86357400

C -0.85309300 -1.34598900 -3.31234400

C 0.28111700 -3.14085400 2.05431400

C 2.54629800 -3.32641800 2.24870100

C -3.11934100 -1.67797000 -3.16421000

C -2.09695500 -1.38318000 -4.05226100

C 1.37029800 -3.56165600 2.91071800

H 0.94474400 -0.24034800 2.88986800

H -0.01194300 0.11395700 1.29805800

H -0.88318500 -0.33388800 2.93125800

H -0.05403000 1.29598300 2.78916300

**RC (*F*_z2_=-0.004 a.u.)**

O 0.01117300 1.99929900 0.34952900

C 0.21832000 2.03868600 3.91542900

Fe -0.03844200 0.44052700 -0.06668600

H 4.72833500 0.08201900 -1.84767100

H -4.88254900 -0.98330000 0.26850900

H 3.21711400 0.69469300 -4.01649100

H -3.84745400 -1.69570400 2.64322000

H 3.56632800 -1.40989100 3.05190300

H -4.10844000 -0.00833200 -3.03167600

H -2.11373900 0.49692000 -4.76464700

H 1.23050600 -1.82600800 4.36104600

N 1.46878300 0.24504200 -1.21488100

N -1.67017700 -0.20945600 0.66140500

N 0.87249900 -0.48748400 1.32294300

N -1.13535700 0.44380800 -1.62390600

C 3.65628700 0.20347700 -1.91497300

C -3.86794700 -0.83195100 0.60986700

C 2.89724200 0.51177500 -3.00004900

C -3.34335200 -1.19874400 1.82602300

C 2.74512700 0.01629600 -0.78027100

C -2.79597900 -0.22802300 -0.15639100

C 1.49053300 0.52325200 -2.57821000

C -1.92518600 -0.83317400 1.82587900

N 3.12153100 -0.36815200 0.44372800

N 0.45759200 0.67899500 -3.39152500

N -0.98973300 -1.14753000 2.74927500

C 2.23209600 -0.63194200 1.40601600

C -2.48882000 0.15438100 -1.47460800

C -0.81428900 0.56290600 -2.92269600

C 0.29695000 -0.98038200 2.49167800

C 2.55952100 -1.19626600 2.72204100

C -3.06876500 0.17306200 -2.79558300

C -2.04664100 0.43181300 -3.68737900

C 1.39006800 -1.40467900 3.37838100

H 1.16101300 1.55274000 4.18163700

H 0.14608100 2.13699900 2.82948900

H -0.61397400 1.43768800 4.29057600

H 0.18490500 3.02770900 4.37843100

**TS1 (*F*_z2_=-0.004 a.u.)**

O -0.00090000 -0.01624800 0.08033800

C -0.01563300 0.22244300 2.60850900

Fe -0.06358800 -1.65805800 -0.46216800

H 4.68863600 -1.87750300 -2.28203500

H -4.93368700 -2.92021200 -0.08878400

H 3.15756900 -1.16390700 -4.40544500

H -3.88807500 -3.72611300 2.25033200

H 3.55596000 -3.54240800 2.58034300

H -4.16607600 -1.80456600 -3.39706700

H -2.18186600 -1.21879000 -5.11646700

H 1.23046000 -4.01361700 3.88312000

N 1.43291700 -1.77962500 -1.63150800

N -1.67848800 -2.35852800 0.22241800

N 0.85212600 -2.62016700 0.87850500

N -1.16701700 -1.56453400 -2.00356400

C 3.61423300 -1.76689000 -2.32967500

C -3.90262700 -2.82973600 0.22398100

C 2.84002700 -1.40056200 -3.39937700

C -3.36712400 -3.25101800 1.43060400

C 2.73048200 -2.01907900 -1.21005700

C -2.83297200 -2.27849400 -0.55604000

C 1.45252400 -1.41725500 -2.97900400

C -1.94731100 -2.97472900 1.41112300

N 3.10351000 -2.44769000 -0.00368200

N 0.40168100 -1.21768400 -3.77792000

N -1.00872100 -3.30785700 2.31115100

C 2.22943700 -2.75044500 0.95895100

C -2.53026500 -1.81981800 -1.86208200

C -0.85215800 -1.34575800 -3.31260200

C 0.28439800 -3.14349300 2.05394800

C 2.54951200 -3.32916100 2.24838300

C -3.11900100 -1.67265100 -3.16080200

C -2.09668000 -1.37716100 -4.05000600

C 1.37364400 -3.56346700 2.91072800

H 0.93438700 -0.22980600 2.88946100

H -0.01761600 0.11549300 1.29912200

H -0.89242400 -0.34170100 2.92176000

H -0.07919300 1.29552600 2.78150700

**RC (*F*_z2_=-0.005 a.u.)**

O 0.03181700 2.00264800 0.33108000

C 0.15607600 2.02326200 3.93412500

Fe -0.02781700 0.44117700 -0.07275900

H 4.73230800 0.06945700 -1.86442400

H -4.87733100 -0.95918500 0.28730000

H 3.21704900 0.68073300 -4.03141400

H -3.83551700 -1.66960500 2.65957900

H 3.58085100 -1.40464000 3.04472800

H -4.11135900 0.00557200 -3.02006800

H -2.12101900 0.49919100 -4.76280300

H 1.24789800 -1.81007300 4.36117100

N 1.47472900 0.23648100 -1.22597800

N -1.65940200 -0.19887200 0.66334800

N 0.88431400 -0.48538900 1.31659800

N -1.13058800 0.44287600 -1.62682300

C 3.66013600 0.19093900 -1.93032700

C -3.86026000 -0.81269300 0.62332700

C 2.89868200 0.49857000 -3.01426700

C -3.33274100 -1.17850300 1.83796000

C 2.75144300 0.00708500 -0.79318400

C -2.78814500 -0.21579800 -0.14939200

C 1.49298300 0.51294400 -2.58946600

C -1.91203800 -0.81901500 1.83091700

N 3.13065000 -0.37429300 0.43086000

N 0.45803100 0.67080400 -3.40025400

N -0.97529200 -1.13505300 2.75057300

C 2.24400300 -0.63237800 1.39731600

C -2.48473500 0.16141000 -1.47102800

C -0.81374000 0.55990100 -2.92689500

C 0.31138000 -0.97190800 2.48943300

C 2.57378900 -1.19142300 2.71536400

C -3.06959200 0.18134400 -2.78864900

C -2.04899200 0.43423400 -3.68571300

C 1.40580600 -1.39450700 3.37570100

H 1.09321200 1.53537000 4.21596900

H 0.10358600 2.12594900 2.84733300

H -0.68330900 1.42268000 4.29347400

H 0.11653000 3.01050400 4.40011700

**TS1 (*F*_z2_=-0.005 a.u.)**

O 0.00023900 -0.01665800 0.07386300

C -0.02834900 0.22060900 2.60391300

Fe -0.06146000 -1.66157500 -0.46233500

H 4.69030300 -1.87718500 -2.28160400

H -4.93292000 -2.91420500 -0.08233400

H 3.15948700 -1.16217600 -4.40578000

H -3.88554900 -3.72097900 2.25563800

H 3.55892400 -3.54170500 2.58160500

H -4.16647400 -1.79801000 -3.39311600

H -2.18309100 -1.21126900 -5.11466200

H 1.23399800 -4.01194600 3.88419300

N 1.43484700 -1.78222500 -1.63337900

N -1.67575500 -2.36116500 0.22203200

N 0.85430400 -2.62337600 0.87684200

N -1.16546200 -1.56805100 -2.00441600

C 3.61574300 -1.76821700 -2.33039700

C -3.90076600 -2.82715100 0.22777000

C 2.84123100 -1.40119200 -3.40038800

C -3.36469400 -3.24912700 1.43393200

C 2.73209500 -2.02159400 -1.21170400

C -2.83097500 -2.27905700 -0.55434300

C 1.45403800 -1.41857700 -2.98064900

C -1.94423500 -2.97612200 1.41211900

N 3.10548500 -2.45084500 -0.00518700

N 0.40271100 -1.21762000 -3.77945000

N -1.00553900 -3.30959000 2.31077900

C 2.23255900 -2.75324400 0.95777400

C -2.52865300 -1.82059600 -1.86125500

C -0.85137600 -1.34551800 -3.31333300

C 0.28752700 -3.14561700 2.05318000

C 2.55263300 -3.33025800 2.24803000

C -3.11856100 -1.66937200 -3.15841800

C -2.09631400 -1.37308900 -4.04870800

C 1.37690600 -3.56426000 2.91053800

H 0.92022000 -0.23043400 2.89179000

H -0.02315800 0.11571500 1.30205900

H -0.90644800 -0.34320000 2.91421400

H -0.09339000 1.29361400 2.77718600

**RC (*F*_z2_=-0.006 a.u.)**

O 0.03490100 2.00594600 0.31346100

C 0.12943300 2.01100600 3.95218800

Fe -0.02386800 0.44159200 -0.07840500

H 4.73406800 0.06423000 -1.87174100

H -4.87669000 -0.94325100 0.29784800

H 3.21784500 0.67072100 -4.04003200

H -3.83158000 -1.64830800 2.67027600

H 3.58636700 -1.39655000 3.04323800

H -4.11406400 0.01214200 -3.01617300

H -2.12572000 0.49839300 -4.76486000

H 1.25462600 -1.79474100 4.36296100

N 1.47716100 0.23241100 -1.23331800

N -1.65494200 -0.19279200 0.66205800

N 0.88915200 -0.47925200 1.31345000

N -1.12890700 0.43812200 -1.63189500

C 3.66175500 0.18452900 -1.93819500

C -3.85796200 -0.80016700 0.63023200

C 2.89942400 0.48977700 -3.02256800

C -3.32927600 -1.16322200 1.84471100

C 2.75372100 0.00347600 -0.80014100

C -2.78506600 -0.20979200 -0.14752700

C 1.49397800 0.50562700 -2.59735100

C -1.90652500 -0.80806500 1.83303000

N 3.13402100 -0.37483300 0.42443900

N 0.45833100 0.66305000 -3.40778400

N -0.96954200 -1.12355900 2.75092200

C 2.24878000 -0.62855300 1.39350400

C -2.48344800 0.16256500 -1.47219500

C -0.81406900 0.55469400 -2.93253500

C 0.31731500 -0.96206300 2.48873600

C 2.57938000 -1.18391900 2.71323500

C -3.07090600 0.18365700 -2.78748500

C -2.05062200 0.43292100 -3.68785100

C 1.41203400 -1.38342000 3.37552800

H 1.06966700 1.52708100 4.23047300

H 0.07376800 2.11762700 2.86579300

H -0.70595000 1.40565400 4.31265300

H 0.08676800 2.99613000 4.42201700

**TS1 (*F*_z2_=-0.006 a.u.)**

O 0.00097500 -0.01775000 0.06771000

C -0.03736100 0.21922700 2.60008500

Fe -0.05984500 -1.66516300 -0.46272200

H 4.69153400 -1.87724700 -2.28123100

H -4.93268900 -2.90830000 -0.07610800

H 3.16105800 -1.16127000 -4.40640800

H -3.88365400 -3.71540800 2.26095100

H 3.56126400 -3.54000200 2.58335700

H -4.16738400 -1.79192700 -3.38952000

H -2.18479300 -1.20449700 -5.11330000

H 1.23688600 -4.00896400 3.88585100

N 1.43626500 -1.78522300 -1.63551400

N -1.67360900 -2.36376200 0.22146700

N 0.85590500 -2.62631900 0.87521600

N -1.16436500 -1.57198900 -2.00566600

C 3.61681700 -1.76998600 -2.33126700

C -3.89947300 -2.82459600 0.23136300

C 2.84205600 -1.40251700 -3.40165700

C -3.36289900 -3.24693600 1.43718800

C 2.73317800 -2.02434100 -1.21344600

C -2.82951300 -2.27974300 -0.55291000

C 1.45509000 -1.42055300 -2.98257200

C -1.94177400 -2.97714400 1.41303100

N 3.10688900 -2.45382600 -0.00665300

N 0.40332200 -1.21827800 -3.78137000

N -1.00299500 -3.31065900 2.31052800

C 2.23505400 -2.75563600 0.95674600

C -2.52752600 -1.82180700 -1.86078100

C -0.85104200 -1.34590300 -3.31441000

C 0.29001900 -3.14710200 2.05260200

C 2.55513300 -3.33049900 2.24805800

C -3.11862900 -1.66661900 -3.15641700

C -2.09645000 -1.36970200 -4.04782800

C 1.37952800 -3.56404100 2.91078500

H 0.91141300 -0.22803500 2.89332700

H -0.02708600 0.11573400 1.30510500

H -0.91476700 -0.34651400 2.90898000

H -0.10644700 1.29202900 2.77342800

**RC (*F*_z2_=-0.007 a.u.)**

O 0.04701300 2.00932900 0.29345000

C 0.08654400 1.99546000 3.97654700

Fe -0.01655300 0.44216100 -0.08543300

H 4.73685900 0.05424800 -1.88461500

H -4.87362000 -0.92221600 0.31205700

H 3.21819800 0.65657100 -4.05304100

H -3.82369900 -1.62223900 2.68389400

H 3.59629800 -1.38786300 3.03892000

H -4.11684900 0.02159500 -3.00927000

H -2.13176800 0.49727400 -4.76625600

H 1.26659300 -1.77600800 4.36422300

N 1.48122700 0.22531300 -1.24362400

N -1.64732400 -0.18374400 0.66155000

N 0.89740600 -0.47414600 1.30815800

N -1.12568900 0.43463800 -1.63710200

C 3.66441200 0.17393900 -1.95073400

C -3.85289800 -0.78319700 0.63994600

C 2.90047400 0.47712900 -3.03495600

C -3.32227100 -1.14367100 1.85389600

C 2.75792100 -0.00339700 -0.81093700

C -2.77950400 -0.20029900 -0.14381300

C 1.49565200 0.49538300 -2.60811500

C -1.89725400 -0.79398600 1.83626600

N 3.14014400 -0.37782900 0.41415400

N 0.45867800 0.65338300 -3.41725100

N -0.95957500 -1.10950700 2.75157300

C 2.25703500 -0.62586000 1.38682100

C -2.48063900 0.16628200 -1.47208300

C -0.81396700 0.54929400 -2.93878900

C 0.32744300 -0.95100400 2.48707400

C 2.58922900 -1.17581500 2.70873100

C -3.07194600 0.18805200 -2.78445400

C -2.05262800 0.43204200 -3.68933600

C 1.42290800 -1.37030900 3.37416500

H 1.02684600 1.51355600 4.25810200

H 0.03604700 2.10656500 2.89017200

H -0.74857100 1.38668700 4.33158300

H 0.03924800 2.97838100 4.45018800

**TS1 (*F*_z2_=-0.007 a.u.)**

O 0.00189700 -0.01926000 0.06239400

C -0.04685000 0.21766000 2.59743600

Fe -0.05813100 -1.66855100 -0.46318500

H 4.69289500 -1.87773500 -2.28092600

H -4.93231400 -2.90261700 -0.07056800

H 3.16289400 -1.16076500 -4.40721800

H -3.88175000 -3.71021600 2.26559900

H 3.56348700 -3.53813300 2.58521100

H -4.16810800 -1.78587200 -3.38629400

H -2.18622000 -1.19792500 -5.11230900

H 1.23959700 -4.00579800 3.88755200

N 1.43780800 -1.78792200 -1.63770700

N -1.67148900 -2.36584300 0.22082100

N 0.85750600 -2.62902500 0.87357500

N -1.16312700 -1.57556200 -2.00716100

C 3.61804100 -1.77201200 -2.33223500

C -3.89811500 -2.82209400 0.23443200

C 2.84311700 -1.40409000 -3.40306700

C -3.36112200 -3.24484400 1.43992600

C 2.73432700 -2.02705400 -1.21521200

C -2.82799300 -2.28008000 -0.55175800

C 1.45631900 -1.42249700 -2.98460000

C -1.93935500 -2.97794700 1.41363600

N 3.10829500 -2.45670100 -0.00812200

N 0.40417700 -1.21892700 -3.78349600

N -1.00054900 -3.31154600 2.31008200

C 2.23747400 -2.75782500 0.95575300

C -2.52626800 -1.82278600 -1.86058500

C -0.85051000 -1.34622200 -3.31567600

C 0.29241500 -3.14837200 2.05197800

C 2.55753000 -3.33054500 2.24815900

C -3.11853200 -1.66383700 -3.15475900

C -2.09638800 -1.36642100 -4.04728500

C 1.38201900 -3.56359900 2.91108900

H 0.90241000 -0.22520400 2.89594700

H -0.03142900 0.11536400 1.30877500

H -0.92317200 -0.35065100 2.90487000

H -0.12070200 1.29018600 2.77092700

**RC (*F*_z2_=-0.008 a.u.)**

O 0.05587000 2.01292200 0.27108800

C 0.05598100 1.97884800 4.00834200

Fe -0.01163300 0.44278400 -0.09380600

H 4.73861200 0.04815700 -1.89593900

H -4.87166200 -0.90525100 0.32320900

H 3.21809700 0.64376600 -4.06564400

H -3.81764600 -1.59735600 2.69562800

H 3.60391800 -1.37411100 3.03616400

H -4.11997200 0.02564500 -3.00562200

H -2.13774700 0.49166200 -4.77016500

H 1.27596800 -1.75296400 4.36627000

N 1.48364000 0.21855500 -1.25442300

N -1.64187100 -0.17540400 0.65926100

N 0.90338000 -0.46740600 1.30255700

N -1.12400500 0.42925200 -1.64419200

C 3.66593200 0.16599000 -1.96239600

C -3.84931700 -0.76902700 0.64712200

C 2.90068300 0.46581700 -3.04704200

C -3.31711200 -1.12544500 1.86116400

C 2.76051300 -0.00824400 -0.82132500

C -2.77559800 -0.19320500 -0.14240300

C 1.49620500 0.48507300 -2.61936600

C -1.89018500 -0.77998800 1.83812500

N 3.14433400 -0.37769200 0.40464500

N 0.45820000 0.64237600 -3.42790400

N -0.95205600 -1.09400500 2.75151600

C 2.26312400 -0.62026800 1.38063500

C -2.47914300 0.16693100 -1.47447000

C -0.81480600 0.54158200 -2.94686400

C 0.33520500 -0.93770800 2.48520000

C 2.59675500 -1.16361300 2.70518400

C -3.07363000 0.18814200 -2.78420500

C -2.05505400 0.42730600 -3.69324600

C 1.43129600 -1.35347300 3.37338900

H 0.99834200 1.50152400 4.29077400

H 0.00769900 2.09486700 2.92216000

H -0.77636800 1.36417900 4.35946700

H 0.00229200 2.95918200 4.48633100

**TS1 (*F*_z2_=-0.008 a.u.)**

O 0.00265500 -0.02124500 0.05788600

C -0.05489700 0.21584600 2.59605700

Fe -0.05669400 -1.67175500 -0.46390100

H 4.69407300 -1.87742600 -2.28065400

H -4.93209900 -2.89719000 -0.06533100

H 3.16452500 -1.16020500 -4.40832400

H -3.88003500 -3.70468100 2.27016900

H 3.56555100 -3.53478200 2.58734600

H -4.16893900 -1.78184100 -3.38368900

H -2.18773400 -1.19377200 -5.11211300

H 1.24216800 -4.00141500 3.88953500

N 1.43906100 -1.79046600 -1.64017700

N -1.66953800 -2.36804300 0.21983300

N 0.85889000 -2.63132900 0.87178900

N -1.16222900 -1.57865800 -2.00898000

C 3.61906100 -1.77350900 -2.33333900

C -3.89694400 -2.81974400 0.23720700

C 2.84397700 -1.40553400 -3.40473700

C -3.35955700 -3.24257000 1.44248100

C 2.73522300 -2.02924400 -1.21708600

C -2.82665300 -2.28069400 -0.55100200

C 1.45727100 -1.42454900 -2.98693800

C -1.93712300 -2.97849200 1.41401800

N 3.10946900 -2.45869800 -0.00962500

N 0.40477900 -1.22014500 -3.78609700

N -0.99829600 -3.31189400 2.30958800

C 2.23962500 -2.75919200 0.95477200

C -2.52529300 -1.82399900 -1.86075600

C -0.85024600 -1.34712300 -3.31735400

C 0.29459700 -3.14905100 2.05133800

C 2.55974100 -3.32939600 2.24844300

C -3.11862700 -1.66247700 -3.15367900

C -2.09653300 -1.36476600 -4.04738500

C 1.38432700 -3.56210400 2.91158300

H 0.89412700 -0.22432400 2.89951800

H -0.03507400 0.11439800 1.31339500

H -0.93103400 -0.35341500 2.90247800

H -0.13149400 1.28822000 2.76972900

**RC (*F*_z2_=-0.009 a.u.)**

O 0.06307900 2.01693600 0.24544000

C 0.02867600 1.95902000 4.05337300

Fe -0.00665700 0.44363400 -0.10452700

H 4.74068400 0.03994600 -1.90837000

H -4.86999700 -0.88616600 0.33223100

H 3.21883800 0.62709100 -4.08019300

H -3.81262300 -1.56818700 2.70615300

H 3.61065500 -1.35812400 3.03315900

H -4.12255300 0.02847300 -3.00493200

H -2.14269700 0.48268100 -4.77708600

H 1.28415300 -1.72609800 4.36791300

N 1.48631800 0.21150400 -1.26698200

N -1.63660000 -0.16607400 0.65460300

N 0.90907000 -0.45817800 1.29597700

N -1.12207000 0.42325200 -1.65394300

C 3.66781700 0.15620100 -1.97542300

C -3.84606100 -0.75281000 0.65222300

C 2.90152200 0.45179300 -3.06092100

C -3.31264800 -1.10403400 1.86687200

C 2.76325800 -0.01346900 -0.83303100

C -2.77169600 -0.18537000 -0.14344300

C 1.49730900 0.47296400 -2.63269000

C -1.88370800 -0.76342800 1.83832300

N 3.14830800 -0.37692800 0.39417600

N 0.45845900 0.62887200 -3.44102000

N -0.94531800 -1.07514700 2.75025000

C 2.26881600 -0.61284100 1.37365200

C -2.47736800 0.16722300 -1.47962400

C -0.81505900 0.53203300 -2.95764000

C 0.34228200 -0.92130000 2.48243600

C 2.60352400 -1.14891000 2.70116900

C -3.07479100 0.18688600 -2.78685200

C -2.05672600 0.42020000 -3.70007200

C 1.43874600 -1.33335700 3.37206200

H 0.97656000 1.49113800 4.33303500

H -0.02129600 2.08168600 2.96766800

H -0.79637600 1.33307300 4.40161000

H -0.03566300 2.93542700 4.53774000

**TS1 (*F*_z2_=-0.009 a.u.)**

O 0.00292000 -0.02396200 0.05436900

C -0.06021300 0.21361400 2.59592000

Fe -0.05557500 -1.67492500 -0.46475200

H 4.69501200 -1.87746500 -2.28040900

H -4.93233200 -2.89155700 -0.06071700

H 3.16609800 -1.15964000 -4.40948100

H -3.87896900 -3.69918900 2.27412300

H 3.56696800 -3.53175300 2.58947100

H -4.17003500 -1.77714700 -3.38147400

H -2.18939800 -1.18889200 -5.11218300

H 1.24401200 -3.99721600 3.89144500

N 1.44004200 -1.79289400 -1.64277100

N -1.66804500 -2.37014300 0.21856900

N 0.85981800 -2.63363500 0.86991100

N -1.16158300 -1.58138400 -2.01105900

C 3.61986100 -1.77520900 -2.33450600

C -3.89625900 -2.81726000 0.23945300

C 2.84471900 -1.40702400 -3.40647400

C -3.35859800 -3.24024300 1.44450200

C 2.73578700 -2.03155200 -1.21901600

C -2.82570200 -2.28107700 -0.55064600

C 1.45802900 -1.42647100 -2.98935400

C -1.93543500 -2.97897700 1.41398300

N 3.11020100 -2.46085700 -0.01118700

N 0.40525700 -1.22101700 -3.78883200

N -0.99666300 -3.31225800 2.30880700

C 2.24122700 -2.76068100 0.95373700

C -2.52463100 -1.82486700 -1.86124400

C -0.85017300 -1.34763400 -3.31918600

C 0.29617800 -3.14977800 2.05057000

C 2.56133300 -3.32845200 2.24869700

C -3.11899100 -1.66057100 -3.15293200

C -2.09688600 -1.36257100 -4.04775700

C 1.38597200 -3.56073900 2.91202700

H 0.88985800 -0.22197500 2.90297400

H -0.03766500 0.11282900 1.31891100

H -0.93459700 -0.35835700 2.90254400

H -0.14146100 1.28566000 2.76996700

**RC (*F*_z2_=-0.010 a.u.)**

O 0.06751200 2.02389900 0.21003600

C 0.01574300 1.93154400 4.12684700

Fe -0.00488100 0.44670900 -0.12065700

H 4.74148600 0.03421300 -1.92130200

H -4.86980600 -0.87039900 0.33455200

H 3.21954100 0.60592500 -4.09795300

H -3.81036800 -1.53469500 2.71277000

H 3.61363900 -1.32995500 3.03120700

H -4.12505300 0.02046000 -3.01207300

H -2.14690100 0.46175600 -4.79132700

H 1.28801400 -1.68568700 4.36983900

N 1.48689200 0.20438200 -1.28335900

N -1.63426200 -0.15447800 0.64458900

N 0.91129600 -0.44344500 1.28640200

N -1.12168200 0.41345300 -1.67001000

C 3.66837900 0.14763700 -1.99013900

C -3.84485100 -0.73747800 0.65138300

C 2.90163700 0.43546500 -3.07784400

C -3.31090400 -1.07964300 1.86805900

C 2.76380900 -0.01591400 -0.84692000

C -2.76992000 -0.17902400 -0.15067700

C 1.49730900 0.45773700 -2.65026500

C -1.88046600 -0.74209900 1.83447800

N 3.14956500 -0.37004000 0.38259900

N 0.45817200 0.60943300 -3.45971300

N -0.94238700 -1.04833300 2.74639700

C 2.27127900 -0.59821000 1.36524100

C -2.47694000 0.16270000 -1.49167600

C -0.81600300 0.51613200 -2.97476800

C 0.34573300 -0.89659200 2.47746900

C 2.60661000 -1.12360000 2.69699900

C -3.07632300 0.17702700 -2.79683700

C -2.05838200 0.40372800 -3.71400200

C 1.44220500 -1.30188600 3.37027000

H 0.97185800 1.47748300 4.40110700

H -0.03638900 2.06780900 3.04254400

H -0.79823200 1.28729500 4.46737500

H -0.06537300 2.90030800 4.62364900

**TS1 (*F*_z2_=-0.010 a.u.)**

O 0.00285800 -0.02702600 0.05163400

C -0.06521100 0.21077200 2.59699000

Fe -0.05456400 -1.67783500 -0.46579800

H 4.69594800 -1.87677300 -2.28006400

H -4.93249200 -2.88650900 -0.05679400

H 3.16773000 -1.15872700 -4.41073800

H -3.87790300 -3.69412400 2.27749300

H 3.56826100 -3.52775200 2.59180500

H -4.17100800 -1.77385700 -3.37983200

H -2.19088000 -1.18560700 -5.11289300

H 1.24571700 -3.99230800 3.89349200

N 1.44093900 -1.79486500 -1.64547600

N -1.66662000 -2.37201300 0.21710100

N 0.86068600 -2.63552800 0.86795500

N -1.16101600 -1.58343700 -2.01336300

C 3.62065100 -1.77632400 -2.33566400

C -3.89557100 -2.81512900 0.24114600

C 2.84549400 -1.40813100 -3.40829500

C -3.35768400 -3.23816400 1.44600900

C 2.73627800 -2.03327000 -1.22093100

C -2.82478200 -2.28146800 -0.55066700

C 1.45875700 -1.42813200 -2.99191400

C -1.93380500 -2.97933200 1.41360700

N 3.11083900 -2.46226700 -0.01271900

N 0.40575500 -1.22199600 -3.79187700

N -0.99514300 -3.31236400 2.30784000

C 2.24267100 -2.76151500 0.95273500

C -2.52403200 -1.82568200 -1.86205200

C -0.85010900 -1.34832000 -3.32130900

C 0.29762800 -3.15010300 2.04977400

C 2.56279300 -3.32670200 2.24908000

C -3.11930900 -1.65955400 -3.15267800

C -2.09717700 -1.36139700 -4.04864500

C 1.38746500 -3.55870100 2.91257900

H 0.88355700 -0.22533000 2.90764100

H -0.04025200 0.11085200 1.32542200

H -0.94088200 -0.35947100 2.90344500

H -0.14550200 1.28282100 2.77196200

**Coordinates of the doublet state under the *F*_z1_**

**RC (*F*_z1_=+0.002 a.u.)**

Fe 0.05559500 -0.31098100 0.35408000

O 0.18529100 -0.79620200 1.88958400

H 5.00035200 0.62906400 -0.52089000

H -4.85737700 0.26135000 -0.70490700

H 3.95386900 3.02134600 0.21270600

H -4.33206400 -2.25930200 -1.47415400

H 2.87263100 -3.98005800 -1.81741600

H -3.43720900 3.34124100 0.15402000

H -1.13040000 4.68544300 0.46293900

H 0.31548600 -4.86421200 -1.99739800

N 1.71577500 0.55630800 0.00869700

N -1.73164900 -0.73267100 -0.14302900

N 0.61834200 -1.82378500 -0.65665900

N -0.74178300 1.41373800 0.44921400

C 3.97648500 0.87394100 -0.27589700

C -3.91989800 -0.26866400 -0.60899800

C 3.45228400 2.07095000 0.09560700

C -3.65403600 -1.55860000 -1.00727900

C 2.87063900 -0.08750000 -0.34672100

C -2.68816600 0.27960400 -0.08496100

C 2.00344100 1.89478500 0.25910900

C -2.24173800 -1.82239100 -0.74268300

N 2.99010200 -1.35612000 -0.74854800

N 1.14804300 2.87535600 0.49589100

N -1.51662500 -2.90429200 -1.11672400

C 1.92315300 -2.14145500 -0.92307200

C -2.11892600 1.51446300 0.25712400

C -0.18627400 2.63681300 0.49601600

C -0.19529900 -2.87338800 -1.07419500

C 1.96289500 -3.48932800 -1.50150100

C -2.44852900 2.92178600 0.27882400

C -1.26852400 3.61373500 0.43249100

C 0.68224600 -3.93351200 -1.58774300

C -0.65016200 1.98334900 3.82295300

H 0.14654600 2.70573900 3.62500000

H -0.40868300 1.03474600 3.33789000

H -0.74843800 1.83740000 4.90249800

H -1.59104800 2.36986200 3.42170500

**TS1 (*F*_z1_=+0.002 a.u.)**

Fe -0.06734700 0.09202600 -0.05227300

O -0.10203000 -0.03439600 1.65926200

H 4.99443700 0.05621500 -0.66713200

H -4.83654700 0.98773300 -1.44227000

H 4.29411000 2.67728500 -0.74151200

H -4.70025800 -1.69603400 -1.37715800

H 2.22025600 -4.39347600 -0.73847900

H -2.94383600 4.01140800 -1.38412500

H -0.47568400 5.06731300 -1.23590000

H -0.43735800 -4.92377700 -0.88703900

N 1.71785400 0.59496700 -0.48057900

N -1.85882400 -0.19854000 -0.61702100

N 0.28195700 -1.69829100 -0.55495500

N -0.58383500 1.83695200 -0.56229400

C 4.00607500 0.48917600 -0.60399200

C -3.97567000 0.38752800 -1.18161300

C 3.65199200 1.81380400 -0.63852300

C -3.90846900 -0.99510600 -1.15244700

C 2.79256600 -0.28688000 -0.51771800

C -2.66966400 0.89042200 -0.85672900

C 2.20989000 1.89333300 -0.57306600

C -2.55181700 -1.36826600 -0.82334400

N 2.71322600 -1.61633900 -0.53002800

N 1.49600100 3.01872700 -0.68092500

N -1.99098100 -2.57669000 -0.80886300

C 1.55433400 -2.27216100 -0.56862300

C -1.91749000 2.09579000 -0.83043800

C 0.16413500 2.98419800 -0.72400600

C -0.66472400 -2.71558400 -0.68508500

C 1.39426200 -3.69827500 -0.68464900

C -2.03209200 3.49420700 -1.11844500

C -0.76046800 4.04222900 -1.04441000

C 0.04808600 -3.96694800 -0.75484800

C -0.31487200 -2.36117500 2.58886900

H 0.54616600 -2.88437500 2.17846600

H -0.20128500 -1.13520700 2.10370200

H -1.27665700 -2.70137800 2.21065900

H -0.27804200 -2.17509900 3.66108300

**RC (*F*_z1_=+0.004 a.u.)**

Fe 0.01775100 -0.32019000 0.37182500

O 0.12066900 -0.80441400 1.91180200

H 4.96943000 0.63672900 -0.45427800

H -4.86380800 0.26059200 -0.81771100

H 3.90790100 3.02651100 0.26659000

H -4.32149100 -2.25861400 -1.57907400

H 2.87660000 -3.96446100 -1.79278100

H -3.47079000 3.32776100 0.07183500

H -1.17282500 4.67607600 0.42766800

H 0.32511500 -4.85358100 -2.01746500

N 1.68146400 0.55119400 0.05437400

N -1.75914300 -0.74426400 -0.15935900

N 0.60038900 -1.82809400 -0.63434300

N -0.78313600 1.40415500 0.45404100

C 3.94425000 0.87677200 -0.20990500

C -3.93310600 -0.27436600 -0.68895700

C 3.41231600 2.07229000 0.15509100

C -3.65879000 -1.56318700 -1.08316000

C 2.84250900 -0.08820600 -0.28947200

C -2.71550500 0.26906800 -0.12736600

C 1.96295300 1.89098100 0.30536300

C -2.25496300 -1.83022100 -0.77869700

N 2.97189300 -1.35541600 -0.69076300

N 1.10306100 2.87012400 0.52953400

N -1.52292600 -2.91012100 -1.14091300

C 1.90963700 -2.14130100 -0.88580300

C -2.15524900 1.50351500 0.22787500

C -0.23050300 2.62881000 0.50466400

C -0.20310700 -2.87663000 -1.07476300

C 1.96181900 -3.48308800 -1.47703300

C -2.48650300 2.90999200 0.23223100

C -1.31069400 3.60402700 0.41029000

C 0.68415400 -3.92978300 -1.58589000

C -0.48826000 2.00440900 3.83922100

H 0.32341200 2.69691600 3.60010500

H -0.30827300 1.04906800 3.34019400

H -0.53695100 1.85871200 4.92289000

H -1.43295600 2.42678900 3.48571100

**TS1 (*F*_z1_=+0.004 a.u.)**

Fe -0.08214900 0.00195000 -0.00108700

O -0.11706400 -0.12192400 1.71904500

H 4.97879200 -0.04458500 -0.63697500

H -4.83508800 0.89493400 -1.45042500

H 4.28190200 2.57720600 -0.72439000

H -4.70501600 -1.78806600 -1.36614200

H 2.20310800 -4.48679800 -0.68900200

H -2.94413100 3.90749000 -1.40323000

H -0.47703600 4.96373900 -1.24822500

H -0.45510600 -5.01452800 -0.83881400

N 1.70446500 0.50055400 -0.42948400

N -1.87224900 -0.29007700 -0.56906400

N 0.26599300 -1.79040400 -0.49901300

N -0.59474700 1.74398600 -0.52121700

C 3.99226800 0.39081100 -0.56271300

C -3.98156400 0.29474800 -1.16663100

C 3.64003300 1.71580600 -0.60394600

C -3.91744600 -1.08694700 -1.12773700

C 2.77789000 -0.38217100 -0.46577900

C -2.67813600 0.79691100 -0.82683600

C 2.19868800 1.79700100 -0.53196500

C -2.56491700 -1.46008300 -0.77931200

N 2.69717300 -1.71175600 -0.47209200

N 1.48621000 2.92281600 -0.64706700

N -2.00644500 -2.66803900 -0.75655900

C 1.53816100 -2.36591400 -0.51138500

C -1.92497000 2.00137000 -0.80473000

C 0.15524400 2.88920000 -0.69418000

C -0.67984900 -2.80704200 -0.62869000

C 1.37746900 -3.79179900 -0.62776000

C -2.03651800 3.39567000 -1.11405700

C -0.76558600 3.94372000 -1.03644200

C 0.03110400 -4.05927600 -0.69830600

C -0.27096400 -2.45712700 2.63695900

H 0.59603400 -2.95987600 2.21373600

H -0.18898800 -1.22265700 2.15578700

H -1.22857900 -2.81754100 2.26710400

H -0.22659900 -2.27945600 3.71063400

**RC (*F*_z1_=+0.010 a.u.)**

Fe -0.55945600 0.05234000 0.46363300

O -0.79519200 -0.19593900 2.05140000

H 4.40080100 1.30617700 0.52083200

H -4.94831500 0.07492500 -2.04906800

H 3.07950200 3.67520800 0.61058800

H -4.12712200 -2.47391300 -2.23877400

H 2.92543100 -3.57733500 -0.47721400

H -3.97917100 3.29474000 -1.36181700

H -1.91907800 4.87364400 -0.67122300

H 0.54771100 -4.70309100 -1.13550800

N 1.08921500 1.00688800 0.39875300

N -2.12597700 -0.59395600 -0.40055000

N 0.32453300 -1.52382000 -0.13868600

N -1.43515200 1.69747900 0.08088100

C 3.33539800 1.48787000 0.53980800

C -4.08348400 -0.36927300 -1.57587500

C 2.67285200 2.67367000 0.59020800

C -3.66569800 -1.67319800 -1.67742200

C 2.33191300 0.43053800 0.40591100

C -3.09063200 0.33553800 -0.79230600

C 1.23894800 2.38896500 0.47576100

C -2.39358700 -1.78604400 -0.96557300

N 2.61843300 -0.86502800 0.25809200

N 0.30496300 3.31472700 0.34580500

N -1.55079400 -2.84250000 -0.96352900

C 1.67214600 -1.75578200 -0.04632900

C -2.69705300 1.64591700 -0.51359600

C -0.96717100 2.95818900 0.04410600

C -0.29213400 -2.68574000 -0.59650500

C 1.93469000 -3.14945300 -0.41615000

C -3.07060300 2.99916600 -0.85540400

C -2.01627900 3.81004100 -0.50380200

C 0.74407900 -3.71493100 -0.74313500

C 2.09808300 0.24050300 3.79509500

H 2.48812500 1.24015600 3.58493000

H 1.11945700 0.12496500 3.31964000

H 2.78649300 -0.50124700 3.38142400

H 2.01047000 0.09816000 4.87917000

**TS1 (*F*_z1_=+0.010 a.u.)**

Fe -0.46911700 -0.35396400 0.05391400

O -0.64472400 -0.69910500 1.83333300

H 4.63674800 -0.62680800 -0.15469700

H -4.74393400 1.15949300 -2.16535900

H 4.09448100 2.01468700 0.16340600

H -4.73114900 -1.49732900 -2.57205000

H 1.73930200 -4.74399300 -1.40472000

H -2.84691800 3.86530600 -1.37584600

H -0.37424000 4.71848300 -0.75334200

H -0.89680400 -5.07496800 -1.95849400

N 1.38776200 0.08292000 -0.10672600

N -2.19884600 -0.45054600 -0.72620200

N -0.12231000 -2.06384400 -0.73760500

N -0.82235800 1.50207800 -0.18431400

C 3.67699700 -0.13752000 -0.06413900

C -4.03648800 0.44118100 -1.77479900

C 3.40460400 1.18475700 0.09899500

C -4.03250100 -0.91540800 -1.98674200

C 2.39709000 -0.83274900 -0.21164600

C -2.84944900 0.75155900 -1.00876800

C 1.94879700 1.34775800 0.04620200

C -2.82681000 -1.45610800 -1.36304600

N 2.27968100 -2.13973900 -0.45970100

N 1.33179500 2.51453500 0.03881700

N -2.34018900 -2.70847000 -1.50183200

C 1.09723800 -2.68100000 -0.76247300

C -2.06917900 1.86322800 -0.69444300

C -0.00446100 2.56611600 -0.19637000

C -1.07277000 -2.95288800 -1.22496300

C 0.92172300 -4.05508300 -1.24534100

C -2.04225500 3.28322800 -0.94822100

C -0.77652600 3.72256700 -0.63011400

C -0.39690800 -4.22276400 -1.51946900

C 1.43622500 -0.86227500 3.21560400

H 1.96549900 -1.70674000 2.77528000

H 0.31845200 -0.76452300 2.47338300

H 1.09450100 -1.03326300 4.23758100

H 1.93842800 0.09303900 3.06491600

**RC (*F*_z1_=-0.002 a.u.)**

Fe 0.08660400 -0.30077100 0.31820400

O 0.23052400 -0.79523100 1.84582000

H 5.03858200 0.61163000 -0.52366700

H -4.85683400 0.25746300 -0.59913700

H 4.00249500 3.00496900 0.21856300

H -4.34527300 -2.26153300 -1.38314900

H 2.87729300 -4.00758900 -1.81040300

H -3.40700000 3.35805400 0.24884800

H -1.09258500 4.69390200 0.52676600

H 0.31493100 -4.88195100 -1.96973500

N 1.74787100 0.56088000 -0.03666800

N -1.70772500 -0.71597000 -0.15759200

N 0.63609800 -1.81788700 -0.69489800

N -0.70655800 1.42402900 0.42789800

C 4.01125500 0.86468600 -0.30263700

C -3.91012600 -0.26240400 -0.54888500

C 3.49250100 2.06289800 0.07366400

C -3.65062400 -1.55228900 -0.95517700

C 2.89964100 -0.08972900 -0.38868400

C -2.66541900 0.29332800 -0.06634500

C 2.04090600 1.89566700 0.22600100

C -2.22979500 -1.81034000 -0.73607000

N 3.01011600 -1.35913800 -0.79435100

N 1.18814000 2.87771400 0.46997200

N -1.50890000 -2.89399800 -1.12052000

C 1.93884300 -2.14296800 -0.95897700

C -2.08793300 1.52746300 0.27034300

C -0.14693800 2.64397400 0.48577000

C -0.18616700 -2.86810300 -1.09174500

C 1.96944500 -3.49999100 -1.51627400

C -2.41454900 2.93593700 0.32483000

C -1.23102600 3.62374500 0.46221200

C 0.68548900 -3.93935700 -1.59181300

C -0.80309600 1.97347600 3.79378900

H -0.04638900 2.73838800 3.59817500

H -0.50177300 1.03351300 3.32631200

H -0.90466600 1.83529300 4.87289600

H -1.76101300 2.30079900 3.38023700

**TS1 (*F*_z1_=-0.002 a.u.)**

Fe -0.05460700 -0.00984600 -0.04939400

O -0.09054000 -0.11662800 1.64959300

H 5.00896100 -0.02947000 -0.61758400

H -4.85086700 0.86907300 -1.34590500

H 4.30046600 2.58884400 -0.69887000

H -4.70172600 -1.81498200 -1.28385900

H 2.24215800 -4.49619100 -0.67308800

H -2.95914700 3.91550300 -1.29604800

H -0.49074700 4.97552900 -1.16887300

H -0.41391500 -5.03528000 -0.81365700

N 1.72831600 0.49694100 -0.48219500

N -1.84781100 -0.30656400 -0.60689900

N 0.29879700 -1.80291300 -0.53911800

N -0.57890000 1.73326100 -0.56432600

C 4.01723100 0.39901300 -0.58280900

C -3.97725700 0.27262200 -1.12123300

C 3.65879600 1.72239100 -0.62070200

C -3.90357700 -1.11105000 -1.09350100

C 2.80564300 -0.38227600 -0.50894500

C -2.66739200 0.78162200 -0.82761500

C 2.21527700 1.79812900 -0.57040700

C -2.53968000 -1.47908000 -0.79310000

N 2.72991700 -1.71221900 -0.51713300

N 1.49773700 2.92083600 -0.67862500

N -1.97375500 -2.68732800 -0.77800000

C 1.57224900 -2.37251600 -0.54718400

C -1.91810800 1.99022200 -0.80717500

C 0.16427700 2.88327600 -0.71543500

C -0.64759800 -2.82327100 -0.65707400

C 1.41493300 -3.80093000 -0.64410900

C -2.03983900 3.39277900 -1.06996700

C -0.76775100 3.94321300 -1.00687800

C 0.06941500 -4.07378100 -0.71039300

C -0.40037100 -2.40687900 2.64449700

H 0.45064900 -2.97103300 2.26846400

H -0.23657000 -1.21048100 2.12843100

H -1.36728000 -2.72419000 2.25912300

H -0.37599600 -2.18450100 3.71000300

**RC (*F*_z1_=-0.004 a.u.)**

Fe 0.09590300 0.23193600 0.37412300

O 0.15945900 0.28668900 1.98224600

H 5.13641000 0.25481800 -0.41059200

H -4.73983700 0.99934500 -0.89867000

H 4.39365900 2.85818900 -0.56003500

H -4.52666300 -1.67795300 -0.79814500

H 2.43791300 -4.26277800 -0.24268000

H -2.92849200 4.03543700 -1.02903000

H -0.47058000 5.12112400 -1.08897800

H -0.21002300 -4.84742400 -0.25378200

N 1.85496100 0.73432100 -0.15545400

N -1.73004300 -0.11681700 -0.03149300

N 0.46797800 -1.58954500 -0.04278900

N -0.48288000 1.97163400 -0.12940900

C 4.14343600 0.67896200 -0.36035200

C -3.85958400 0.42344900 -0.64848300

C 3.77088200 1.98402600 -0.43094200

C -3.75317500 -0.94856000 -0.60181300

C 2.92258300 -0.12029800 -0.20012000

C -2.56007900 0.96588100 -0.31754600

C 2.30655300 2.04012000 -0.32384300

C -2.37417400 -1.27652400 -0.24617400

N 2.88110700 -1.45669300 -0.15912600

N 1.57731300 3.13612200 -0.46081200

N -1.78584900 -2.49827800 -0.22496400

C 1.72464600 -2.12711000 -0.11421200

C -1.84031600 2.17154600 -0.37290500

C 0.22172300 3.07333500 -0.43641100

C -0.47160100 -2.61311500 -0.12542300

C 1.59542400 -3.58733000 -0.19449800

C -1.99408900 3.54937600 -0.78455700

C -0.73551400 4.10793000 -0.82052900

C 0.26888100 -3.88004900 -0.19677700

C -0.69011800 -2.87817100 3.21341700

H 0.07190700 -3.58358100 2.86981900

H -0.42333300 -1.86515100 2.90317100

H -1.65808100 -3.15410100 2.78623000

H -0.75051900 -2.92034100 4.30329900

**TS1 (*F*_z1_=-0.004 a.u.)**

Fe -0.04742200 -0.01459300 -0.06579000

O -0.08369000 -0.11465600 1.62730900

H 5.01680500 -0.02653200 -0.61074800

H -4.85527200 0.86152600 -1.31519700

H 4.30535200 2.59092200 -0.68677100

H -4.70122000 -1.82282600 -1.26264300

H 2.25246000 -4.50002000 -0.67430300

H -2.96349900 3.91772200 -1.25847000

H -0.49492300 4.97880000 -1.13759700

H -0.40306200 -5.04205400 -0.81162900

N 1.73470000 0.49459600 -0.49954000

N -1.84147200 -0.31216400 -0.62089800

N 0.30737900 -1.80778800 -0.55469400

N -0.57471800 1.72915900 -0.57873400

C 4.02385800 0.40002500 -0.58884700

C -3.97628200 0.26589200 -1.10997300

C 3.66380300 1.72309300 -0.62403700

C -3.90012000 -1.11834200 -1.08707800

C 2.81285700 -0.38347600 -0.52384000

C -2.66451900 0.77635100 -0.82947300

C 2.21966900 1.79719600 -0.58123800

C -2.53307500 -1.48525800 -0.80112500

N 2.73840500 -1.71370400 -0.53465400

N 1.50071100 2.91922700 -0.68613900

N -1.96515900 -2.69354300 -0.78926500

C 1.58118800 -2.37562500 -0.56252300

C -1.91619200 1.98632300 -0.80827300

C 0.16651800 2.88081500 -0.72035000

C -0.63896300 -2.82881200 -0.66972800

C 1.42488400 -3.80479900 -0.65502400

C -2.04098600 3.39167700 -1.05398800

C -0.76859400 3.94282500 -0.99412700

C 0.07958500 -4.07903300 -0.71961900

C -0.43379400 -2.38609500 2.65511100

H 0.41437800 -2.96691200 2.29819600

H -0.25003200 -1.20609000 2.12331600

H -1.40115600 -2.69741000 2.26577200

H -0.41701200 -2.14456900 3.71661800

**RC (*F*_z1_=-0.010 a.u.)**

Fe 0.15377700 -0.26658100 0.26963000

O 0.29487900 -0.69603700 1.75953000

H 5.13010900 0.55742800 -0.54658300

H -4.83841200 0.24806500 -0.33937500

H 4.11978700 2.97340600 0.18582300

H -4.39164400 -2.30765600 -1.06651400

H 2.85983400 -4.01405200 -1.73121500

H -3.27612200 3.35890500 0.50227800

H -0.93676700 4.66591500 0.73589600

H 0.27777600 -4.86636800 -1.82803500

N 1.83524800 0.58173600 -0.11569600

N -1.66494400 -0.67530900 -0.19793100

N 0.63136200 -1.71943200 -0.81763300

N -0.60009300 1.42852000 0.18543900

C 4.10006100 0.82917800 -0.36394800

C -3.88553000 -0.26067400 -0.38787100

C 3.59175300 2.04694600 0.00865500

C -3.65855600 -1.57354200 -0.76255400

C 2.99232700 -0.09140800 -0.46659800

C -2.61004700 0.31574500 -0.06222500

C 2.15899300 1.90464200 0.14330000

C -2.23511300 -1.81724900 -0.68238100

N 3.05178200 -1.35159600 -0.89162200

N 1.30006400 2.89821400 0.37859800

N -1.53017300 -2.87241800 -1.09342300

C 1.96500000 -2.09913100 -1.05267500

C -1.98844500 1.55833400 0.19495300

C -0.00819600 2.67151500 0.37915900

C -0.19689400 -2.81519100 -1.12338000

C 1.95813300 -3.45872500 -1.51383500

C -2.28473400 2.93700700 0.41166600

C -1.08200900 3.61551500 0.52574900

C 0.65498100 -3.89109100 -1.55335600

C -1.17639200 1.90118200 3.70523900

H -0.58540700 2.78882100 3.46562500

H -0.74422800 1.02453500 3.21489300

H -1.16901000 1.74647000 4.78540900

H -2.20680500 2.04293700 3.36857900

**TS1 (*F*_z1_=-0.010 a.u.)**

Fe 0.00837900 -0.63540500 -0.14632100

O -0.04204500 -0.90983900 1.54665200

H 5.01152300 0.16920100 -0.67361500

H -4.92842700 -0.14410900 -1.03905900

H 3.96942500 2.58908600 -0.03414300

H -4.41156900 -2.69193200 -1.73403200

H 2.85032900 -4.44569400 -2.00599400

H -3.44481800 3.03373100 -0.19735200

H -1.12434100 4.34882300 0.15666800

H 0.28196300 -5.29175200 -2.25623400

N 1.69676100 0.19506300 -0.44757100

N -1.75062700 -1.02241800 -0.74648500

N 0.58962000 -2.21235600 -1.04212300

N -0.76824500 1.08544300 -0.10050600

C 3.97144600 0.44162200 -0.56013700

C -3.96428700 -0.63438000 -1.03735100

C 3.44521800 1.66426900 -0.23191200

C -3.69919600 -1.94967500 -1.40046500

C 2.87145200 -0.48786800 -0.71226100

C -2.72280700 -0.04259800 -0.63556400

C 2.00377000 1.52885100 -0.17105400

C -2.28498500 -2.18390500 -1.23332500

N 2.97632700 -1.76570600 -1.09021100

N 1.14252900 2.52669300 0.03044900

N -1.55979100 -3.26797600 -1.55910300

C 1.91320900 -2.55371700 -1.26791300

C -2.14363600 1.20733400 -0.26816700

C -0.18405400 2.31942400 0.00815800

C -0.22877400 -3.26190600 -1.47127500

C 1.94003200 -3.90491600 -1.78616500

C -2.45539300 2.59934600 -0.15250800

C -1.25658300 3.28387900 0.02319700

C 0.64199600 -4.33356500 -1.90709900

C -0.57825400 0.96213500 2.98790000

H 0.38269600 1.40009400 2.74901400

H -0.65181700 -0.31865900 2.16683900

H -0.65544500 0.39267200 3.91189700

H -1.47019300 1.44255500 2.59717600

**P (*F*_z1_=-0.010 a.u.)**

Fe -0.04399700 0.04660800 0.17554000

O -0.47288100 -0.45213900 2.05583200

H 4.95877900 0.83564200 -0.16196600

H -4.97493600 0.40540300 -0.79255200

H 3.87833400 3.28309400 0.27206200

H -4.43365400 -2.18529700 -1.29534900

H 2.85412800 -3.91385000 -1.04927000

H -3.54661900 3.61141400 0.02688300

H -1.25452600 4.97374100 0.37335600

H 0.30331100 -4.79978200 -1.32932900

N 1.64351200 0.82890400 0.06255300

N -1.82299300 -0.45393800 -0.28770200

N 0.54671500 -1.58127800 -0.54400200

N -0.83093000 1.72227900 0.07607200

C 3.91442300 1.09883500 -0.06799700

C -4.01508900 -0.08700200 -0.71430700

C 3.36764300 2.33759600 0.15213500

C -3.73794200 -1.42044600 -0.97795100

C 2.82838600 0.14589400 -0.15408600

C -2.78155500 0.52976300 -0.30132300

C 1.92751800 2.19023600 0.20855300

C -2.32559300 -1.63520500 -0.73446800

N 2.93935700 -1.15161000 -0.44484200

N 1.04898000 3.18591800 0.30518300

N -1.57838200 -2.72914200 -0.96038300

C 1.88088800 -1.94776900 -0.62307000

C -2.20858300 1.81288000 -0.04788100

C -0.27347200 2.96517800 0.23181400

C -0.24729800 -2.68925600 -0.84401400

C 1.93487300 -3.35121800 -0.96175000

C -2.54774400 3.19767700 0.05519900

C -1.36227700 3.90713400 0.23137600

C 0.64332300 -3.79963100 -1.09752400

C 0.10113400 0.29935200 3.17466800

H 1.17869100 0.28716700 3.02499000

H -1.42534100 -0.58429100 2.18228900

H -0.15744500 -0.21738700 4.09997300

H -0.27921800 1.32243400 3.17266000

**Coordinates of the doublet state under the *F*_z2_**

**RC (*F*_z2_=+0.002 a.u.)**

O -1.85724900 -0.43904400 0.67797400

C -2.14566900 1.40460300 3.57074500

Fe -0.38693400 -0.28777600 0.02847700

H -0.63014800 4.67239600 -1.17533500

H 1.53973600 -4.93645200 -0.47091300

H -1.37647500 3.36661700 -3.43154600

H 2.43252500 -4.13471800 1.93407300

H 1.53689500 3.10022200 3.33374000

H 0.11498100 -3.88169200 -3.49679600

H -0.77555600 -1.75759200 -4.87873700

H 2.30238400 0.67857600 4.28879900

N -0.46592400 1.35715300 -0.92787700

N 0.49655100 -1.91705500 0.45592200

N 0.63254400 0.54567800 1.40628000

N -0.49727500 -1.18484800 -1.64346400

C -0.68032300 3.60785500 -1.35542300

C 1.32185000 -4.00264900 0.02867900

C -1.05803500 2.95401600 -2.48446800

C 1.78349700 -3.59323900 1.26008500

C -0.28817500 2.59449800 -0.36924100

C 0.52220400 -2.92493100 -0.50742100

C -0.90476900 1.51366600 -2.23991000

C 1.28170000 -2.24358800 1.49626900

N 0.21127400 2.86124800 0.84146500

N -1.06272600 0.57394700 -3.15670900

N 1.60974400 -1.39825600 2.50651100

C 0.67071300 1.89346800 1.64082000

C -0.04802400 -2.50255900 -1.71686900

C -0.77811100 -0.71899500 -2.87259500

C 1.30529400 -0.11298800 2.43105300

C 1.36440300 2.12000300 2.91228500

C -0.15120600 -2.91103300 -3.10208200

C -0.60361000 -1.82527200 -3.81357700

C 1.74230500 0.90634100 3.39266400

H -1.39780700 1.07197500 4.29596500

H -2.11350300 0.76262400 2.68683800

H -3.13963400 1.35583700 4.02410200

H -1.93126200 2.43669700 3.27962300

**TS1 (*F*_z2_=+0.002 a.u.)**

O -0.04389800 -0.11109000 0.18472800

C -0.06176400 0.34131600 2.65677200

Fe 1.48785000 -0.07460400 -0.58410300

H 1.39027100 4.79748200 -2.08538500

H 3.03735000 -4.87704600 -0.66904500

H 0.60944400 3.37276400 -4.25787700

H 3.92436500 -3.97850100 1.70444100

H 3.41807200 3.41960100 2.55701600

H 1.64633500 -3.93211700 -3.82530900

H 0.81431200 -1.87396200 -5.33935300

H 4.08813400 1.02920900 3.65908700

N 1.47214900 1.49928700 -1.65250100

N 2.34665000 -1.69486200 -0.07906900

N 2.54827300 0.78607300 0.72792400

N 1.40044800 -1.05953000 -2.19276000

C 1.31589200 3.72452400 -2.19236900

C 2.91251300 -3.88262500 -0.26292700

C 0.91833200 3.00494200 -3.28944600

C 3.37321400 -3.42184800 0.95929800

C 1.67997400 2.78470800 -1.15859600

C 2.27411500 -2.78060300 -0.92704100

C 1.02366000 1.59914000 -2.96471400

C 3.03855200 -2.01989100 1.06304800

N 2.17851700 3.09015400 0.03672000

N 0.81091200 0.59417000 -3.82071700

N 3.38611600 -1.13603100 1.99910800

C 2.59941800 2.16841400 0.90465500

C 1.71896400 -2.40685500 -2.18065800

C 1.03404800 -0.66953100 -3.46445700

C 3.15204500 0.17259300 1.82744200

C 3.23289100 2.42729300 2.17006800

C 1.48458200 -2.91330300 -3.50105400

C 1.05700200 -1.85444700 -4.28613400

C 3.56791400 1.21574800 2.72987800

H 0.52543200 -0.47697600 3.06837900

H -0.05802900 0.10393200 1.35404500

H -1.12727500 0.30673400 2.87824600

H 0.39997600 1.32261600 2.74264200

**RC (*F*_z2_=+0.004 a.u.)**

O -1.82030700 -0.51946900 0.76122900

C -2.26026200 1.64659700 3.38483300

Fe -0.37044700 -0.33967500 0.07212200

H -0.66869500 4.63770300 -1.06059500

H 1.61906200 -4.94479900 -0.55298100

H -1.44654700 3.35646300 -3.31986900

H 2.54433100 -4.17535900 1.85056100

H 1.57435700 3.01962800 3.39210100

H 0.12657900 -3.86196900 -3.52737600

H -0.81625900 -1.72770100 -4.85587200

H 2.38373000 0.59096300 4.29751400

N -0.48626900 1.31927900 -0.85566800

N 0.54290400 -1.96346700 0.45455300

N 0.66830300 0.48466600 1.44262300

N -0.50431100 -1.21020700 -1.61129200

C -0.71959200 3.57507600 -1.25124800

C 1.39275500 -4.02641100 -0.02907500

C -1.11315000 2.93439900 -2.38206700

C 1.87055700 -3.63276400 1.20218600

C -0.30571300 2.55127800 -0.28492100

C 0.56572200 -2.95322200 -0.52809100

C -0.94714700 1.49192500 -2.15834200

C 1.35222200 -2.29756600 1.47374400

N 0.21221800 2.80485100 0.92063600

N -1.11085800 0.56527300 -3.08682300

N 1.68503400 -1.46585400 2.49511900

C 0.69425300 1.82891800 1.69695700

C -0.03348200 -2.51824800 -1.71820300

C -0.80753500 -0.72680600 -2.82892000

C 1.36417800 -0.18291100 2.44552700

C 1.40354100 2.04332800 2.96069300

C -0.15062000 -2.90384800 -3.11038200

C -0.62952000 -1.81384400 -3.79443100

C 1.80319500 0.82566100 3.41612000

H -1.49889500 1.43385300 4.14109000

H -2.19649500 0.90639300 2.58323400

H -3.25275000 1.60875800 3.84331200

H -2.08790100 2.64384200 2.97140700

**TS1 (*F*_z2_=+0.004 a.u.)**

O -0.04610900 -0.15653000 0.19926400

C -0.09079800 0.49171200 2.62572700

Fe 1.48810300 -0.10386400 -0.57452900

H 1.35729100 4.78002600 -2.04630700

H 3.08768900 -4.88855500 -0.71244900

H 0.57740400 3.36259300 -4.22288500

H 3.97311100 -4.00627900 1.66758700

H 3.41292800 3.38247100 2.57761400

H 1.67740300 -3.92949500 -3.85243000

H 0.82006900 -1.86783900 -5.34460800

H 4.10063700 0.98760600 3.66461300

N 1.45780000 1.47784200 -1.63130400

N 2.36523000 -1.72030500 -0.08585800

N 2.54728100 0.75554100 0.74109800

N 1.40327800 -1.07577200 -2.18871800

C 1.28616900 3.70727600 -2.15694700

C 2.95231300 -3.90076500 -0.29370700

C 0.88911500 2.99226800 -3.25625100

C 3.41101400 -3.44791500 0.93187000

C 1.65962300 2.76319300 -1.12937200

C 2.30056900 -2.79798100 -0.94431500

C 1.00459800 1.58466100 -2.93891500

C 3.06255100 -2.04986900 1.05115600

N 2.16075200 3.06263400 0.06446600

N 0.79664000 0.58433700 -3.80263000

N 3.40402800 -1.17212500 1.99510200

C 2.59131900 2.13598900 0.92564200

C 1.73636700 -2.41859000 -2.19164100

C 1.03077000 -0.67812500 -3.45802500

C 3.15909000 0.13763500 1.83425300

C 3.22824800 2.39095200 2.18811800

C 1.50410100 -2.91606700 -3.51761200

C 1.06364800 -1.85616200 -4.29142400

C 3.57236700 1.17636000 2.74025400

H 0.51239200 -0.27799600 3.10259200

H -0.07370200 0.14986500 1.34126300

H -1.15661500 0.45201200 2.84503400

H 0.35008300 1.48613800 2.63425200

**RC (*F*_z2_=+0.010 a.u.)**

O -1.80977300 -0.51441900 0.82004700

C -2.26483900 1.60337100 3.41479400

Fe -0.38249100 -0.33778100 0.07809500

H -0.65461200 4.64031300 -1.08414700

H 1.64392900 -4.91683900 -0.58270500

H -1.43637500 3.34992700 -3.33569700

H 2.56683700 -4.15938500 1.82703000

H 1.57807200 3.03056100 3.37362800

H 0.14906500 -3.84452200 -3.53871600

H -0.79954500 -1.71323800 -4.86363200

H 2.39298000 0.60007900 4.28554900

N -0.49848100 1.31870900 -0.85168100

N 0.53880100 -1.95847500 0.45467800

N 0.66577400 0.48925700 1.44374100

N -0.52847500 -1.20800500 -1.60320200

C -0.71979600 3.57634900 -1.26210300

C 1.40280600 -4.00996100 -0.04526500

C -1.11518300 2.93380300 -2.39032100

C 1.87734900 -3.62068700 1.19159600

C -0.31400900 2.55535100 -0.28858400

C 0.56625400 -2.94346900 -0.53260700

C -0.95695400 1.49125200 -2.15657900

C 1.35086100 -2.29449900 1.47239900

N 0.19985100 2.81056100 0.91807700

N -1.12049300 0.56458100 -3.08303200

N 1.67825500 -1.46416500 2.50023300

C 0.68722300 1.83387900 1.69384900

C -0.03946700 -2.50924400 -1.72050300

C -0.81910100 -0.72252500 -2.82565900

C 1.35928700 -0.17828400 2.44643000

C 1.39653400 2.05106000 2.95271800

C -0.14287400 -2.89251500 -3.11755900

C -0.62373600 -1.80639300 -3.79997100

C 1.79822100 0.83049200 3.41145400

H -1.49589100 1.40780100 4.16931600

H -2.18299200 0.86437500 2.61235500

H -3.25946600 1.54618500 3.86967800

H -2.11136000 2.60123900 2.99569900

**TS1 (*F*_z2_=+0.010 a.u.)**

O 0.28046400 -0.20603000 1.08770800

C -1.73218300 1.00770100 0.23685100

Fe 1.70424500 -0.22046500 -0.02467300

H 1.50956800 4.77207900 -1.15371300

H 3.26283900 -4.89732700 -1.07651600

H 0.21200400 3.53790100 -3.19127100

H 4.72492900 -4.21836700 1.07902700

H 4.57528200 3.00947400 2.68551500

H 1.29999600 -3.72493700 -3.65601600

H 0.23925100 -1.53537900 -4.79354700

H 5.43214600 0.53292600 3.42621400

N 1.54286700 1.43889100 -0.97011200

N 2.60729800 -1.87625300 0.14491500

N 3.08480800 0.53628700 1.06431600

N 1.15964600 -1.06862900 -1.64240200

C 1.35413200 3.71428500 -1.31170100

C 3.20008800 -3.98082700 -0.50624100

C 0.69892600 3.09999500 -2.33040300

C 3.95037100 -3.63232800 0.60377000

C 1.90656800 2.66724700 -0.44749500

C 2.36030500 -2.85939500 -0.81136200

C 0.83376900 1.65051200 -2.12998800

C 3.59024800 -2.28064000 0.97503500

N 2.65808100 2.88292700 0.62079500

N 0.41526600 0.73998600 -3.00719000

N 4.19032300 -1.47663700 1.89292200

C 3.24581100 1.86566300 1.28703200

C 1.54160800 -2.37982500 -1.84793800

C 0.67582800 -0.55326300 -2.79959700

C 3.95317600 -0.17547200 1.89548600

C 4.23172900 2.04247300 2.34390100

C 1.14501000 -2.75871400 -3.19625700

C 0.60895100 -1.64463200 -3.78258300

C 4.66018200 0.79777700 2.71614700

H -1.39755800 2.04170400 0.16214000

H -0.69743100 0.35717500 0.70184000

H -2.51930400 0.84706600 0.97329800

H -1.93248400 0.54393000 -0.72792900

**RC (*F*_z2_=-0.002 a.u.)**

O -1.96031900 -0.00547300 0.47352400

C -1.80224800 0.11961700 3.98739900

Fe -0.44326500 -0.01939200 -0.07414400

H -0.30456500 4.82673800 -1.66783100

H 1.14028900 -4.82477700 -0.07219200

H -1.02708700 3.39277500 -3.85444100

H 1.99084500 -3.87583700 2.29389400

H 1.53580900 3.49608200 3.06375600

H -0.06634000 -3.93601000 -3.24970300

H -0.73099000 -1.88072500 -4.85125000

H 2.09364600 1.11778400 4.23982500

N -0.35654700 1.53937300 -1.16509700

N 0.29206400 -1.66483000 0.53534000

N 0.56489500 0.85439400 1.28503700

N -0.53611600 -1.04830300 -1.67172700

C -0.40771900 3.75582300 -1.77229200

C 0.97881100 -3.83441400 0.33033700

C -0.77338900 3.03646700 -2.86584300

C 1.41900600 -3.35197300 1.54068200

C -0.12690800 2.80280200 -0.69265700

C 0.28645000 -2.75181100 -0.33622800

C -0.72551200 1.61370600 -2.50479100

C 1.00995400 -1.95104600 1.63581800

N 0.32847700 3.13634400 0.51896800

N -0.90900900 0.61278800 -3.35042900

N 1.36144300 -1.04844800 2.58119900

C 0.68076900 2.21238100 1.41808500

C -0.19280800 -2.39732000 -1.60742900

C -0.73177100 -0.67170900 -2.94657000

C 1.14608100 0.24244700 2.39248800

C 1.32477500 2.49829200 2.70595500

C -0.26900500 -2.91604200 -2.95331400

C -0.60591300 -1.86372000 -3.77750300

C 1.60145100 1.30753200 3.29636400

H -1.16694200 -0.70839500 4.31264100

H -1.93620700 0.07960100 2.90366400

H -2.77466600 0.04415000 4.48040800

H -1.33125200 1.06586400 4.26746500

**TS1 (*F*_z2_=-0.002 a.u.)**

O -0.03287200 -0.08027600 0.17208800

C -0.05036400 0.20927200 2.67298200

Fe 1.49212000 -0.04955000 -0.58986200

H 1.41896500 4.81252600 -2.11260300

H 2.99011900 -4.86781200 -0.62624700

H 0.63135500 3.38422000 -4.28305800

H 3.88578500 -3.95154400 1.73734200

H 3.42657500 3.45136900 2.54309200

H 1.61294900 -3.93621200 -3.79659000

H 0.80156500 -1.88089200 -5.33233500

H 4.08262200 1.06643900 3.65521800

N 1.48595400 1.51879400 -1.67005800

N 2.33638000 -1.67108700 -0.07523700

N 2.55328000 0.81255200 0.71598600

N 1.39830400 -1.04530200 -2.19778300

C 1.34320200 3.73958400 -2.21982800

C 2.88003100 -3.86557700 -0.23541500

C 0.94231300 3.01670000 -3.31510600

C 3.34670800 -3.39687200 0.98206500

C 1.70061600 2.80220000 -1.18277000

C 2.25334700 -2.76489700 -0.91171400

C 1.03931400 1.61230100 -2.98623000

C 3.02553400 -1.99171700 1.07055500

N 2.19982200 3.11350400 0.01342800

N 0.82100100 0.60408700 -3.83671600

N 3.37976300 -1.10216400 1.99838900

C 2.61132500 2.19688900 0.88699100

C 1.70366800 -2.39620600 -2.17052200

C 1.03503800 -0.66217200 -3.46951200

C 3.15262500 0.20366200 1.82022400

C 3.24345400 2.45906700 2.15555600

C 1.46456500 -2.91150500 -3.48418500

C 1.04697000 -1.85305900 -4.27977600

C 3.57107800 1.25131800 2.72094700

H 0.54928900 -0.62450700 3.03260500

H -0.04482200 0.06021100 1.36889400

H -1.11446800 0.14267400 2.89364600

H 0.39364700 1.19085900 2.82552100

**RC (*F*_z2_=-0.004 a.u.)**

O -1.96517000 0.02013400 0.44421300

C -1.76933000 0.03819700 4.00995200

Fe -0.44211600 -0.00209000 -0.08440600

H -0.29095300 4.83670600 -1.69110500

H 1.10513500 -4.81995600 -0.04017400

H -1.00922300 3.39782800 -3.87688300

H 1.95107700 -3.86061100 2.32318700

H 1.52629500 3.51895400 3.05584000

H -0.08441300 -3.94001500 -3.23342300

H -0.73388600 -1.88979500 -4.85039000

H 2.06923200 1.14408500 4.24305600

N -0.34507000 1.55145400 -1.18267600

N 0.28106400 -1.64767800 0.53716800

N 0.56183700 0.87370200 1.27574800

N -0.52978100 -1.03889000 -1.67847100

C -0.39206500 3.76546600 -1.79495500

C 0.95265300 -3.82425700 0.35248300

C -0.75549700 3.04287500 -2.88768000

C 1.39050900 -3.33750600 1.56092900

C -0.11506100 2.81521700 -0.71194300

C 0.27262600 -2.73961300 -0.32618100

C -0.71053400 1.62100000 -2.52316800

C 0.99162800 -1.93100100 1.64404100

N 0.33644500 3.15238600 0.49994600

N -0.89576900 0.61714300 -3.36565900

N 1.34479600 -1.02616200 2.58365000

C 0.68113800 2.23191800 1.40575600

C -0.19877800 -2.38995900 -1.60352900

C -0.72443000 -0.66826000 -2.95522000

C 1.13435200 0.26495300 2.39004300

C 1.31742600 2.52086500 2.69763100

C -0.27795900 -2.91606700 -2.94424300

C -0.60730100 -1.86498200 -3.77678300

C 1.58670000 1.33193800 3.29413600

H -1.14402000 -0.80403900 4.31704000

H -1.91118100 0.02168900 2.92647800

H -2.73913600 -0.03331300 4.50792900

H -1.28505600 0.97241700 4.30707300

**TS1 (*F*_z2_=-0.004 a.u.)**

O -0.02575300 -0.06301400 0.16806200

C -0.04424000 0.14593500 2.68021500

Fe 1.49552200 -0.03689100 -0.59243000

H 1.43639200 4.81963600 -2.12825600

H 2.96124300 -4.86411100 -0.60237600

H 0.64244100 3.38901900 -4.29644100

H 3.86232300 -3.93909900 1.75581800

H 3.43551800 3.46751500 2.53401700

H 1.59096000 -3.93952400 -3.78064400

H 0.78993300 -1.88596500 -5.32766800

H 4.08183100 1.08518300 3.65309100

N 1.49452900 1.52799100 -1.67989000

N 2.33198500 -1.65878300 -0.07350000

N 2.55860400 0.82592100 0.70918300

N 1.39615700 -1.03868100 -2.19982100

C 1.35942200 3.74667700 -2.23512500

C 2.86030700 -3.85767700 -0.21995500

C 0.95545500 3.02195500 -3.32883100

C 3.33026900 -3.38513600 0.99503100

C 1.71399600 2.81068900 -1.19621900

C 2.24151900 -2.75712500 -0.90329400

C 1.04770000 1.61818400 -2.99712800

C 3.01841800 -1.97773300 1.07483900

N 2.21426600 3.12498800 -0.00028400

N 0.82478900 0.60810000 -3.84461800

N 3.37653100 -1.08555100 1.99842500

C 2.62108500 2.21103200 0.87679700

C 1.69393300 -2.39125900 -2.16457700

C 1.03348900 -0.65925400 -3.47150800

C 3.15451700 0.21929600 1.81647400

C 3.25230600 2.47513300 2.14678600

C 1.45060900 -2.91164800 -3.47454600

C 1.03808700 -1.85364300 -4.27565200

C 3.57501300 1.26906700 2.71596000

H 0.55900400 -0.69579400 3.01474300

H -0.03709800 0.04008400 1.37815700

H -1.10790300 0.06670800 2.89899900

H 0.39359700 1.12465500 2.86614300

**RC (*F*_z2_=-0.010 a.u.)**

O -1.98860800 0.06567800 0.31650600

C -1.63105400 -0.10882100 4.19564700

Fe -0.44393000 0.03023800 -0.13937700

H -0.26829500 4.85778700 -1.75132100

H 1.00905700 -4.81551400 0.01578800

H -0.94610900 3.41515100 -3.95099500

H 1.81152900 -3.83833900 2.38749500

H 1.44800700 3.55768200 3.04674200

H -0.10146200 -3.94979100 -3.22955600

H -0.70008400 -1.90813800 -4.88834000

H 1.94338900 1.18819600 4.25838400

N -0.31146400 1.57454400 -1.24969500

N 0.24099500 -1.61617600 0.51489200

N 0.52617800 0.90794300 1.24084300

N -0.49657500 -1.02062800 -1.73104900

C -0.35546200 3.78568500 -1.86141800

C 0.87588400 -3.80813500 0.38471100

C -0.69789600 3.05872600 -2.96008300

C 1.29117400 -3.31551000 1.59640500

C -0.09324400 2.83786000 -0.77251600

C 0.23753600 -2.71625500 -0.32913100

C -0.65596300 1.63717900 -2.59459300

C 0.91830900 -1.89488000 1.64838300

N 0.33266500 3.18018500 0.44616700

N -0.83482600 0.62926500 -3.43663400

N 1.26436500 -0.98831800 2.57825700

C 0.65007200 2.26689200 1.37064400

C -0.19827900 -2.37515900 -1.63032200

C -0.67904900 -0.66063400 -3.01365200

C 1.06453700 0.30518000 2.37700400

C 1.25078500 2.56003900 2.68031000

C -0.27309800 -2.91651100 -2.95818600

C -0.57692400 -1.86559900 -3.81369000

C 1.49635200 1.37362900 3.29098900

H -0.95504000 -0.92491800 4.46165800

H -1.81711100 -0.10587500 3.11740400

H -2.57440400 -0.23934900 4.72944400

H -1.18577300 0.84142700 4.50229200

**TS1 (*F*_z2_=-0.010 a.u.)**

O 0.01368200 -0.10525400 0.20472700

C -0.02339400 0.25004400 2.73045900

Fe 1.48780700 -0.05503600 -0.59271400

H 1.39693000 4.81164700 -2.09620400

H 2.97466700 -4.87946700 -0.62321100

H 0.62021000 3.39488600 -4.28581500

H 3.86930600 -3.95543900 1.73984200

H 3.40522300 3.43859400 2.55535900

H 1.63344200 -3.94334500 -3.80253400

H 0.83392400 -1.88240700 -5.35461900

H 4.06036100 1.05682700 3.66129300

N 1.47173100 1.51838200 -1.68060100

N 2.32635800 -1.67796100 -0.08309200

N 2.52964200 0.80484200 0.71171300

N 1.38470300 -1.04976600 -2.21527200

C 1.33271000 3.73845900 -2.21866000

C 2.87382700 -3.87161300 -0.24207700

C 0.93795100 3.01929100 -3.32107400

C 3.34146400 -3.40191800 0.97448600

C 1.68718900 2.79480200 -1.18574500

C 2.24608700 -2.77157000 -0.92022400

C 1.03606100 1.61221300 -3.00103800

C 3.01704900 -1.99626700 1.05884300

N 2.18761800 3.10536400 0.01114700

N 0.82946800 0.60802400 -3.86148100

N 3.37118300 -1.10563800 1.98648700

C 2.59649900 2.18854200 0.88378400

C 1.70206900 -2.40188400 -2.18146400

C 1.04568400 -0.66377400 -3.48749000

C 3.13877100 0.19374100 1.81632900

C 3.23091300 2.44830900 2.15632100

C 1.48345500 -2.91557600 -3.49526900

C 1.07356400 -1.85226700 -4.29822900

C 3.55766000 1.24233700 2.72083800

H 0.56495200 -0.59090600 3.09379000

H 0.01302600 0.06983200 1.36704900

H -1.09686000 0.18640500 2.90300500

H 0.42182200 1.23442500 2.86132000
